# Emerging nano sensing technologies for the detection of biological and chemical food toxins

**DOI:** 10.1039/d5ra06960b

**Published:** 2025-11-04

**Authors:** Mohsin Javed, Afzal Shah, Sidra Nasir, Sidra Pervaiz, Hazrat Hussain

**Affiliations:** a Department of Chemistry, Quaid-i-Azam University Islamabad 45320 Pakistan afzals_qau@yahoo.com; b Department of Applied Chemistry, Government College University Faisalabad 38040 Pakistan; c Department of Chemistry, Government College University Faisalabad 38040 Pakistan

## Abstract

This document presents advancements in contemporary detection technologies aimed at ensuring food quality and safety. It explores a range of sophisticated sensing platforms designed to identify harmful substances in food products. The integration of nanomaterials and microfluidics has significantly enhanced these detection techniques, resulting in improved sensitivity, specificity and response times. Additionally, the document emphasizes the importance of developing portable and user-friendly sensing devices, which not only facilitate practical applications of these technologies but also address the challenges and potential solutions associated with their implementation. Emerging trends, including smartphone-based detection systems and the incorporation of artificial intelligence for data analysis, are also examined, showcasing their potential to revolutionize food safety protocols. This review emphasizes the critical importance of advanced sensing technologies in mitigating risks associated with foodborne toxins, ensuring the integrity of the global food supply chain, and highlighting the necessity for ongoing research and development to meet the dynamic challenges of food safety. It offers a comprehensive analysis of the sources, toxicity characteristics, and detection challenges linked to various classes of toxins, followed by an overview of innovative sensor-based techniques for detecting food toxins. By consolidating existing knowledge, this comprehensive review establishes a foundation for future improvements in food quality and safety measures.

## Abstract 2

This document presents advancements in contemporary detection technologies aimed at ensuring food quality and safety.

## Graphical abstract

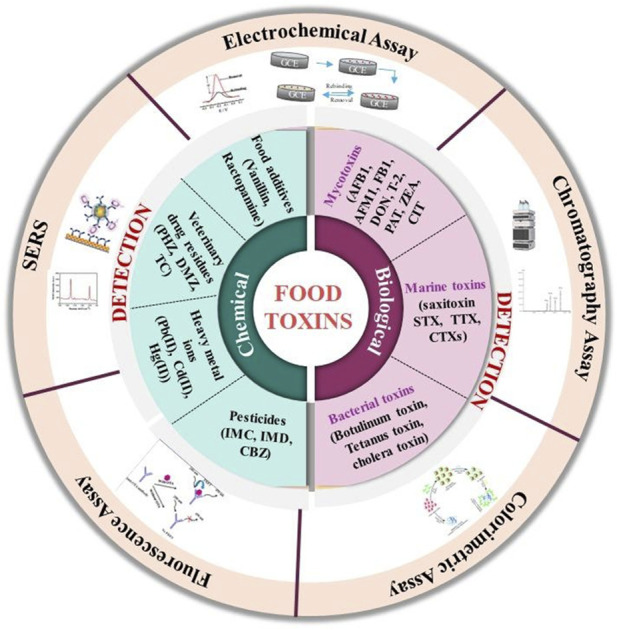

## 1. Introduction

Innovative technologies, including electrochemical and optical sensing systems, microfluidic platforms, and nanomaterial-based biosensors, are being developed to enable the rapid, accurate, and on-site detection of biological and chemical contaminants in food. Advancements in sensing technologies play a critical role in achieving Sustainable Development Goal 2 (Zero Hunger) by ensuring that food distributed globally is safe. By reducing exposure to hazardous pathogens, mycotoxins, heavy metals, and pesticide residues, these technologies are crucial for achieving SDG 3 (Good Health and Well-being). With contaminated food affecting millions each year and being a significant contributor to global illness, the integration of advanced sensing technology into regular food monitoring is crucial for preventing outbreaks, reducing healthcare costs, and enhancing consumer confidence. As the global community works towards meeting the SDGs by 2030, investing in next-generation food toxin detection systems is imperative for safeguarding food safety.

Toxins are produced by various organisms, including bacteria, algae, fungi, plants, and animals, for purposes such as competition, defense, or predation. Toxins do not pose a threat to the organisms that produce them; however, they can be detrimental to other creatures. Toxins have the potential to elicit an immune response.^1^ A notable example of such toxins is snake venom, which causes critical pathologies when injected through a bite. Aflatoxin is the most commonly found toxin in stored moldy grains, nuts, and fruits. Warm and humid conditions promote the growth of fungi that produce this toxin.^2^ Environmental pollution and the application of agricultural chemicals further exacerbate the generation of toxins. While these agents do not naturally occur in food, they can contaminate the food supply through environmental pollution.^3^ For instance, crops or animals absorb heavy metal ions from soil or water, and these ions then make their way into the food chain.^4^ Exposure to toxins through ingestion, inhalation, or direct contact can lead to a range of adverse health effects, which vary depending on their physical, chemical, or biological characteristics.^5^ Several chemical toxins have been reported to cause cellular death by blocking cellular metabolism or impairing nerve impulse transmission.^6^

The availability of toxin-free food is crucial for survival and promoting overall well-being. Technological innovations in food science have enhanced food production and processing systems, facilitating the global distribution of food to support population growth.^7^ Precision agriculture and genetically modified organisms have contributed to increased food production and improved quality in agricultural systems. The unintentional entry of trace amounts of chemical substances into food can pose significant health risks to humans, underscoring the urgent need for rigorous food safety measures.^8^ As the food industry evolves, it faces critical food safety challenges that represent a substantial global concern for both public health and economic stability. Food security and safety have become universal priorities due to international food adulteration scandals. Adequate food safety protection relies on stringent regulatory frameworks and comprehensive monitoring systems to safeguard public health and uphold consumer trust in national food supplies.^9^

The concept of food extends beyond nutritional and cultural aspects to encompass the essential protection of human wellbeing. Food safety control must focus on monitoring food quality and detecting potential contaminants. The processes of physical, biological, and chemical contamination are primarily associated with the methods used for transporting and storing food products.^10^ Pathogenic microorganisms and microbial toxins are the most significant factors in compromising food safety among all the factors that lead to food contamination.^11^ In 2010, the WHO reported that foodborne pathogens caused approximately 600 million illnesses and resulted in around 420 000 fatalities. In recent years, there has been a significant rise in foodborne illnesses, impacting all developed countries. Annually, 9.4 million cases of foodborne diseases are linked to recognized pathogenic agents and their toxins, which include 24 confirmed agents.^12^ In contrast, around 38.6 million cases arise from unidentified agents and unpredictable distribution systems. The economic impact of these foodborne illnesses is substantial, totaling $78 billion annually, encompassing costs related to healthcare, unemployment, product recalls, bankruptcies, and legal issues.^13^ A significant number of foodborne illnesses are caused by microbial toxins, including botulinum toxin, staphylococcal enterotoxin, and various enterotoxins, as well as mycotoxins produced by fungi and toxins found in seafood. Even minimal exposure to these toxic substances can lead to severe health complications for consumers.^14,15^

Food safety depends on continuous monitoring of biological and chemical toxins. Among these, Clostridium botulinum is known for producing botulinum toxin, while certain fungi, such as Aspergillus and Fusarium, are responsible for producing mycotoxins.^15^ The consequences of exposure to foodborne toxins can be severe, leading to a range of illnesses from food poisoning to chronic health issues, and in extreme cases, even death. Food products can become contaminated with chemical toxins, including pesticides, heavy metals, and industrial chemicals, which infiltrate the food supply through environmental pollution, agricultural practices, and food processing methods. Of particular concern are persistent organic pollutants and endocrine-disrupting chemicals, which can accumulate in the human body and disrupt hormonal functions, leading to significant health risks, including the development of cancers and reproductive issues.^16^

Monitoring the presence of toxins is crucial for safeguarding human health. Various testing methods and stringent quality control measures facilitate the identification of potential contamination risks. Regulatory bodies such as the WHO and the Food and Agriculture Organization (FAO) have established maximum permissible levels of toxins in food production. Advances in detection technologies, such as mass spectrometry and chromatography, have significantly enhanced the capability to identify and quantify these toxins, thereby improving safety standards and regulatory compliance. Maintaining rigorous monitoring and strict food regulations is vital to prevent unknown toxins from jeopardizing the food supply. Identifying pathogenic agents and their toxins is essential for addressing food safety challenges.^17^ The protection of public health and the preservation of consumer trust in the food supply rely heavily on robust food safety regulations and comprehensive monitoring systems, which should encompass all facets of nutritional heritage and health protection.^18^

Research on biological toxins primarily focuses on low-molecular-weight compounds, such as mycotoxins, which are often produced by fungi under suitable environmental conditions. These biotoxins can be found in various sources and have the potential to contaminate food, animal feed, and seafood during harvesting, processing, storage, and transportation.^19^ The presence of biological toxins at harmful levels can lead to food poisoning, particularly when used as agents in biological warfare. Notably, aflatoxin B1 (AFB1), a type of mycotoxin, is linked to liver cancer, affecting approximately 28.2% of diagnosed patients.^19^ Additionally, biotoxins are categorized based on their sources, with marine toxins including shellfish toxins, ciguatoxins, and tetrodotoxins, which pose significant health risks. Marine pollutants can enter the food chain, resulting in severe consequences, such as shellfish poisoning, which claims between 750 and 7500 lives globally each year. Bacterial toxins also contribute to foodborne illnesses by inhibiting protein synthesis and causing neurotoxicity. Notably, Clostridium botulinum produces the highly lethal botulinum toxin, which can cause death at doses as low as 100 ng.^20,21^ When these toxins cannot be effectively reduced or eliminated, it becomes essential to restrict their consumption.^22^

Recent advancements in the detection of biological and chemical toxins in food have been significant, driven by technological innovations and a heightened emphasis on public health safety. This review explores the latest detection methods, utilizing data from 2020 onward, and highlights advanced sensing protocols that improve sensitivity, specificity, and detection speed through developments in nanomaterials and microfluidics. It also addresses the role of electrode modifiers in facilitating electron transport between transducers and food toxins. Furthermore, the emergence of portable and user-friendly sensing devices is emphasized, enabling on-site testing and real-time monitoring, which ultimately bolsters food safety standards and encourages proactive strategies against foodborne illnesses. As the domain of food toxin detection evolves, this review serves as an essential reference for researchers, industry stakeholders, and regulatory bodies committed to protecting public health. It identifies prevalent detection challenges and offers adaptable solutions, concluding with a focused gap analysis and a roadmap for research priorities aimed at expediting industry and regulatory integration. The review explicitly connects theoretical sensing principles to practical applications by examining how factors such as matrix effects, sample preparation, analytical specificity, and validation processes influence the relevance of ultra-low limits of detection reported under controlled conditions for real-world food testing.

## 2. Sensors and their types

Sensors have become indispensable in modern operations, providing critical data that enhances security protocols and improves efficiency across various industries.^23^ Healthcare relies on sensors to track patient vital signs, as this enables the prompt detection of health issues, leading to enhanced patient recovery.^24^ Sensors enable precise farming to achieve better water optimization while monitoring the soil ecosystem and advancing agricultural production. Sensors play a crucial role in environmental monitoring systems, assessing air and water quality, and contributing to the resolution of pollution and effective climate change management.^25^ Sensors in the food industry monitor contaminants for both safety and quality purposes. The applications demonstrate the necessity of sensors that deliver real-time information to boost automation and upgrade system operational excellence. As advancements in technology continue, the role of sensors will remain critical in advancing safety protocols, streamlining processes, and supporting sustainable growth. In 132 AD, Zhang Heng constructed the seismoscope, which became the initial sensing device for detecting earthquakes.^26,27^ Food detection sensors are now essential for monitoring contaminants and evaluating the quality of food products in food safety procedures.^28^ The Toronto-based startup TellSpec created the first food-specific detector device, the TellSpec. Raman spectroscopy enables this device to analyze food through scans that reveal allergens, chemicals, nutrients, calories, and ingredients. The device TellSpec was developed following a food allergy incident involving the founder's daughter to provide consumers with precise information about food contents. The SCiO helps users select healthier food options, serving as a handheld molecular sensor that utilizes near-infrared light to identify molecular signatures in food.^27^ The protection of public health depends on strict food monitoring and thorough regulations, as consumer trust in the food supply requires this level of security. The evolution of sensor technology enhances our ability to detect and neutralize foodborne threats, thereby creating a more secure and diverse food supply chain. A variety of sensors are used to detect biological and chemical toxins present in food, as discussed *vide infra*.

Biosensors serve as analytical devices that combine biological recognition components with transduction systems for detecting specific substances, such as food toxin. Each biosensor contains one or more biological recognition components, including enzymes, antibodies, nucleic acids, or microorganisms, to detect the target substance.^28^ The biological interaction between recognition elements produces a detectable signal, which is then translated by the transducer into electrical, optical, or thermal outputs. A biosensor operates through four distinct operations: starting with the binding of the target analyte to its biological recognition component, followed by signal generation and transduction, and finally signal processing. The sequential operational steps enable biosensors to perform quick and precise detection of analytes.^29,30^ Biosensors play a crucial role in ensuring food safety and quality by detecting toxins. Modern biosensors can detect a wide range of toxic compounds, including pathogens, microbial toxins, pesticides, and heavy metals. Biosensors provide immediate monitoring data, enabling the detection of contaminated food products and helping to prevent dangerous consumption and reduce the risk of food-related illnesses.^31,32^ The application of nanotechnology and biotechnology has sparked the development of biosensors with greater accuracy and sensitivity, which now serve as fundamental tools in the food industry. Through continuous monitoring, these devices verify that products from production zones meet safety regulations and quality requirements as they are transported to storage and distribution points. Biosensors are essential for fostering consumer confidence in the safety of the food supply.

### 2.1 Electrochemical sensors

Electrochemical sensors utilize electrical signals to transform chemical information, enabling the detection and measurement of food toxins. These devices employ three principal sensing methods: potentiometry, amperometry, and voltammetry.^33^ The potentiometric sensor determines electrode potential disparity while sustaining a steady current flow in pH meter applications. Amperometric sensors track the constant voltage-controlled oxidation or reduction current to detect analytes. A changing voltage in voltammetric sensors produces measurable current fluctuations, enabling the detection of complex analyte combinations.^34^ When operating electrochemical sensors, the target analyte engages with a chemically sensitive layer attached to the electrode surface. An interaction occurs between the target molecule and the chemically sensitive layer, resulting in modifications to the electrical features that are measured as a distinct signal.^35,36^ These sensors obtain enhanced sensitivity and selectivity when their surfaces are modified with specific nanomaterials, enzymes, polymers and specialized recognition elements.^37^ Assessing food toxins through electrochemical sensors relies on detecting pesticides, heavy metals, and microbial toxins. Electrochemical sensors offer high precision and fast responsiveness, while providing portability benefits that support real-time testing across the entire food distribution chain, from factory production to end-user consumption.^36^ The sensors provide prompt information about toxins to ensure food safety, protect public health, and maintain consumer trust. The advancement of materials science and nanotechnology helps improve electrochemical sensors, thereby extending their usefulness in preserving the food supply.

### 2.2 Fluorescent sensors

Fluorescent sensors serve as analytical devices that detect specific substances using fluorescence-based methods, including the assessment of toxins in food products. Fluorescent sensors utilize specific wavelength-stimulated fluorescent dyes and probes that emit light. The intensity of fluorometric light detection and the variations in wavelength correlate directly with the concentration of the target analyte, enabling quantitative analysis.^38,39^ Fluorescent sensing utilizes three fundamental analytical methods: fluorescence resonance energy transfer (FRET), time-resolved fluorescence, and fluorescent quenching. The energy exchange between two fluorophores under FRET detection yields results whose intensity depends on the distance between them, making it suitable for detecting molecular interactions. Time-resolved fluorescence enhances sensitivity by separating background noise and measuring the emission signal over time. The structure of a fluorescent quencher causes a decrease in fluorescence intensity, allowing researchers to detect specific analytes. Flexible fluorescent analytical sensors prove irreplaceable during analyte monitoring because they offer exceptional sensitivity, speed, and high selectivity performance.^40–42^ These detectors demonstrate performance in identifying various substances, including infectious agents, toxic chemicals, microbial toxins, heavy metals, and pesticides. Fluorescent probes serve as targeted detectors for DNAs, bacterial toxins, such as botulinum toxin, and aflatoxins produced by molds, facilitating prompt detection and ongoing monitoring of these harmful substances.^43–46^ Fluorescent sensors used for food protection prevent contaminated food from being consumed, thereby protecting public health while preserving consumer confidence. Research in nanotechnology and biotechnology has significantly enhanced the capabilities of fluorescent sensors, allowing for the accurate detection of minute quantities of toxins.

### 2.3 Optical sensors

Optical sensors detect variations in light to identify specific substances by measuring their concentrations.^47^ The sensor mechanism utilizes a combination of absorption, fluorescence, phosphorescence, and reflectance processes to analyze the characteristics of various analytes. The functioning system of optical sensors creates a photon emission from a light source that interacts with the target analyte.^48^ The photodetector detects changes in light properties stemming from the interaction between the light source and its target element. The resulting optical signal provides a precise measurement that correlates directly with the concentration of the substance being analyzed. These sensors are particularly effective in detecting food toxins, thanks to their high sensitivity, remarkable selectivity, and swift response times.^49^ They are capable of identifying a wide range of biological and chemical contaminants, including pathogenic agents, toxic microbial substances, insecticides, and heavy metals. Specifically, fluorescence-based optical sensors employ fluorescent dyes in conjunction with quantum dots, which emit light upon binding to their target toxins, facilitating real-time detection and early identification of these harmful substances. Additionally, surface plasmon resonance (SPR) sensors can detect anionic substances at low concentrations by monitoring changes in the refractive index at the sensor surfaces after the binding of toxin-recognition elements.^50^ Optical sensors play a crucial role in food safety by enabling continuous monitoring of food products throughout production, distribution, and consumption. By providing accurate and timely information about the presence of contaminants, these sensors help protect public health and maintain confidence in the food industry.

### 2.4 Spectrometric and chromatographic sensors

Spectrometric and chromatographic sensors serve as fundamental analytical tools that help determine the identification and quantity of food toxins. Mass spectrometry (MS) and infrared spectroscopy (IR) serve as spectrometric sensors due to their ability to detect interactions between electromagnetic radiation and matter.^51^ Mass spectrometry determines the presence of chemicals by measuring their mass-to-charge ratio. In contrast, infrared spectroscopy determines chemical composition by analyzing the IR light absorption patterns of molecules.^52^ Chromatographic sensors separate mixtures into individual components, such as gas chromatography (GC) and high-performance liquid chromatography (HPLC). Separating volatile compounds occurs through GC methods, whereas HPLC analyzes all nonvolatile substances. The separation process in both methods utilizes fixed environments for each phase to determine how analytes bind with each station, which in turn defines their moving locations. Sample preparation followed by separation, detection, and data analysis comprises the sensor's operational procedure. Mass spectrometry involves applying ions to samples, followed by separation using a mass analyzer, and then detecting their ionization patterns to produce mass spectral output data.^53^ When a sample absorbs IR light in infrared spectroscopy, the resulting spectrum provides structural information about the molecules. The basis of chromatographic systems involves placing a sample into a column that uses monitored detectors, including mass spectrometers or UV detectors, to detect separated analytes.

### 2.5 Colorimetric sensors

**Fig. 1:**
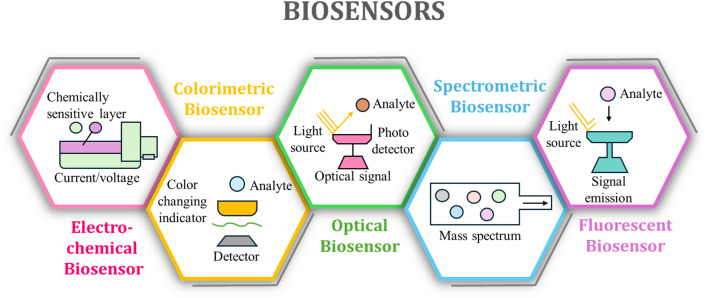
Biosensor technologies employed in food toxins detection, categorized by their working principles and analytical signals.

Colorimetric sensors sense discrete substances through visible color alterations.^54^ A color change occurs visibly when these sensors detect target analytes based on chemical processes that produce observable changes in color.^55,56^ The degree and tone of the created color directly correspond to the amount of analyte present, which enables quantitative examination. The function of colorimetric sensors depends on a recognition element, such as a chemical reagent or enzyme, that triggers a color reaction when it interacts with the target analyte. A spectrophotometer or direct human observation can effectively monitor color changes, providing both qualitative and quantitative data. The wide application of colorimetric sensors in food safety necessitates their ability to detect various contaminants, including pesticides, heavy metals, and microbial toxins, through the provision of speedy and cost-effective quality monitoring of food products. Comprehensive overview of biosensor technologies employed in food toxins detection, categorized by their working principles and analytical characteristics is given in Fig. 1 that systematically compares five major biosensor types: (1) electrochemical biosensors, utilizing electrical signals (potentiometry, amperometry, voltammetry) with nanomaterial-enhanced interfaces for real-time, sensitive detection; (2) fluorescent biosensors, leveraging light-emitting probes (FRET, time-resolved fluorescence) for exceptional sensitivity and selectivity; (3) colorimetric biosensors, enabling rapid visual analysis through quantitative/qualitative color changes; (4) spectrometric/chromatographic sensors (MS, IR, HPLC, GC), providing detailed chemical composition and structural data; and (5) optical biosensors (SPR, fluorescence-based), offering real-time, precise monitoring *via* photon-based detection. All platforms share core biosensor attributes, such as biorecognition, specificity, and response, tailored for diverse food safety applications.

The following sections present biological and chemical toxins along with their respective sensing platforms.

## 3. Biological toxins

**Fig. 2:**
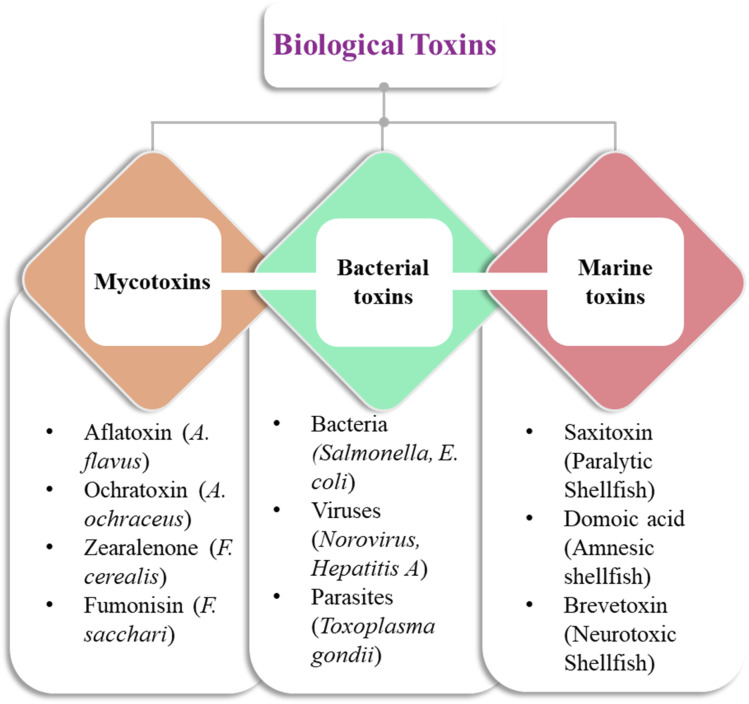
Classification of major biological toxins demanding the implementation of effective prevention and control measures to safeguard food safety.

Biological toxins pose significant risks to both human and animal health.^57^ As discussed *vide supra*, botulinum toxin and aflatoxins are among the most hazardous biological toxins. They are associated with severe health implications, with aflatoxins being linked to serious conditions like liver cancer.^58,59^ Tetrodotoxin found in pufferfish cells is highly perilous, as even minuscule amounts can lead to paralysis and death. Biological toxins disrupt biological cell operations by targeting nerve cells, liver function, or renal function, resulting in moderate to severe health consequences.^60^ Physical health security is fundamentally linked to the analysis of biological toxins, as their prompt identification enables timely interventions that can prevent outbreaks and ensure public safety. Continued research and development efforts have enabled researchers to devise improved methods for detecting and neutralizing these toxins, thereby ensuring the integrity of food supplies. Fig. 2 presents three primary categories of biological food toxins categorized hierarchically by their origins and representative examples. This classification highlights the variety of biological toxins present, emphasizing the need for the development of effective sensing platforms to ensure food safety monitoring.

### 3.1 Mycotoxins

**Fig. 3:**
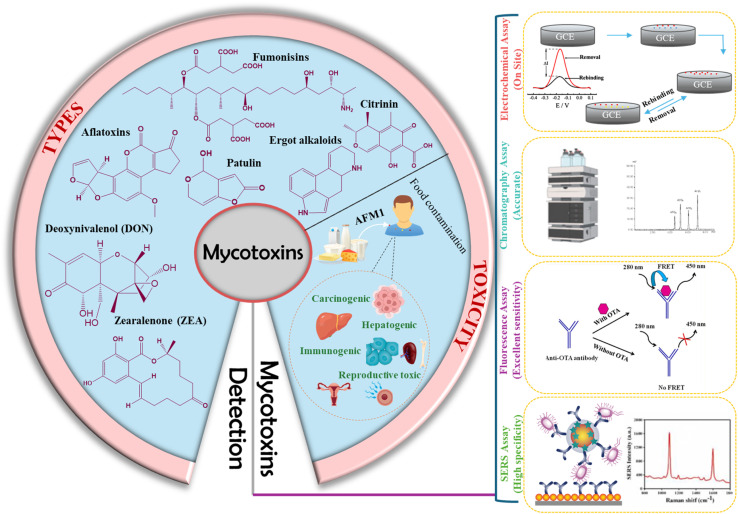
Schematic representation of the structures of mycotoxins, their toxic effects on the human body, and the methods for their detection using sensor technology.

Mycotoxins are harmful substances produced by molds, with “myco” signifying their fungal origin and “toxin” highlighting their toxic characteristics. These are among the most hazardous contaminants in food and animal feed, posing significant health risks to both humans and animals.^55^ The contamination of meat with mycotoxins raises critical concerns regarding food safety. It is believed that such contamination leads to considerable economic losses, in addition to public health and food safety issues, although precise calculations of these impacts remain elusive. The term “mycotoxin” was first introduced in 1960 following the death of 100 000 turkeys in the UK due to feed contaminated with secondary metabolites from Aspergillus flavus.^61^ Mycotoxicosis refers to a health condition resulting from exposure to mycotoxins.^62^ These toxins are categorized based on their harmful effects, including nephrotoxins, immunotoxins, hepatotoxins, and neurotoxins. Additionally, they can be classified into four groups based on their cellular injury mechanisms, including allergens, teratogens, carcinogens, and mutagens. Chemically, mycotoxins are derived from amino acids and polypeptide derivatives. Humans can be exposed to these toxins primarily through the consumption of meat from animals that have ingested contaminated feed, as well as through spices that have been infected during the processing of these meats.^63,64^ Mycotoxins are implicated in several clinical conditions in humans; for instance, fumonisin B1 has been linked to esophageal cancer in the Balkans, while ochratoxin is associated with endemic nephropathy. Currently, over 400 distinct mycotoxins have been identified.^65,66^ A schematic representation illustrating the structures of mycotoxins, their toxic effects on the human body, and their sensor-based detection is provided in Fig. 3.

#### 3.1.1 Aflatoxins

Aflatoxins (AFs) are toxic compounds that can trigger allergic reactions. These substances are derived from coumarin and feature a bifuran group linked to the coumarin core, along with either a pentanone or a lactone ring, which distinguishes between the AFB and AFG types.^67^ Among the 20 identified aflatoxins, AFB1, AFB2, AFG1, and AFG2 are the most significant. The B-type aflatoxins are produced by Aspergillus flavus, while the G-type aflatoxins are generated by *Aspergillus parasiticus*. The biosynthesis of aflatoxins involves at least 25 genes that regulate enzyme production, encompassing 18 enzymatic steps in the process.^68,69^ These toxins are commonly found in various food products and animal feeds, with research indicating that at least one aflatoxin was detected in 37.6% of the grain samples analyzed.^70^ Research indicates that while rice is generally considered low-risk for AF contamination, some varieties of rice have been found to contain AFB1 and other mycotoxins. The ingestion of contaminated food and animal feed, coupled with the presence of residual aflatoxins, poses serious health risks. AFB1, in particular, is a potent carcinogen in humans and is associated with significant health issues.^71^ Acute hepatitis and liver cancer are significantly related, and there are also intermittent acute AF outbreaks that have resulted in fatalities, as highlighted by reports from Kenya concerning lethal aflatoxicosis.^72^ While liver cancer is not very common due to the liver's ability to detoxify aflatoxins, the metabolism of AFB1 results in the production of a reactive intermediate known as AFB1-*exo*-8,9-epoxide, facilitated by cytochrome P450 enzymes.^73^ The detoxification process is aided by glutathione transferases, suggesting that an unidentified protective mechanism against liver cancer may be associated with the detoxification of AFB1-*exo*-8,9-epoxide. Additionally, AF eruptions are primarily found in humid and subtropical regions, with a few instances occurring in temperate climates.^73^ Climate change has altered the geographical distribution of traditional aflatoxins, making Mediterranean regions increasingly vulnerable to aflatoxin contamination. This vulnerability is driven by rising temperatures, increased CO_2_ levels, and changing precipitation patterns, which collectively contribute to a global increase in crop contamination by fungi and aflatoxins.

Before harvest, AFs can significantly impact crops in the field, often in conjunction with drought conditions. The situation becomes even more concerning in storage, where conditions that promote mold growth can exacerbate the problem. Typically, the most critical factors influencing storage quality are the moisture content of the substrate and the relative humidity of the environment. The presence of AFs not only increases mortality rates in agricultural animals but also diminishes the value of grains used as animal feed. Additionally, dairy products can indirectly contain aflatoxins, as cows metabolize AFB1 from contaminated feed into hydroxylated AFM1.^74^ Aflatoxins are toxic and carcinogenic to both humans and animals. Chronic exposure can lead to immunosuppression, cancer, and other serious health issues, while acute exposure may result in fatal outcomes.^75^ AFB1 is particularly harmful, causing liver damage in affected animals, whose susceptibility varies widely. Research on aflatoxin toxicity has primarily focused on experimental animals or those with significant agricultural relevance. The detoxification of aflatoxins occurs in the cytosol and microsomes through a reactive glutathione S-transferase mechanism, which conjugates activated aflatoxins with reduced glutathione.^76^ Differences in glutathione transferase and cytochrome P450 systems may influence the susceptibility to aflatoxins across species. Consequently, due to the variability in aflatoxin susceptibility among test species, concluding its effects on humans has proven challenging; however, instances of acute toxicity in humans remain uncommon.^77^ Notably, a hepatitis outbreak in India in 1974, linked to aflatoxin-contaminated maize, resulted in the deaths of 100 individuals.^78^ Interestingly, a woman who ingested at least 40 mg of pure aflatoxin in a suicide attempt has been reported alive even after more than a decade.^79^ Initial fears that aflatoxin was responsible for Reye's syndrome, a neurological disorder affecting children and adolescents, have been disproven, yet aflatoxin remains a recognized toxin. In individuals with hepatitis B, dietary aflatoxin significantly increases the risk of developing primary hepatocellular carcinoma, and epidemiological studies have consistently associated dietary aflatoxin exposure with a heightened risk of liver cancer.^74^ Evaluating an individual's lifetime exposure to aflatoxin is complex, and findings from such studies have often been inconsistent.

##### 3.1.1.1 Detection of AFs

Aptasensors designed for the electrochemical detection of food toxins facilitate the swift, sensitive, and selective identification of toxins. These sensors utilize aptamers as biorecognition elements, providing high specificity and user-friendliness in complex food matrices. Nodoushan *et al.* developed an electrochemical aptasensor for the detection of AFB1, using a nanocomposite of graphene oxide and gold nanowires. The aptasensor detected AFB_1_ through specific binding between the toxin and its aptamer, resulting in detachment from the electrode and a measurable decrease in the electrochemical signal *via* DPV. This reflects molecular recognition and disruption of electron transfer.^75^ The aptasensor demonstrated a notable change in the peak current of differential pulse voltammetric signals when the aptamer was detached from the electrode surface in the presence of the toxin. It exhibited a linear detection range of 5.0 to 750.0 pM and achieved a limit of detection of 1.4 pM, along with commendable reproducibility. The aptasensor's specificity test showed that it detects AFB_1_ over a blank solution and non-specific compounds. Wang *et al.* also designed a simple electrochemical aptasensor that efficiently detected AFB_1_. A gold electrode was modified with an anti-AFB_1_ aptamer featuring a methylene blue (MB) redox tag at its 3′-end, in the absence of AFB_1_, a complementary DNA (cDNA) strand bound to the aptamer, resulting in the MB moving away from the electrode surface and leading to a reduction in current. AFB_1_ and cDNA then competed for the MB-tagged aptamer, resulting in the formation of a hairpin structure that drew the MB closer to the electrode, thereby increasing the current. This signal-on electrochemical aptasensor is capable of detecting AFB_1_ across a dynamic concentration range from 2 nM to 4 μM. AFB_1_ binds specifically to the methylene blue-labeled aptamer, displacing complementary DNA and inducing a hairpin loop that brings the redox tag close to the electrode, thereby enhancing electron transfer and enabling sensitive electrochemical detection.^80^ Chen *et al.* introduced a self-calibrating single-probe method for ratiometric electrochemical aptasensors, achieving a detection limit for AFB_1_ as low as 0.012 pg mL^−1^ by utilizing an internal self-referencing signal. This aptasensor detects AFB_1_ based on its selective binding to ferrocene-labeled aptamers, which causes the aptamer to detach from the electrode surface, resulting in a measurable decrease in redox current that enables highly sensitive, ratiometric electrochemical detection under varying pH and temperature conditions.^81^

A recent study introduced a novel electrochemical biosensor that utilizes a multifunctional composite material composed of MOF and MXene. This composite combines a molybdenum-based two-dimensional MOF with Ti_3_C_2_ MXene and is applied to a screen-printed electrode (SPE). The article reported the detection of AFB_1_ using an electrochemical biosensor, where anti-AFB_1_ antibodies are covalently immobilized on an MOF/MXene-modified electrode. Antigen–antibody binding hinders electron transfer, as measured by increased charge transfer resistance in impedance spectroscopy. Remarkably, the sensor demonstrated a low LOD of 8 pg mL^−1^, effectively identifying AFB1 concentrations between 0.06 and 50 ng mL^−1^ using electrochemical impedance spectroscopy (EIS). Additionally, it showed strong performance in key quality metrics, including long-term stability, specificity against interfering substances, and both repeatability and reproducibility.^82^ Similarly, Wood *et al.* also detected AFB_1_ electrochemically, using a sensor made of molecularly imprinted polymer (MIP) incorporated into a stainless-steel hypodermic needle. The hypodermic needle sensor, constructed from stainless steel, was developed through a meticulous layer-by-layer film coating process that incorporated AFB_1_-imprinted polyaniline (PANI), cellulose nanocrystals (CNC), and MWCNTs biomimetic receptor films. The PANI@MIP/CNC-CNT hypodermic needle sensor demonstrated a remarkable electrochemical capacitance response (approximately 10 minutes) to AFB_1_, exhibiting a linear range of 0–25 nM and LOD of 3 nM. The sensor exhibited commendable reusability, capable of being utilized up to 7 times while maintaining an RSD of 2.8% in its capacitive response. The high current signal in the MIP-enabled stainless-steel hypodermic needle sensor arises from the synergistic effects of conductive MWCNTs, structurally supportive CNC, and redox-active PANI, MIP, which together facilitate rapid electron transfer, enhance electrochemical capacitance, and enable the selective binding of AFB1, thereby modulating the charge distribution and amplifying the sensing response.^83^ Arzi *et al.* successfully identified AFB1 using modified SPCE/MWCNTs/CS.^80^ In their electrochemical detection method, the signal was generated by the electrons produced during the reduction of TMB(ox) to TMB(red), facilitated by the HRP enzyme, while maintaining a consistent voltage between +0.2 and +0.3 V. The developed electrochemical immunosensor demonstrated a detection limit of 0.3 pg mL^−1^ and a linear working range from 0.0001 to 10 μg L^−1^. The high current signal in this electrochemical immunosensor arises from the synergistic integration of MWCNTs and chitosan on the screen-printed carbon electrode, which enhances conductivity and surface area. At the same time, the enzyme-linked immunoassay mechanism, specifically the HRP-catalyzed redox conversion of TMB, produces a quantifiable electron flow that reflects the competitive binding between AFB_1_ and its conjugate, enabling sensitive and specific detection through efficient electron transfer and catalytic amplification. Notably, this immunosensor was effectively utilized on real peanut samples without any pretreatment, and the analysis of spiked samples indicated a recovery rate of 80–127%.

A study focused on the development of a pencil graphite electrode enhanced with reduced graphene oxide (rGO) and gold nanoparticles (AuNPs) for detecting aflatoxin M_1_ (AFM_1_) in milk samples. The findings revealed that several factors, including aptamer concentration, incubation time for AFM_1_, and duration of exposure, significantly influenced the sensor's effectiveness. The high current signal in this aptasensor originates from the synergistic enhancement of electron transfer by rGO and AuNPs, which increases conductivity and the electroactive surface area. At the same time, the specific binding of AFM_1_ to thiol-modified aptamers immobilized on the electrode surface forms hydrogen bonding and π–π interactions that alter the charge transfer resistance, enabling sensitive and selective detection through EIS. When optimal conditions were met, the EIS technique demonstrated a linear concentration range of 0.5 to 800 ng L^−1^, with a detection limit of 0.3 ng L^−1^.^84^ Additionally, a photoelectrochemical aptasensor was created using sensitized PDA@*f*-MWCNTs/TiO_2_ to detect AFB_1_ in real samples, including groundnuts and milk. The high current signal in this photo-electrochemical aptasensor arises from the synergistic integration of PDA@f-MWCNTs/TiO_2_ nanotube arrays, which enhance visible-light absorption, electron mobility, and charge separation. At the same time, the specific binding of AFB_1_ to NH_2_-functionalized aptamers *via* hydrogen bonding and π–π stacking creates a non-conductive complex that modulates electron transfer and photocurrent intensity through steric hindrance and diffusion limitation of the electron donor. This method exhibited a linear range of 0.005–50 ng mL^−1^ and an impressive detection limit of 1 pg mL^−1^, showcasing remarkable sensitivity and selectivity for AFB_1_ detection.^85^ Another method for the rapid assessment of AFM_1_ in milk and water has been developed, employing a solvent-free approach for signal evaluation. This technique involves the electrodeposition of two layers of PANI onto a GCE, which serves to encapsulate a DNA aptamer that specifically targets AFM_1_. Through the use of EIS and direct current voltammetry, a notable reduction in the intrinsic activity of PANI was observed upon exposure to the AFM_1_ solution. The high current signal in this aptasensor stems from the redox-active emeraldine form of PANI, which facilitates rapid electron transfer due to its conductive polymer matrix; upon binding of AFM_1_ to the NH_2_-functionalized aptamer *via* hydrogen bonding and electrostatic interactions, the analyte induces conformational changes and partial charge shielding, modulating the redox behavior of PANI and enhancing sensitivity through altered electron transfer kinetics. The LOD varied based on the measurement technique, ranging from 1 to 5 ng L^−1^ and 3 to 90 ng L^−1^.^86^

An electrochemical immunosensor for AFB_1_ has been developed using a composite of graphene quantum dots (GQDs) and AuNPs. The carboxyl groups on the GQDs interact with the amino groups of a crosslinker, resulting in the formation of a GQD-AuNP conjugate. Following the application of this composite onto an indium tin oxide (ITO) electrode, AFB_1_ antibodies were introduced. The high current response in this electrochemical immunosensor is attributed to the synergistic enhancement of electron transfer by GQDs and AuNPs, which increase conductivity, surface area, and catalytic activity. At the same time, the specific binding of AFB_1_ to immobilized antibodies involves covalent amide bonding and thiol–gold interactions that modulate charge transfer resistance and facilitate sensitive detection through efficient electron tunneling and bio-nano conjugation. The sensor utilized hexacyanoferrate as the electrochemical probe, demonstrating effective performance across a concentration range of 0.1 to 3.0 ng mL^−1^ of AFB_1_. This biosensor was subsequently employed to analyze maize samples that had been artificially contaminated. Furthermore, it is conceivable that this approach could be adapted for the detection of other mycotoxins by utilizing specific antibodies for each toxin.^87^ Additionally, Qian *et al.* developed a multifunctional aptasensor capable of dual-channel detection of AFB_1_, which exhibited a linear detection range of 5 to 200 ng mL^−1^ and a detection limit of 35 pg mL^−1^. The signal intensification in this aptasensor originates from the catalytic role of AuNPs, which facilitate the deposition of AgNPs and amplify the electrochemical stripping signal through efficient electron transfer at the electrode interface. The sensing mechanism is driven by the specific binding affinity between the thiolated aptamer and AFB_1_, involving non-covalent interactions such as hydrogen bonding and π–π stacking, while the Au–S bond between the aptamer and AuNPs ensures stable probe immobilization and effective signal transduction.^88^

**Fig. 4:**
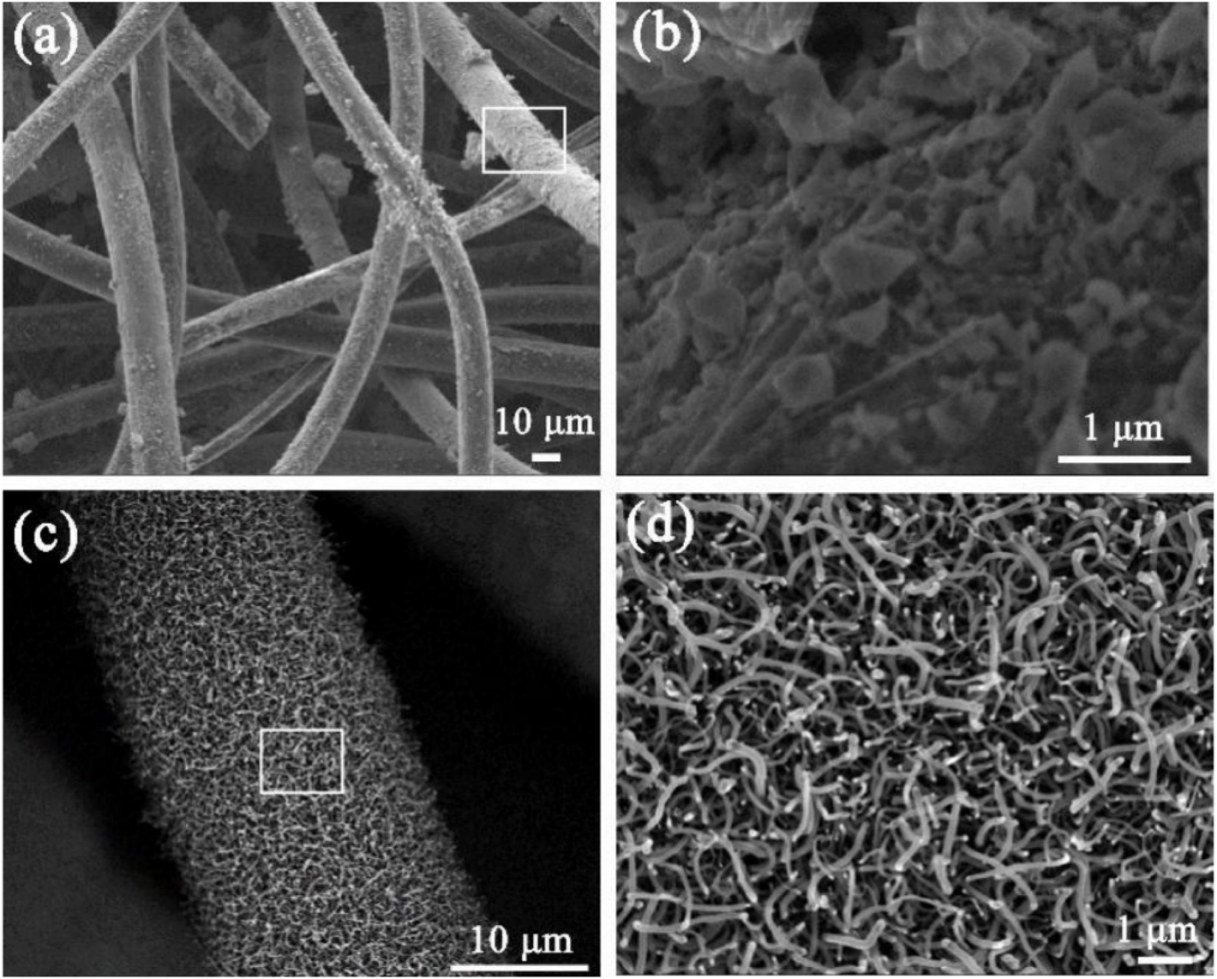
FESEM micrographs of Ni–Cu oxalate-decorated CF (a and b) and SSE (c and d) at different scales. This figure has been adapted from ref. 89 with permission from MDPI, copyright 2022.

A multi-scaled electrochemical biosensor was developed by integrating an aptamer, horseradish peroxidase, and carboxylated polystyrene nanospheres onto a carbon nanofiber/carbon felt platform. The three-dimensional porous structure of the carbon nanofiber and carbon felt, as shown in the FESEM micrographs in Fig. 4, provides an excellent substrate due to its high conductivity and efficient transport of reactants. Horseradish peroxidase and carboxylated polystyrene nanospheres served as signal amplification probes, facilitating the decomposition of H_2_O_2_ and improving electrochemical responses. The biosensor employed DPV to detect AFB1 in soy sauce and wine samples, achieving recovery rates ranging from 87.53% to 106.71%, with a limit of detection of 0.016 pg mL^−1^.^89^ Wang *et al.* designed a competitive electrochemical aptamer-based technique to detect AFB_1_. A gold electrode was coated with the cDNA strand of an anti-AFB_1_ aptamer and subjected to a sample solution containing an aptamer labeled with MB. The increase in signal intensity in this competitive electrochemical aptamer-based sensor arises from the proximity of MB to the gold electrode when the MB-labeled aptamer hybridizes with the immobilized cDNA, enabling efficient electron transfer. In the presence of AFB_1_, the aptamer preferentially binds AFB_1_ through non-covalent interactions, such as hydrogen bonding and π–π stacking, thereby preventing hybridization with cDNA and reducing the proximity of MB, which lowers the current signal and enables selective detection. Under carefully calibrated testing settings, the sensor's dynamic detection range was 2–500 nM, and it identified 2 nM AFB_1_.^90^

**Fig. 5:**
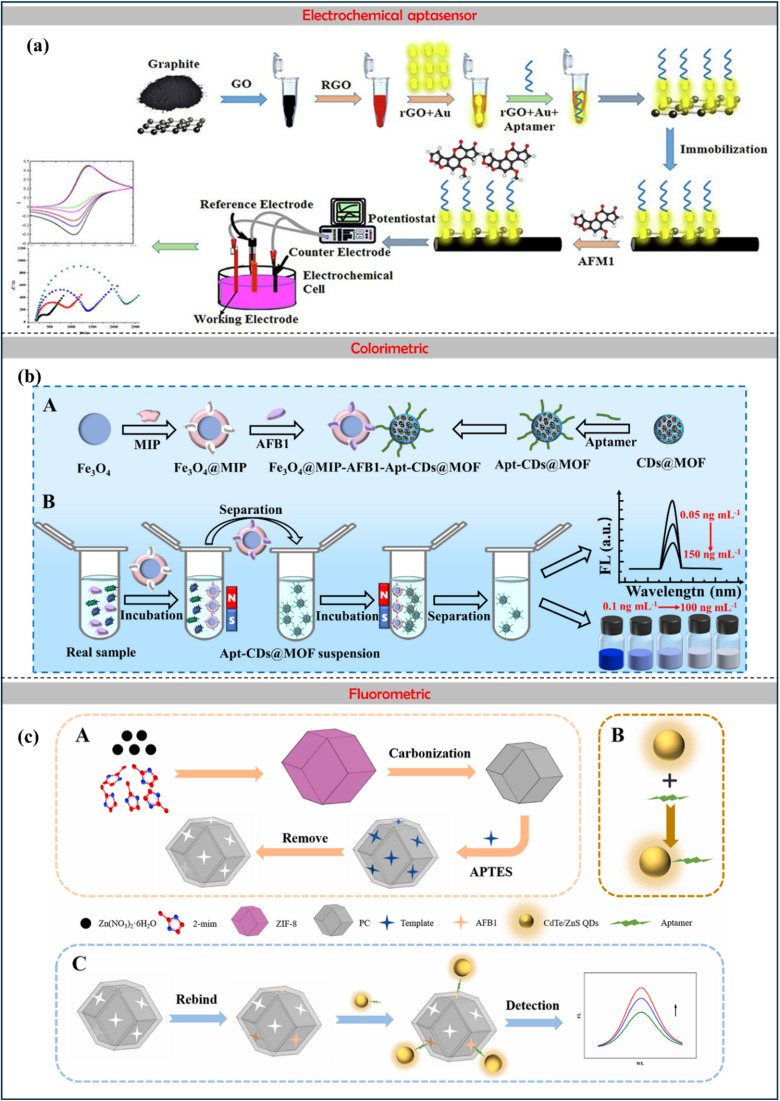
Electrochemical aptasensor (a) monitoring of Aflatoxin M1 in milk using a novel electrochemical aptasensor based on reduced graphene oxide and gold nanoparticles, reproduced from ref. 84 with permission from Elsevier, copyright 2022. Colorimetric: (b) fluorescence/colorimetric sensor based on aptamers-molecular imprinted polymers synergistic recognition for ultrasensitive and interference-free detection of aflatoxin B_1_, reproduced from ref. 91 with permission from Elsevier, copyright 2025. Fluorometric: (c) schematic illustration of Preparation of the MIP/PC imprint layer (A); preparation of fluorescent CdTe/ZnS-Apt probe (B); and detection of AFB_1_ by the sandwich biosensor (C), reproduced from ref. 92 with permission from Elsevier, copyright 2023.

**Fig. 6:**
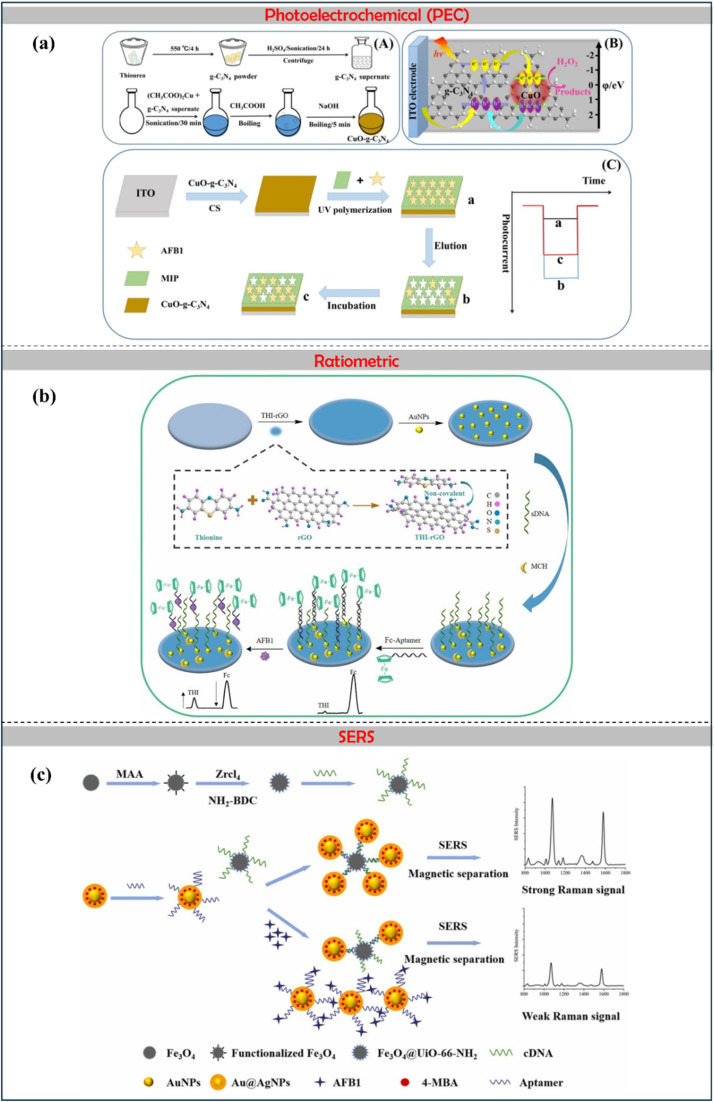
Photoelectrochemical: (a) construction of CuO-g-C_3_N_4_ heterojunction-based MIP-PEC sensors for ultrasensitive detection of aflatoxin B_1_, reproduced from ref. 93 with permission from Elsevier, copyright 2021. Ratiometric: (b) a sensitivity programmable ratiometric electrochemical aptasensor was fabricated based on thionine-graphene nanocomposite for the detection of AFB_1_ in peanut, reproduced from ref. 94 with permission from Elsevier, copyright 2020. SERS: (c) development of an ultrasensitive SERS aptasensor for the determination of AFB_1_ by modifying magnetic beads with UiO-66-NH_2_ for enhanced signal probe capturing, reproduced from ref. 95 with permission from Elsevier, copyright 2023.

The biosensing strategies illustrated in Fig. 5 showcase advanced techniques for detecting aflatoxins in food, emphasizing high sensitivity, selectivity, and multiplexing capabilities. The electrochemical aptasensor (Fig. 5a) utilizes nanomaterials, including rGO, AuNPs, and MOFs, to enhance signal transduction for the detection of AFM_1_ in milk. Optical methods, such as colorimetric (Fig. 5b) and fluorometric (Fig. 5c) biosensors, integrate aptamers, MIPs, and quantum dots (CdTe/ZnS) for interference-free and ultrasensitive AFB_1_ detection. Further, Fig. 6 also illustrates aflatoxin detection, where Photoelectrochemical (PEC) sensors (Fig. 6a) exploit CuO-g-C_3_N_4_ heterojunctions to boost photocurrent responses in molecularly imprinted polymer-based AFB_1_ sensing, while ratiometric sensors (Fig. 6b) leverage thionine-graphene nanocomposites to improve accuracy in AFB_1_ detection. Additionally, surface-enhanced Raman spectroscopy (SERS) aptasensors (Fig. 6c) enhance sensitivity through the use of UiO-66-NH_2_-modified magnetic beads, enabling the ultra-trace quantification of AFB_1_. Collectively, these approaches highlight the integration of nanomaterials, biorecognition elements, and innovative transduction mechanisms, paving the way for next-generation biosensors in food safety monitoring.

An electrochemical aptasensor was developed to simultaneously detect ochratoxin A (OTA) and AFB_1_. This sensor incorporated haemin and ferrocene within the HKUST-1 MOF, which consists of copper nodes and trimesic acid. The framework was further enhanced by integrating complementary DNA sequences specific to AFB_1_ and OTA aptamers, resulting in the formation of Hemin@HKUST-1 and Ferrocene@HKUST-1. The improved current response in this duplexed electrochemical aptasensor arises from the synergistic conductivity of carbon nanodots decorated with AuNPs, which enhances electron transfer between the transducer and redox-labeled aptamer complexes. Upon the specific binding of aflatoxin B1 and ochratoxin A to their respective aptamers, non-covalent interactions, such as hydrogen bonding and π–π stacking, displace the ferrocene@HKUST-1 and hemin@HKUST-1 bioconjugates, modulating the redox signal and enabling highly sensitive detection through DPV. DPV was used to measure the current response to the electroactive labels of ferrocene and hemin in the bioconjugates at two potentials simultaneously, in the absence of the two mycotoxins. The detection limits for OTA and AFB_1_ mycotoxins were 4.3 and 5.2 × 10^−3^ ng mL^−1^, respectively, and ranged from 1.0 × 10^−2^ to 100 ng mL^−1^.^96^ Selvolini *et al.* developed an electrochemical enzyme-linked oligonucleotide array for the rapid detection of AFB_1_ by employing a multi-detection assay within a competitive framework. The poly(aniline-anthranilic acid) copolymer (PANI-PAA) was first deposited onto graphite SPC using cyclic voltammetry (CV). AFB1-BSA was covalently attached to the PANI-PAA copolymer for immobilization. The resolution was implemented on modified SPCs after the affinity interaction between AFB_1_ and apt-BIO. Streptavidin and alkaline phosphatase conjugates were attached to biotinylated complex sensors. The electroactive product derived from 1-naphthyl phosphate, the enzymatic substrate, was identified through DPV. Covalent bonding between the carboxyl groups of the copolymer and the amino groups of the AFB_1_–BSA conjugate ensures stable immobilization, while specific aptamer–AFB_1_ interactions, primarily hydrogen bonding and π–π stacking, trigger competitive displacement, modulating the enzymatic redox signal for precise detection. Within the range of 0.1 to 10 ng mL^−1^, a dose–response relationship was observed with a detection threshold of 0.086 ng mL^−1^.^97^

Lin *et al.* developed an electrochemical aptasensor for AFB_1_ detection by employing layer-by-layer self-assembly. Positively charged poly (diallyl dimethylammonium chloride) nanosheets and negatively charged carboxylated polystyrene nanospheres were arranged in alternating layers on the electrode. Multi-layered sandwich electrodes featured numerous electrochemically active regions and exhibited good conductivity, with amide bonds formed between carboxyl-functionalized polystyrene nanospheres and AFB_1_ aptamers. To increase selectivity, ethanolamine and bovine serum albumin were applied to the electrode. Nyquist plots showed that the sample AFB_1_ concentration is substantially associated with electrode charge transfer resistance. Among the most successful mycotoxin detection technologies, the improved electrochemical aptasensor has a detection limit of 0.002 ng mL^−1^ and stability over 30 days. The electrochemical aptasensor recovered 94.5–103.3% AFB_1_ from soy sauce and oil.^98^ Using aptamers and AuNPs on a conductive boron-doped diamond electrode, Feng *et al.* constructed an electrochemical aptasensor to detect AFB_1_. The high sensitivity in this boron-doped diamond (BDD)-based electrochemical aptasensor results from the synergistic conductivity of the BDD electrode and AuNPs, which enhance electron transfer and provide abundant active sites for aptamer immobilization *via* strong Au–S covalent bonding; upon specific binding of AFB_1_ to the aptamer through hydrogen bonding and π–π stacking, the resulting spatial conformation hinders redox probe access, modulating charge transfer resistance and enabling precise, concentration-dependent impedance shifts. With a linear relationship range of 1.0 × 10^−13^ to 1.0 × 10^−8^ mol L^−1^, the EIS analysis revealed that the aptasensor had a low detection limit of 5.5 × 10^−14^ mol L^−1^.^99^ Additionally, Meng *et al.* demonstrated the synthesis of a platinum-coordinated titanium-based porphyrin MOF, specifically designed for the development of an aptamer sensor targeting AFB_1_. The high specific surface area of the MOF, coupled with the excellent electrochemical properties of platinum, significantly enhanced the sensor's performance. The modified AFB1 aptamer sensor exhibited a linear detection range of 0.1 to 75 μg L^−1^ and a detection limit of 31 ng L^−1^.^100^

Another study introduced a pistachio AF sensor with a confirmatory cross-validation. This sensor utilized a competitive assay integrated with a screen-printed carbon electrode (SPCE) and was assessed through electrochemical methods. The intense signal and improved sensitivity in this electrochemical immunosensor are attributed to the efficient electron transfer facilitated by the SPCE and the catalytic reduction of TMB by HRP-labeled antibodies. In contrast, the specific binding between aflatoxins and monoclonal antibodies, mediated through non-covalent interactions such as hydrogen bonding and hydrophobic forces, modulates the competitive immunoassay response, resulting in a quantifiable current proportional to the analyte concentration. It demonstrated a linear detection range of 0.01 to 2 μg L^−1^ and exhibited excellent repeatability, with RSD of 2%. The detection limits were found to be 0.017 μg L^−1^ in phosphate-buffered saline and 0.066 μg kg^−1^ within the pistachio matrix, both significantly lower than the European regulatory threshold for total aflatoxins in pistachios.^101^ Additionally, Gevared and coworkers reported a facile synthesis of AuNP-GQD nanocomposite, which was employed as an electrode modifier for the voltammetric measurement of AFB_1_. The oxidation of AFB_1_ using this device resulted in an enhanced electrocatalytic effect, improving the voltammetric response and shifting the peak potential to more positive values. EIS revealed that the modified electrodes exhibited lower charge transfer resistance values. Under optimal conditions, a linear analytical curve was established, yielding detection and quantification limits of 0.47 and 1.5 nmol L^−1^, respectively.^102^Table 1 illustrates the various methods used to detect aflatoxins and their corresponding detection limits.

**Table 1:** Aflatoxin detection in a variety of food samples using different sensing platforms

S. No.	Method	Analyte	Electrode modifier	Linear range	LOD	Sample	Ref.
1	CV and EIS	AFB_1_	AP/Ti-MOFs-Pt/GCE	0.1–75 μg L^−1^	31 ng L^−1^	—	100
2	DPV	AFB_1_	—	0.5 nM–4 μM	0.07 nM	Cow milk	103
3	DPV	AFB_1_	GCE/RGO/MoS_2_/PANI@AuNPs/Apt/MCH	0.01–1.0 fg mL^−1^	0.002 fg mL^−1^	Wine	104
4	DPV	AFB_1_	APT/TDNs/3DOM MoS_2_-AuNPs	0.1 fg mL^−1^–0.1 μg mL^−1^	0.01 fg mL^−1^	Rice, wheat powder	105
5	DPV	AFB_1_	AFB_1_-BSA/PANI-PAA/GSPEs	0.1–10 ng mL^−1^	0.086 ng mL^−1^	Maize flour	97
6	DPV	AFB_2_	SPCE/PAM/PA/PDA/Apt	0.0001–100 ng mL^−1^	0.10 pg mL^−1^	Corn	106
7	DPV	AFM_1_	Apt-modified SPGE	2–600 ng L^−1^	0.9 ng L^−1^	Milk and serum	107
8	EIS	AFB_1_	Apt-Fe_3_O_4_@Au/SPCE	0.020–50 ng mL^−1^	15 pg mL^−1^	Peanuts	108
9	EIS	AFM_1_	—	5–120 ng L^−1^	0.5 ng L^−1^	Milk	109
10	EIS	AFM_1_	Anti-AFM_1_/Fc/SiNPs-PpPD/SPCE	10–500 fM	4.53 fM	Milk	110
11	EIS and DPV	AFB_1_	Fc-cDNA/pβ-CD/AuNPs/GC	0.0001–10 ng mL^−1^ (EIS) 0.001–10 ng mL^−1^ (DPV)	0.049 pg mL^−1^ (EIS) 0.511 pg mL^−1^ 0.511 pg mL^−1^ (DPV)	Peanut oil/94.5–106.7%	111
12	SWV	AFB_1_	MBs/CdTe-cDNA1/Apt-SiO_2_@PbS	0.005–50 ng mL^−1^	4.5 pg mL^−1^	Peanuts	112
13	CV	AFB_1_	BSA/anti-AFB_1_/GQD-AuNPs/ITO	0.1–3.0 ng mL^−1^	0.008 ng mL^−1^	Maize	87
14	CV	AFB_1_	GO-PB-PTCNH_2_-AuNPs	0.01–100 ng mL^−1^	3.3 pg mL^−1^	Wheat	113
15	I-T	AFB_1_		1–20 ng mL^−1^	0.13 ng mL^−1^	—	114
16	ECL	AFM_1_	Apt-GMNP-GO-L-AgNPs	5–150 ng mL^−1^	0.01 ng mL^−1^	Milk	115
17	ECL	AFM_1_	H1/Au NPs/PTCA/GCE	0.0004–400 ng mL^−1^	0.09 pg mL^−1^	Milk	116
18	ECL	AFB_1_	Au-PEI@SiO_2_-AFB_1_-BSA	0.01–100 ng mL^−1^	0.0039 ng mL^−1^	Milk	117
19	ECL and EC	AFB_1_	—	0.0001–100 ng mL^−1^ (ECL)	0.058 pg mL^−1^ (ECL)	Peanut	118
				0.0001–100 ng mL^−1^ (EC)	0.018 pg mL^−1^ (EC)		
20	PEC	AFB_1_	ITO/Zn–SnS_2_/N-GQDs/(EDC/NHS)/aptamer/DA	0.01–20 ng mL^−1^	3 ng mL^−1^	Milk	119
21	PEC	AFB_1_	AuNPs-labeled DNA|aptamer|Ce–TiO_2_@MoSe_2_|ITO	0.03–200 ng mL^−1^	10 pg mL^−1^	Peanut	120
22	PEC	AFB_1_	Au/AchE/TiO_2_NTs	1–6 nM	0.33 nM	—	121
23	PEC	AFB_1_	AFB_1_/Aptamer/erGO/P5Fin/Au/ITO	0.01–100 ng mL^−1^	2 pg mL^−1^	Peanuts, wheat	122
24	EIS	AFB_1_	Ab-AFB_1_/BSA/AuNPs-PABA/RGO/GCE	0.01–1 ng mL^−1^	0.001 ng mL^−1^	Vegetable oil/unspiked	123
25	EIS	AFB_1_	Ab-AFB_1_/BSA/AuNPs–COOH–GO/GCE	0.05–25 ng mL^−1^	0.05 ng mL^−1^	Rice/99–101%	124
26	CV	AFB_1_	SA/Biotin Ab-AFB_1_/SPE	0.00005–5 ng mL^−1^	50 fg mL^−1^	Rice milk/unspiked	125
27	DPV	AFB_1_	BSA/Ab-AFB_1_/Mn_2_O_3_/ITO	1 pg mL^−1^–10 μg mL^−1^	0.54 pg mL^−1^	Sweet corn/96–99%	126
28	DPV (indirect HRP)	AFB_1_	Ab-AFB_1_/MWCNTs/Chi/SPCE	0.0001–10 ng mL^−1^	0.3 pg mL^−1^	Peanut/unspiked	127
29	CA (direct HRP)	AFM_1_	Ab-AFM_1_/SWCNTs/PET	0.01–1 ng mL^−1^	0.02 ng mL^−1^	Milk/unispiked	128
30	PEC	AFB_1_	CsPbBr_3_/TiO_2_/FTO	0.01–15 ng mL^−1^	2.8 pg mL^−1^	Peanut/90–104% corn/95–109%	129
31	PEC	AFB_1_	CdS/CoOOH/FTO	0.01–10 ng mL^−1^	2.6 pg mL^−1^	Peanut/unspiked	130
32	DPV	AFB_1_	Ab-AFB_1_/Fc/MWCNTs/Chi/SPCE	0.001–20000 ng mL^−1^	0.159 pg mL^−1^	Rice/94%	131
						Peanut/102%	
						Corn/96%	
33	EIS	AFM_1_	Apt/Fc/SiNPs/PpPD/SPCE	3.3–165 fg mL^−1^	1.48 fg mL^−1^	Milk/unspiked	110
34	EIS	AFM_1_	BSA/Apt/PtNPs/MIL-101(Fe)GCE	0.01–80.0 ng mL^−1^	0.002 ng mL^−1^	Milk/93–108%	132
35	EIS	AFB_1_	MCH/MCH-Apt/AuNPs/BDD	0.03–3122.8 pg mL^−1^	0.017 pg mL^−1^	Peanut/96–109%	99
36	EIS	AFB_1_	Apt/BSA/PS-COOH/PDDA-GN/GCE	0.001–0.1 ng mL^−1^	0.002 ng mL^−1^	Oil/98–103%	98
						Soy sauce/95–102%	
37	DPV (HRP)	AFB_1_	DNA-AuNPs-HRP/Apt/cDNA/AuNPs/GCE	0.001–200 ng mL^−1^	0.33 pg mL^−1^	Peanut & corn	133
38	LSV (Au@Ag)	AFB_1_	SPCE–CS–ccDNA/cDNA-AuNPs	0.05–100 ng mL^−1^	0.43 pg mL^−1^	Corn/93–98%	88
39	ACV	AFB_1_	Fc-Apt/cDNA/AuNPs/THI-rGO/GCE	0.05–20 ng mL^−1^	0.016 ng mL^−1^	Peanut/87–112%	94
40	DPV (ALP)	AFB_1_	SA-ALP/Biotin-Apt/AFB_1_-BSA/AuNPs-PANI/GSPE	0.1–10 ng mL^−1^	0.086 ng mL^−1^	Corn & maize flour	97
41	EIS or DPV	AFB_1_	Apt/Cu_2_O-CQDs/GCE	3 ag mL^−1^–1.9 μg mL^−1^	0.9 ag mL^−1^	Wheat flour/97–104%	134
42	EIS	AFB_1_	Fc-apt/THI-rGO/GCE	0.01–100 ng mL^−1^	0.01 ng mL^−1^	Peanut/94–116%	135
43	LSV	AFB_1_	AuNPs-GQDs/SPE	0.03–15.6 ng mL^−1^	0.15 ng mL^−1^	Malted barley/76–190	102
44	EIS	AFB_2_	ZnO-NPs/CS/PPy/AFB_2_	0.1–1000 fg mL^−1^	0.2 Fg mL^−1^	Milk	136
	DPV		ZnO-NPs/CS/AFB_2_		0.6 fg mL^−1^		
45	PEC	AFB_1_	CuO-g-C_3_N_4_/MIP	0.01 ng mL^−1^–1 μg mL^−1^	6.8 pg mL^−1^	Maize/99–106%	93
46	CV and EIS	AFB_1_	AP/Ti-MOFs-Pt/GCE	0.1–75 μg mL^−1^	31 ng mL^−1^	Corn flour/96.05%	100
						Rice/104.45%	
47	DPV	AFB_1_	BSA/Anti-AFB_1_/Mn_2_O_3_/ITO	1 pg mL^−1^–10 μg mL^−1^	0.54 pg mL^−1^	Sweet corn/98.6%	126
48		AFB_1_	CCM@ZIF-8/PDA/IgG	0.5 pg mL^−1^–10 ng mL^−1^	0.11 pg mL^−1^	Rice/86.65%	137
						Wheat/105.20%	
49	CV and EIS	AFM_1_	AuNPs/rGO/PGE/aptamer	0.5–800 ng mL^−1^	0.3 ng mL^−1^	Milk	84
50	DPV	AFB_1_	Aptamer/MCH/cDNA/PDA/MXene/MWCNTs/NiCo_2_O_4_/SGPGE	2.5–200 ng mL^−1^	1.89 ng mL^−1^	Maize flour/92.2%	138
						Maize residue/109.8%	
51	EIS	AFB_1_	Anti-AFB_1_/NH_2_–Mo-MOF/Ti_3_C_2_ modified SPE	0.06–50 ng mL^−1^	8 pg mL^−1^	Pistachio	82
52	DPV	AFB_1_	PS-BSA-Apt-Au/SSE	0.1–10 pg mL^−1^	0.016 pg mL^−1^	Wine/87.5%	89
						Soy sauce/106.7%	
53	EC	AFB_1_	Fc-aDNA/Fc-Apt/MCH/sDNA/AuNPs/GCE	0.1–10000 pg mL^−1^	0.012 pg mL^−1^	Corn/96.7–102.9%	81
54	PEC	AFB_1_	PDA@f-MWCNTs/TiO_2_-NTs	0.005–50 ng mL^−1^	1 pg mL^−1^	Groundnut and milk	85
55	EC	AFB_1_	Apts/AuNPs-CNDs/GCE	0.01–100 ng mL^−1^	0.0052 ng mL^−1^	Corn flour/98.4–101.3%	96
56	CV	AFB_1_	AuNPs/Zn/Ni-ZIF-8-800@graphene	0.18–100 ng mL^−1^	0.18 ng mL^−1^	Peanut oil	139
57	LSPR	AFB_1_	AuNPs/Nb_2_CT_*x*_ MXene	0–1000 nM	26.41 nM	—	140
58	ELISA	AFB_1_	CdTe/ZnS-Apt/MIP/PC	0.01–20 ng mL^−1^	4.0 pg mL^−1^	Edible oil/91.9–102.6%	92
59	FRET	AFM_1_	AFM_1_APT/AFB_1_APT/C1/C2/C3	0.01–200 ng mL^−1^	6.24 pg mL^−1^	(Peanut/98–102.3% pure milk/98.4–105%)	141
		AFB_1_		0.01–150 ng mL^−1^	9.0 pg mL^−1^	(peanut/99.4–103% pure milk/98.9–102.5%)	

#### 3.1.2 Ergot alkaloids

Mycotoxins known as ergot alkaloids (EAs), primarily produced by the Claviceps fungus, have been recognized as harmful since the 5th century AD. The term “ergot” is derived from the old French word “argot,” which means “cock's spur.” Ergotism represents the earliest documented mycotoxicosis, leading to illness in both humans and animals through the ingestion of food or feed contaminated with these alkaloids.^142,143^ The earliest recorded epidemic of ergotism, which resulted in the death of more than 10 000 individuals, occurred in France between 944 and 945 AD.^79^ Ergotism was widespread in Eastern Russia, Germany, and France between the 9th and 14th centuries.^144,145^ Three categories of entheogenic alkaloids—clavines, lysergic acid amides, and ergopeptines—are distinguished by their structural resemblance. Ergotamine and ergosine, the two most well-known ergot alkaloids produced by Claviceps species, utilize tetracyclic ergoline rings and are stable alkaloids that are relatively unaffected by heat. Conversely, heating can diminish the levels of ergocornine, ergocristine, ergokryptine, and ergometrine.^146^ Alkaloids are categorized into ergopeptines and ergopeptinines, each possessing distinct biological and physicochemical features. The levorotatory isomers of EAs are referred to as ergopeptins, while the dextrorotatory isomers are known as ergopeptinines, with the highest and lowest biological activity found in ergopeptines. The conversion of ergopeptine is accelerated in aqueous acidic or alkaline solutions. Various species, including Claviceps purpurea, Claviceps fisiformis, Claviceps africana, Epichloë, and Neotyphodium, produce EAs, along with *Aspergillus*, *Penicillium*, and *Blansia*, all of which can significantly affect cereal production.^147,148^

Sorghum bicolor agriculture has experienced significant financial setbacks due to the adverse effects of C. africana, resulting in declines in seed quality and overall production. Additionally, challenges in harvesting and threshing processes, coupled with restrictions on international seed trade, have further exacerbated these losses.^149^ Furthermore, in developing nations, the poisoning of individuals who ingest food infected with sclerotia (the dormant structures of the fungus) indicates that ergotism has not been eradicated and continues to pose a significant threat. In Ethiopia, 47 people died of ergotism in the spring of 1978 after eating barley contaminated with C. purpurea sclerotia.^150^ Biogenic amine receptors can be bound by endogenous amines, which modify neurotransmission, and their biological effects can be correlated with those of pharmaceuticals due to their structural resemblance to neurotransmitters such as noradrenaline, dopamine, adrenaline, and serotonin.^151^ Notwithstanding the laborious extraction of ergot bodies, the grains still contained ergot alkaloids. When grasses or cereals are harvested, Sclerotia can contaminate the feed and cereal-based food with EAs. Sclerotia are fragmented and blended with flour during cereal milling, rendering them challenging to detect. C. purpurea parasitises a minimum of 400 Poaceae species, with the most impacted being cereals such as barley, millet, oats, wheat, rye, and triticale, as well as various weedy grasses.^152^

The primary health concerns associated with EAs in humans and animals are mycotoxins that lead to convulsive and gangrenous ergotism. This condition, one of the earliest recognized diseases linked to mycotoxins, can manifest through symptoms such as hallucinations, convulsions, agalactia, burning sensations, vasoconstriction, and even the loss of limbs due to gangrene following the consumption of contaminated food. Nausea, endocrine dysfunction, vomiting, weakness, numbness, cardiovascular issues, and death are also noted. Ethiopians contracted gangrenous ergotism in 2001 after eating infected barley. Pigs, cattle, sheep, rabbits, and poultry all exhibited symptoms following an EA infection, which was costly for breeders and the meat industry.^153^ Animals that consume grains affected with EA may develop a variety of diseases that affect any part of their bodies.^79^ Detection of EAs using various methods is discussed in the subsequent section.

##### 3.1.2.1 Detection of EAs

Huybrechts *et al.* developed a UHPLC-MS/MS method to comply with the new EU regulation that restricts ergot alkaloids in dry cereal-based infant foods to 20 ng g^−1^. This technique enabled the quantification of six specific ergot alkaloids, including ergocornine, ergocristine, ergometrine, ergosine, ergotamine, and α-ergocryptine, with a detection limit of 0.5 ng g^−1^. The method employs enhanced QuEChERS extraction followed by UHPLC-MS/MS analysis, demonstrating high sensitivity, accuracy, and precision. In a study involving 49 samples from Belgium, 26 samples showed no presence of EAs, while 23 contained at least one type of alkaloid, with two samples each having 12. Notably, ergometrine was the most frequently detected alkaloid, appearing in 16 of the 49 samples.^154^ Another study utilizing MIP technology focused on the electro-polymerization of dopamine monomers on a GCE, guided by a methylergonovine maleate (MM) template. The improved responsiveness in this electrochemical sensor is attributed to the electrocatalytic activity of the polydopamine (PDA)-based MIP film, which enhances electron transfer between the GCE and MM by creating selective recognition cavities. These cavities facilitate specific rebinding through hydrogen bonding and electrostatic interactions between the functional groups of dopamine and MM, accelerating charge transfer and amplifying the electrochemical response. This sensor achieved a LOD of 0.016 nM and exhibited a linear detection range from 0.05 to 300 nM. It was successfully employed to detect MM in Methergin tablets and blood serum samples, demonstrating effective performance in practical applications with recovery rates ranging from 98.8% to 102.3%.^155^

Tajik *et al.* employed SPE in conjunction with a Fe_2_MoO_6_ magnetic nanocomposite to create a sensing platform for the detection of cabergoline. The Fe_2_MoO_6_ magnetic nanocomposite enhanced the efficiency of the recognition process. The modified electrode demonstrated significantly higher oxidation peak currents for cabergoline compared to traditional screen-printed electrodes. The improved performance of the Fe_2_MoO_6_-modified screen-printed electrode arise from the nanocomposite's high surface area and superior electrical conductivity, which facilitate rapid electron transfer and efficient electrocatalytic oxidation of cabergoline. The sensing mechanism is primarily diffusion-controlled, and the interaction between cabergoline and the modifier likely involves hydrogen bonding and electrostatic interactions between the analyte's amide and amine groups and the oxide surface, enhancing selectivity and signal amplification. A linear analytical response was established for cabergoline concentrations ranging from 0.08 to 300.0 μM, with a detection limit of 0.02 μM.^152^ Additionally, another study highlighted the preliminary detection of cabergoline in aqueous solutions using a nanostructure-modified carbon paste electrode integrated with an ionic liquid. This approach employed a SiO_2_@Fe_3_O_4_/GO nanocomposite, designed explicitly as a bromide binder for 1-methyl-3-butylimidazolium. The signal intensification of this sensor is attributed to the synergistic conductivity of the SiO_2_@Fe_3_O_4_/GO nanocomposite and the ionic liquid binder, which enhances electron transfer and expands the electroactive surface area. Meanwhile, cabergoline undergoes electrocatalytic oxidation *via* diffusion-controlled mechanisms. The interaction between cabergoline and the modifier likely involves hydrogen bonding and electrostatic interactions between its amide and amine groups and the carboxyl-functionalized GO surface, facilitating selective recognition and amplified electrochemical response. The SiO_2_@Fe_3_O_4_/GO/CPILE exhibited a cabergoline oxidation peak at 500 mV, which is 200 mV lower than that observed on the conventional carbon paste electrode under similar conditions. Furthermore, the peak current at the SiO_2_@Fe_3_O_4_/GO/CPILE surface was found to be three times greater than that of the CPE. The detection limit was determined to be 0.01 μM, with a linear response range established between 0.07 and 500.0 μM.^156^

A unique UHPLC-FLD method was used to quantify the six main EAs and their associated epimers in another investigation. The sample preparation involved solid–liquid extraction using acetonitrile and freeze-out cleanup. The high sensitivity of this UHPLC-FLD-based analytical method for ergot alkaloids is attributed to the efficient separation and detection capabilities of the ultra-high-performance liquid chromatography system, coupled with fluorescence detection, which enhances signal intensity through the selective excitation and emission of alkaloid molecules. While no direct electrochemical sensing is involved, the analyte–modifier interaction during sample preparation may include hydrogen bonding and π–π stacking between ergot alkaloid functional groups and matrix components, facilitating selective extraction and quantification. Before analysis, materials were sieved (1.9 × 20 mm) to remove alkaloid-containing sclerotia. However, 23 samples had ergot alkaloid concentrations over the detection limit; the range for the six alkaloids was 0.3 to 2530.1 μg kg^−1^.^157^ Twelve EAs were carefully measured in 228 swine feed samples. Analytes were extracted using QuEChERS and Z-Sep + for cleanup. This method reduced the matrix impact, resulting in quantification limits of 2.1 to 21.7 μg kg^−1^. After quantification using UHPLC–MS/MS, 29 samples (12.7%) were found to be contaminated with EA. Interestingly, 65% of infected samples had several alkaloids. Only 6 of the 12 targeted EAs exceeded quantitative limits. The most prevalent alkaloid, ergometrine, had an EA value of 145.3 μg kg^−1^, whereas ergosinine had an EA level of 5.9 μg kg^−1^. The range of the total EA concentration was 5.9 to 158.7 μg kg^−1^.^158^Table 2 depicts the detection of various EAs.

**Table 2:** Detection of EAs using different methods

Sr No.	Method	Analyte	Electrode modifier	Linear range	LOD	Sample	Ref.
1	Spectrofluorimetry	Bromocriptine mesylate	—	50–600 ng mL^−1^	14.57 ng mL^−1^	—	159
2	Spectrofluorimetry TLC	Cabergoline	—	50–450 ng mL^−1^	14.4 ng mL^−1^	—	160
				100–1500 ng band^−1^	25.4 ng band^−1^		
3	HPTLC	Cabergoline and its degradation products	—	0.05–2.0 ng band^−1^	—	Methacarbamol/99.8%	161
	HPLC			1–40 μg mL^−1^		Fluphenazine/99.7%	
4	CV	Methylergonovine maleate	—	0.05–300 nM	0.016 nM	Methergine tablet and blood serum	155
	DPV						
	EIS						
5	DPV	Levodopa	2D Ni-MOF NSs/ILCPE	0.09–365.0 μM	0.03 μM	Cabergoline tablet	162
						Urine	
6	CV	Cabergoline and levodopa	GQDs/2CBF/IL/CPE	0.07–500 μM	0.02 μM	Urine	163
	DPV						
						Serum	
						Cabergoline tablets	
7	SWV	Lysergic acid diethylamide (LSD)	—	5.0–100 μmol L^−1^	0.5 μmol L^−1^	Seized blotter paper samples	164
8	Amperometry	Ergometrine	—	6 nM–1.2 μM	3 nM	Rye flour	165
9	ELISA	Ergometrine	—	—	29.4 μg mL^−1^	Flour and milk	166
10	HPLC-FLD	Ergotamine	—	—	0.05 μg kg^−1^	Rye-wheat bread and rye-wheat flour	167
		Ergocristine			0.15 μg kg^−1^		
11	DPV	Cabergoline	La_2_O_3_/Co_3_O_4_/SPE	0.1–100 μM	0.04 μM	Cabergoline tablets and urine	168
12	LC-FLD	Ergometrine	—	5–100 ng mL^−1^	14.9 μg kg^−1^	Rye flour and bakery products	169
		Ergometrinine			12.8 μg kg^−1^		
13	LC-ESI-MS/MS	Ergosine	—	0.40–4.99 ng g^−1^ and 0.45–12.17 ng g^−1^	0.25 ng g^−1^	Swine and dairy feeds	170
		Ergosinine		0.53–9.72 ng g^−1^ and 0.52–16.61 ng g^−1^	0.25 ng g^−1^		
14	UHPLC-MS/MS	Ergocristine	—	—	0.10 ng g^−1^	Cereal-based baby food	154
		Ergocristinine			0.07 ng g^−1^		
15	UHPLC-MS/MS	Ergocorninine	—	0.25–250 μg kg^−1^	0.0354 μg kg^−1^	Hard red spring wheat	171
		Ergocornine			0.225 μg kg^−1^		
16	UHPLC-MS/MS	Ergokryptine	—	1.5–100 μg kg^−1^	0.5 μg kg^−1^	Oat-based products	172
		Ergokryptinine		1.9–100 μg kg^−1^	0.6 μg kg^−1^		
17	LC-MS/MS	Ergocristine	—	0.5–100 μg kg^−1^	0.11 μg kg^−1^	Rye flour and wheat breadcrumbs	173
		Ergocristinine			0.12 μg kg^−1^		
18	HPLC-FLD	Ergotamine	—	—	2.2 μg kg^−1^	Compound feeds	174
		Ergotaminine			2.6 μg kg^−1^		
19	LC-MS/MS	Ergine	—	—	0.2 μg kg^−1^	Wheat-rye bread	175
		Erginine			0.3 μg kg^−1^		
20	UPLC-MS/MS	Ergometrine	—	8.39–200 μg kg^−1^ and 9.12–200 μg kg^−1^	2.54 μg kg^−1^ and 2.77 μg kg^−1^	Wheat and maize	176
		Ergometrinine		9.79–200 μg kg^−1^ and 7.64–200 μg kg^−1^	2.97 μg kg^−1^ and 2.32 μg kg^−1^		
21	TWIM-MS	Ergometrine	—	—	0.6 μg kg^−1^ and 0.5 μg kg^−1^	Barley and wheat	177
		Ergometrinine			0.3 μg kg^−1^ and 0.2 μg kg^−1^		
22	LC-MS/MS	Ergocornine	—	2.5–13.9 μg kg^−1^	0.4 μg kg^−1^	Cereal grains and its derivatives, wheat flour	178
		Ergocorninine		2.5–12.4 μg kg^−1^	0.2 μg kg^−1^		
23	UPLC-MS/MS	Ergosine	—	—	0.04 μg kg^−1^	Wheat, oat, rye, wheat gluten and baby food	179
		Ergosinine			0.03 μg kg^−1^		
24	LC-FLD	Ergocristine	—	5–100 ng mL^−1^	4.7 μg kg^−1^	Rye flour and bakery products	169
		Ergocristinine			1.5 μg kg^−1^		
25	LC-ESI-MS/MS	Ergocryptine	—	0.63–17.22 ng g^−1^ and 0.58–13.19 ng g^−1^	0.25 ng g^−1^	Swine feed and dairy feed	170
		Ergocryptinine		0.25–100.55 ng g^−1^ and 0.44–31.57 ng g^−1^	0.25 ng g^−1^		
26	UHPLC-MS/MS	Ergotamine	—	0.25–250 μg kg^−1^	0.0839 μg kg^−1^	Hard red spring wheat	171
		Ergotaminine			0.0645 μg kg^−1^		
27	LC-MS/MS	Ergosine	—	0.5–100 μg kg^−1^	0.14 μg kg^−1^	Rye flour and wheat breadcrumbe	173
		Ergosinine					
28	TWIM-MS	Ergometrine	—	3.2–100 μg kg^−1^	1.0 μg kg^−1^	Barley and wheat	172
		Ergometrinine		0.2–100 μg kg^−1^	0.1 μg kg^−1^		

#### 3.1.3 Fumonisins

Many fungal species, particularly cereal pathogenic fungi such as *F. verticillioides* and *F. proliferatum*, produce secondary metabolites known as fumonisins. *Aspergillus niger* generates fumonisins in peanuts, maize, and grapes. Most cereals and grain products contain fumonisins, with maize being the most common.^180,181^ Over 15 homologs of fumonisins have been identified, including types A, B, C, and P. Among these, fumonisin B_1_ (FB_1_), along with its harmful counterparts FB_2_ and FB_3_, is particularly concerning, as FB_1_ often coexists with the other two forms.^182^ FB_1_, FB_2_, and FB_3_ are the most common food contaminants. Propane-1,2,3-tricarboxylic acid (TCA) and 2-amino-12,16-dimethyl-3,5,10,14,15-pentahydroxyleicosane combine to form FB_1_, a diester with C-14 and C-15 hydroxyl groups and TCA carboxyl groups. FB_2_ and FB_3_ are C-5 and C-10 dehydroxy FB_1_. F. verticillioides, F. proliferatum, and a few other species are primarily responsible for the majority of fumonisin production. Globally, Fusarium saprophytes are present in soil and plants. Fusarium infection is present in the rhizosphere and surrounding systems.^79^ Infections in Zea mays are primarily caused by *Fusarium oxysporum*, ,*F. verticillioides* and *F. proliferatum*, which inflict damage on crops such as corn, carnation, chrysanthemum, and gladiolus throughout their production phase.^183^ Fusarium is responsible for infections in orchids, affecting both pathogenic and non-pathogenic varieties.^184^ Mutualists and nonpathogenic decomposers enhance seed germination and pigmentation. In certain crops, there is a reduction in non-pathogenic Fusarium wilt. Fusarium species important to agriculture, such as *F. oxysporum* and *F. solani*, are commonly found in soils that inhibit wilting.^185^ Before harvest, maize is susceptible to infection by Fusarium species, resulting in the production of fumonisins.^186^ Inadequate storage conditions lead to the production of fumonisin after harvest. Fumonisin has detrimental effects on both agricultural and laboratory animals. Horse leukoencephalomalacia, pig pulmonary oedema, rat hepatotoxicity, and nephrotoxicity are consequences of exposure to these toxins.^63^

Climate influences the contamination of food with fumonisins, particularly affecting crops such as oats, millet, rye, barley, maize, rice, and wheat, which are among the most contaminated. Foods that include FB_1_ include asparagus, garlic, barley, beers, dried figs, and milk. Most often, FB_1_ is found in maize and maize derivatives. Maize is a versatile ingredient used in numerous products, such as tortillas, cornflakes, popcorn, grits, flour, and oils. Research indicates that the levels of fumonisins FB_1_ and FB_2_ significantly decreased by 59% in tortilla chips made from maize flour, 60% in flour, and 50% in grits and snacks when subjected to extrusion heating. However, contamination with fumonisins persists in other products such as cornflakes, black and herbal teas, corn, and Portuguese maize bread.^187^ Endocrine disruptors harm young Nile tilapia. Fumonisins may have an impact on other aquatic creatures and plants, endangering food safety and security. They were found to alter the hepatic expression of the growth hormone receptor (GHR) and insulin-like growth factor 1 (IGF-1) in these species.^188^ In humans and animals, mycotoxins can cause poisoning produced by the fungi *Fusarium anthophilum*, *Fusarium proliferatum*, *Fusarium verticillioides*, *Fusarium napiform, Fusarium oxysporum*, *Fusarium dlamini*, and *Fusarium nygamai*.^63^ These toxins can be found in coffee, barley, sorghum, rice, and maize. Changes in the climate, such as dry periods followed by warm, rainy periods during flowering, can result in ear and kernel rot. Natural strains can reach the ear and kernels due to the insect's maturity damage. Rainfall before harvest could aggravate fumonisin infection in maize. Non-symptomatic maize kernels contain several fumonisins.^189^ In US studies, 34% of maize samples and 53% of diets based on corn contained FB_1_ and moniliformin.^190^ A 2007–2010 Brazilian study revealed the presence of FB_1_ and FB_2_ in 82% and 51% of corn-based foods, respectively. South Korean chicken broilers and feed-fatting calves had FB_1_ and FB_2_ contamination.^79^ This pollutant is found in roasted coffee beans, green coffee beans, buckwheat flour, rye, oats, and wine. A variety of methods are used for the detection of fumonisins in various analytes as discussed in the subsequent section.

##### 3.1.3.1 Detection of fumonisins

Dhiman *et al.* developed an electrochemical microfluidic biosensing technology for the detection of FU-B1, utilizing a maskless lithography approach with a silver-ceria nanocomposite. Structural characterization confirmed the successful creation of impurity-free ceria (CeO_2_) nano-cubes and spherical silver structures. The sensing figures of merit of Ag–CeO_2_ nanocomposite-based microfluidic biosensor stem from rapid electron transfer facilitated by the synergistic conductivity of Ag nanoparticles and the redox-active surface of CeO_2_ nanocubes, which enhances charge mobility at the transducer–analyte interface. Specific binding occurs *via* hydrogen bonding and electrostatic interactions between the functional groups of fumonisin-B1 and the nanocomposite surface, promoting selective and efficient electron exchange. The nano-biochip exhibited a linear response to the antigen FU-B_1_, with a detection range spanning from 10 pg mL^−1^ to 100 ng mL^−1^ and a sensitivity of 7.33 μA log(ng mL^−1^)^−1^cm^−2^. The limits for quantification and detection were determined to be 3.9 and 1.5 pg mL^−1^, respectively.^191^ Munawar *et al.* introduced an electrochemical sensor for measuring FB_1_ in maize samples, employing molecularly imprinted nanoparticles (nanoMIPs) as the recognition unit. The enhanced current arises from efficient electron transfer of the redox couple through the highly conductive PPy/ZnP underlayer and the analyte-modulated nanoMIP film *via* a gate-effect mechanism, where FB_1_ binding triggers local polymer swelling and increased porosity at the Pt interface; FB_1_ is selectively captured by hydrogen bonding and electrostatic interactions between its carboxylate/hydroxyl groups and the phosphate/amide functionalities of the EGMP and NAPMA monomers in the imprinted cavities This nanoMIPs-based chemosensor demonstrated superior performance in terms of sensitivity, low detection limits, reproducibility, repeatability, and shelf life compared to previous research.^192^ Another study used a single pot to synthesize AuNPs-doped 2D titanium carbide MXene nanoflakes (Ti_3_C_2_T_*x*_/Au). The synergistic signal amplification and superior electrical conductivity of Ti_3_C_2_T_*x*_ MXene and AuNPs led to enhanced electrochemical performance. The Ti_3_C_2_T_*x*_/Au hybrid nanostructure is therefore an effective electrode platform for the electrochemical analysis of various targets. To identify and measure ampicillin (AMP) and FB_1_, SPE with the Ti_3_C_2_T_*x*_/Au configuration and biorecognition components was employed. The detection limits of 2.284 pM and 1.617 pg mL^−1^ for AMP and FB_1_, respectively, are significantly lower than the US FDA's maximum residual limits of 2.8 nM in milk and 2 to 4 mg kg^−1^ in maize products for human consumption. Furthermore, 10 pM to 500 nM and 10 pg mL^−1^ to 1 μg mL^−1^ were chosen as the linear ranges for AMP and FB_1_ detection and quantification, respectively.^193^

Dong *et al.* developed a tetrahedral DNA nanostructure (TDN) electrochemical aptasensor that demonstrated a remarkable sevenfold dynamic range, enabling the detection of FB_1_ at sub-femtogram per milliliter (fg mL^−1^) concentrations. The 5.67 nm TDN was specifically engineered to immobilize FB_1_ aptamers effectively. This approach adjusted TDN assembly density to make the sensing interface assembly controllable and reliable. TDN anchored the aptamer at the electrode, whereas MB absorption generated the signal. To reduce free FB_1_ and increase the sensing system's dynamic range, a helper aptamer was added to the sample solution, which negatively charged the TDN blocks and inhibited the absorption of the aptamer. The sensing performance can be related to the tetrahedral DNA scaffold precisely positioning methylene blue labels near the gold electrode to create a low-impedance, high-density redox interface. At the same time, fumonisin B_1_ binding to its aptamer *via* hydrogen bonds and π–π stacking triggers aptamer stripping (reducing steric and electrostatic blocking) and thus enables rapid electron transfer and amplified current. The linear dynamic range of the sensor was 0.500 to 1.00 ng mL^−1^, which was the lowest detection limit of any existing method.^194^ Jin *et al.* used bipolar electrodes (BPEs) to analyze FB_1_ on an array-based ECL platform visually. The sensor consists of a PDMS cover and a glass substrate with 10 ITO electrodes. A unique sensing interface on the BPE cathode modulates ECL reactions at the anode. The current intensification can be linked to the dense loading of reversible methylene-blue redox centers within porous Zr-MOFs grafted onto highly conductive Au-NP/ITO transducers, which drastically lowers interfacial impedance and boosts electron flux. At the same time, fumonisin B_1_ binds its aptamer *via* hydrogen bonds and π–π stacking to trigger DNA-walker/nicking enzyme cycling that modulates MB@Zr-MOF release and amplifies the faradaic signal. The biosensor visibly quantifies FB_1_ from 5 × 10^−5^ to 0.5 ng mL^−1^ utilizing the cyclic amplification activity of the DNA walker and nicking endonuclease. The biosensor detected FB_1_ in samples of peanuts and corn. The BPE-ECL biosensor exhibits a recovery rate of 99.2% to 110.6%, indicating its high accuracy for FB_1_ detection in foodstuffs.^195^

Zheng and coworkers developed a label-free DNA electrochemical aptasensor to detect FB_1_ in maize. The electrochemical signal was enhanced, and the FB_1_ identification probe was anchored using chemically modified AuNPs and GO. Using the π–π interaction between thionine and GO, thionine—a potent electrochemical indicator—was conjugated onto the surface of the GO–Au complex. The aptamer was functionalized onto gold particles *via* an Au–S link to improve FB_1_ measurement specificity and reduce interference during real sample analysis. The proposed biosensor has a low detection limit of 10 pg mL^−1^ and a linear range of 1 × 10^−11^ to 1 × 10^−4^ g mL^−1^. In real sample detection, it demonstrated commendable practicality and durability.^196^ Yu *et al.* developed a disposable bipolar electrode (BPE)-electrochemiluminescence (ECL) device to detect FB_1_. MWCNTs and PDMS were used to produce BPE due to their higher electrical conductivity and mechanical rigidity. The synergistic conductivity of MWCNTs and AuNPs leads to enhanced electron transfer and a larger surface area at the cathode. Meanwhile, the specific binding of FB_1_ to the thiolated aptamer involves hydrogen bonding and Ag–S covalent bonding, modulating oxygen reduction and amplifying the ECL signal at the anode through a dual-signal amplification mechanism. Au NPs on the BPE cathode increased the ECL signal by an 89-fold factor. An aptamer-based sensing technique was developed by grafting capture DNA onto the Au surface and hybridizing it with the aptamer. When tagged on an aptamer, Ag NPs catalyzed the oxygen reduction reaction, increasing the ECL signal at the BPE anode by 13.8-fold. Under ideal conditions, the biosensor detected FB_1_ concentrations ranging from 0.10 pg mL^−1^ to 10 ng mL^−1^ linearly.^197^ TDNs were employed to precisely bind an Apt in a paper-based electrochemical aptasensor, enhancing its target recognition capabilities. To immobilize complementary DNA–TDNs, AuNPs@MXenes, a sensing substrate with excellent conductivity, were modified on the electrode. With the help of many Apt–Au@Pt nanocrystals built on the sensing interface and hybridized with cDNA, TMB was able to oxidize with H_2_O_2_ and generate a significantly higher DPV signal in the absence of FB_1_. When the target FB_1_ linked to its Apt, the electrochemical signal was reduced due to the release of Apt–Au@Pt NCs from double-stranded DNA (dsDNA). Under ideal circumstances, FB_1_ triggered the strand displacement response, resulting in a lower detection limit (21 fg mL^−1^) and a more extensive dynamic linear range (50 fg mL^−1^ to 100 ng mL^−1^) for the aptasensor.^198^

**Fig. 7:**
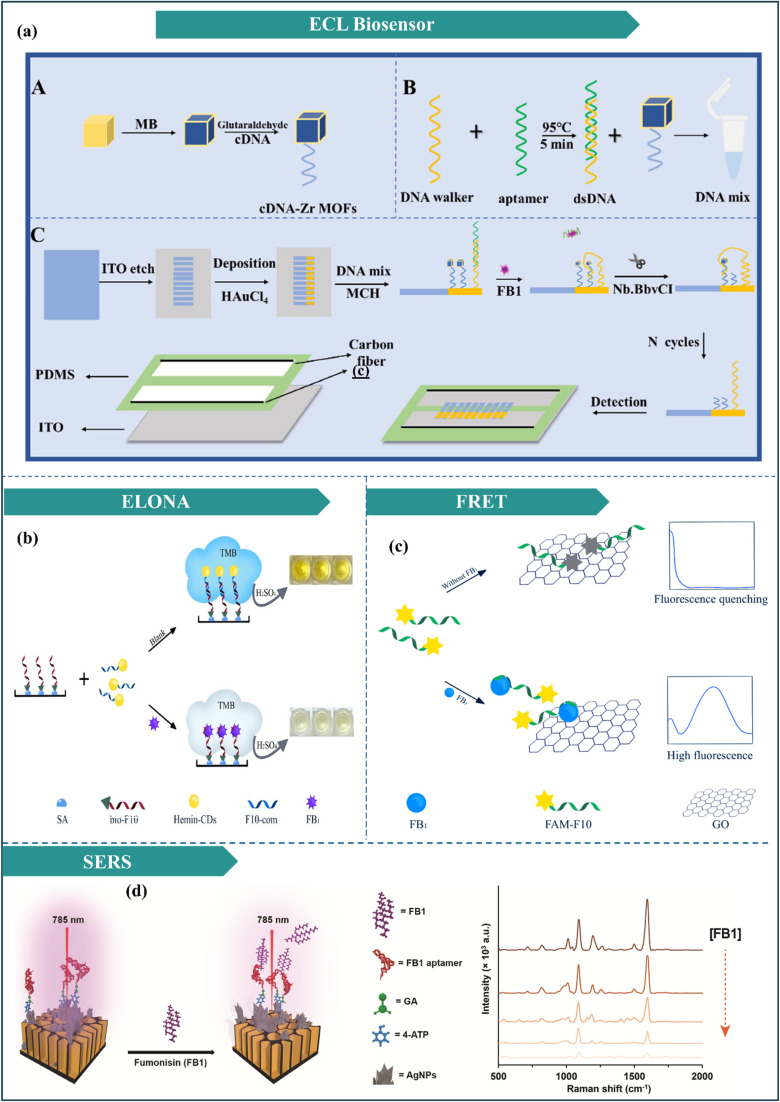
ECL Biosensor: (a) Schematic diagram of (A) the preparation process of cDNA-Zr MOFs, (B) synthesis process of DNA mix, and (C) visual bipolar electrode-ECL array electrode for FB_1_ detection, reproduced from ref. 195 with permission from MDPI, copyright 2023. Enzyme-linked oligonucleotide assay (ELONA): (b) schematic diagram of the ELONA method based on aptamer complementary chains and CDs conjugates, reproduced from ref. 199 with permission from MDPI, copyright 2022. FRET: (c) schematic diagram of the FRET method for detecting FB_1_ based on the aptamer, reproduced from ref. 200 with permission from MDPI, copyright 2022. SERS: (d) schematic representations of FB_1_ (target analyte) capturing and detection using aptamer modified Ag-pSi SERS scaffold, reproduced from ref. 201 with permission from Elsevier, copyright 2025.

The detection of FB_1_, a hazardous mycotoxin prevalent in food commodities, has been significantly advanced through innovative biosensing technologies, as illustrated in Fig. 7. Electrochemiluminescence (ECL) biosensors (Fig. 7a) integrate Zr-based metal–organic frameworks and visual bipolar electrode arrays to achieve highly sensitive and spatially resolved FB_1_ detection, combining the advantages of MOF-enhanced signal loading with ECL's low background noise. Enzyme-linked oligonucleotide assays (ELONA) (Fig. 7b) utilize aptamer complementary chains and carbon dot conjugates, offering a robust and improved method for FB_1_ quantification by combining nucleic acid hybridization with enzymatic signal amplification. Förster resonance energy transfer (FRET)-based methods (Fig. 7c) employ aptamer-mediated fluorescence quenching, allowing precise FB_1_ detection through distance-dependent energy transfer between fluorophores. Lastly, surface-enhanced Raman spectroscopy (SERS) platforms (Fig. 7d) utilize aptamer-modified silver-coated porous silicon (Ag-pSi) substrates to achieve ratiometric and reusable FB_1_ detection, leveraging the plasmonic enhancement of Raman signals for ultrasensitive and multiplexed analysis. Together, these biosensing approaches highlight the integration of nanomaterials, biorecognition elements (such as antibodies and aptamers), and advanced transduction mechanisms (including electrochemical, optical, and spectrometric) to address the challenges of FB_1_ detection in food safety, paving the way for portable, multiplexed, and real-time monitoring systems.

A light-driven biosensor designed for the detection of FB_1_ features a hybrid nanoantenna sensing interface. This interface is formed by integrating graphdiyne oxide composites infused with methylene blue and gold nanorods (GDYO-MB-Au NRs) with cadmium selenide quantum dots and DNA nanowires. A TDN, which was both light-driven and amplified, enabled this incorporation. At the sensor interface, MB's chemical characteristics were changed and degraded by light-driven hybrid nanoantennas. Contact with FB_1_ may remove the CdSe QDs-DNA nanowire, compromising light-driven activity and enhancing the electrochemical signal by blocking MB degradation. The hybrid nanoantenna was incorporated into the biosensor's design to photodegrade MB, thereby reducing the background signal and improving detection sensitivity. The increase in peak current in this light-driven electrochemical biosensor is attributed to the enhanced electron transfer facilitated by the hybrid nanoantenna, where GDYO-MB-Au NRs amplify conductivity and surface reactivity. At the same time, CdSe quantum dots–DNA nanowires modulate the photodegradation of MB. Specific binding of FB_1_ to the DNA tetrahedral structure involves hydrogen bonding and π–π stacking, which disrupts MB degradation and boosts the electrochemical signal. The produced biosensor attained a linear detection range of 0.5 to 10 pg mL^−1^ and a detection limit of 0.45 fg.^202^ By electrodepositing AuNPs on chitosan-functionalized nitrogen-doped graphene and polyaniline (N-G@PANI@CS) electrodes, Yang *et al.* developed an electrochemical immunosensor for detecting mycotoxin FB_1_ in food. The conductivity and high electroactive surface area of the AuNPs/N-G@PANI@CS composite significantly lower charge-transfer resistance and accelerate electron flow; antibodies are covalently tethered to AuNPs *via* Au–S bonds, and FB_1_ binds its antibody through non-covalent hydrogen bonding and electrostatic/hydrophobic interactions, forming immunocomplexes that modulate the redox probe signal. Under ideal conditions, the immunosensor exhibits a linear response from 0.50 to 800.00 ng mL^−1^, with a limit of detection (LOD) of 0.07 ng mL^−1^ and a limit of quantification (LOQ) of 0.22 ng mL^−1^. The designed electrochemical immunosensor had 3.26% to 4.73% RSD in real corn samples. The recovery rate ranged from 93.97% to 98.62%, indicating its suitability for FB_1_ detection in corn samples. Sensor stability and specificity were also impressive.^203^Table 3 enlists a variety of detection methods and electrode modifiers for Fumonisins, along with their detection limits.

**Table 3:** Methods of fumonisins detection and their analytical parameters

S. No.	Method	Analyte	Electrode modifier	Linear range	LOD	Samples/recovery	Ref.
1	DPV	FB_1_	Pt/(PPy/ZnP)/nanoMIPs	1 fM–10 pM	0.03 fM & 0.7 fM	Maize/96–102%	192
	EIS						
2	DPV	FB_1_	Exo-I/Apt/cDNA/AuE	0.001–1000 ng mL^−1^	0.15 pg mL^−1^	Beer/upto 98.6%	204
	EIS					Corn/upto 106.8%	
3	EIS	FB_1_	FB_1_/DNA/PGE	2.5–10 μg mL^−1^	3.69 ng mL^−1^	—	205
4	EC	FB_1_	—	10 pg mL^−1^–100 ng mL^−1^	1.5 pg mL^−1^	Corn/85%	191
5	DPV	FB_1_	AuNPs-SE	1 ng L^−1^–1 mg L^−1^	0.08 ng L^−1^	Pork and beef/89.7–113.3%	206
6	DPV	FB_1_	Apt_2_-AuNRs-Fc/Apt_1_-AuNRs-Th/cDNA/AuE	1 pg mL^−1^–100 ng mL^−1^	0.26 pg mL^−1^	Beer/89–102%	207
7	CV	FB_1_	—	50–400 μg L^−1^	84.17 μg L^−1^	Maize-based and baby foods	208
8	CV	FB_1_	ss-HSDNA/AuNPs/SPCE	0.5–500 ng mL^−1^	0.14 ng mL^−1^	Maize	209
9	ECL	FB_1_	FB_1_/DNA mix/Au NPs/ITO BPE	5 × 10^−5^–0.5 ng mL^−1^	0.67 pM	Maize/99.6–104.1%	195
						Peanut/99.2–110.6%	
10	CV	FB_1_	GCE/Au-GO-Th/DNAA/FB_1_	1 × 10^−11^-1x10^−4^ g mL^−1^	10 pg mL^−1^	Corn	210
11	EC	FB_1_	Apt/MB/MCH/TDN/Au	0.500 fg mL^−1^–1.00 ng mL^−1^	0.306 fg mL^−1^	Rice/97.9–108%	192
12	EC	FB_1_	Ti_3_C_2_T_*x*_/Au/l-Cys/anti- FB_1_	10 pg mL^−1^–1 μg mL^−1^	1.617 pg mL^−1^	Corn/102.9–103.8%	193
						Wheat/100.3–107.2%	
13	EC	FB_1_	Silver/FB_1_/Au-aptamer/Fe_3_O_4_-capture DNA/GCE	10^−3^–10 ng mL^−1^	—	Corn/109–103.8%	211
14	EC	FB_1_	BSA/ab-FB_1_/GOMn_3_O_4_/ITO	1 pg mL^−1^–800 ng mL^−1^	0.195 pg mL^−1^	Sweet corn/98.91%	212
15	PEC	FB_1_	MIP/GO/CdS/CS/ITO	0.01–1000 ng mL^−1^	4.7 pg mL^−1^	Maize/94.03–102.97%	213
						Milk/94.61–105.81%	
16	EC	FB_1_	—	10 pg mL^−1^–100 ng mL^−1^	—	Corn/85%	214
17	CV	FB_1_	Anti- FB_1_/AuNPs/N-G@PANI@CS/GCE	0.50 ng mL^−1^–800 ng mL^−1^	0.07 ng mL^−1^	Corn/93.97–98.62%	203
	EIS						
18	DPV	FB_1_	—	50 fg mL^−1^–100 ng mL^−1^	21 fg mL^−1^	Corn and wheat	198
19	CV	FB_1_	—	0.3–140 ppb	97 pg mL^−1^	Ground corn/88.6–95.8%	215
20	LC-MS/MS	FB_1_	cDNA-AuNR/aptamer-Cy5.5	10–250/500 pg mL^−1^	3/5 pg mL^−1^	Corn/92–107%	216
21	Ratiometric EC	FB_1_	FB_1_/TDN/MCH/cDNA/AuE	0.1–100 pg mL^−1^	0.087 pg mL^−1^	Rice powder/97.8–102%	217
22	DPV	FB_1_	MIP-F/ITO/PANI	1 pg mL^−1^–500 ng mL^−1^	0.322 pg mL^−1^	Corn/86.16–102.41%	218
23	EC	FB_1_	GCE/GDYO-MB-Au NRs/CdSe QDs	0.5 fg mL^−1^–10 pg mL^−1^	0.45 fg mL^−1^	Rice/95.4–97.6%	202
						Glutinous rice/96–100%	
24	SERS	FB_1_	α FB_1_/4-ATP/Ag-pSi surface	0.1–1000 ppb	0.05 ppb	Maize/97.6–106.4%	201
						Onion/93.4–109.8%	
						Wheat/93.3–107.4%	
						Defatted milk/98.1–113.6%	
25	FRET	FB_1_	FAM-F10/GO/FB_1_	0–3000 ng mL^−1^	14.42 ng mL^−1^	Corn/89.13–102.08%	200
26	ELONA	FB_1_	F10-com-hemin-CDs	0–100 ng mL^−1^	4.30 ng mL^−1^	Corn/97.5–99.23%	199
27	Fluorometry	FB_1_	—	0.01–100 ng L^−1^	0.003 ng L^−1^	Corn/106.6%	219
28	PEC	FB_1_	Apt/MnFe_2_O_4_/MCH/sDNA/Cu_2.46_Co_0.54_(HITP)_2_/GCE	0.1 pg mL^−1^–10 ng mL^−1^	65 fg mL^−1^	Corn/96.4–103%	220
						Flour/98.7–108%	
29	ECL	FB_1_	FB_1_/Ag NPs/aptamer/MCH/capture DNA/Au	0.10 pg mL^−1^–10 ng mL^−1^	—	Corn/104.8–112.8%	197
						Peanut/90.9–112.1%	

#### 3.1.4 Trichothecenes

Fusarium fungi produce sesquiterpenoid mycotoxins known as trichothecenes (T-2 toxin and HT-2 toxin).^221^ These toxins can contaminate agricultural products, threatening food safety and public health. Toxigenic organisms produce TCT because of genetic and environmental factors. In developing countries, fungal proliferation and mycotoxin production are significantly influenced by monsoons, unseasonal rainfall during harvest, flash floods, and temperature as well as moisture level.^222^ According to research, crops grown in tropical and subtropical regions are more susceptible to mycotoxin contamination, including TCT, than those grown in temperate areas because these regions provide optimal conditions for toxin production.^223^ Due to weather and environmental factors, TCT contamination of feeds and food varies geographically and temporally across continents, regions, and climatic zones. Various crop varieties, agricultural practices, post-harvest storage methods, processing techniques, soil types, and management practices contribute to local variations. Evaluation of co-exposure to diverse TCTs is crucial for risk characterization, as animals and people consume varied diets.^224^ Mycotoxin exposure is a severe health concern and productivity constraint for animals, especially monogastric species. Subclinical consequences, including degradation of intestinal integrity and changes in gut flora, are concerning due to their low prevalence. The assessment and management of fungal metabolite hazards to human and animal health have improved as a result of European monitoring procedures for major mycotoxins in food and feed, including TCT.^225^ Trichothecenes are a large family of toxic sesquiterpenoids. Trichothecenes of types A and B are widespread and harmful.^226^

##### 3.1.4.1 Detection of TCT

Solis *et al.* developed a competitive immunoassay for detecting T-2 toxin by utilizing monoclonal *anti*-T-2 antibodies within a PMMA microfluidic central channel.^224^ At the end of the channel, a platinum wire working electrode was enhanced through a one-step electrodeposition technique that incorporated rGO-nanoporous gold. In this setup, the T-2 toxin present in the sample competed with a T-2-horseradish peroxidase conjugate for binding sites on the immobilized *anti*-T-2 monoclonal antibodies. When exposed to H_2_O_2_, HRP oxidizes 4-*tert*-butylcatechol (4-TBC) and reduces it electrochemically at −0.15 V on the nanostructured electrode. Thus, with lower T-2 concentrations, more enzymatically conjugated T-2 should adhere to the capture antibodies, thereby increasing the current. Electron transfer is accelerated by the 3D graphene-decorated nanoporous gold film, whose enormous electroactive surface area and superior conductivity dramatically lower the charge-transfer resistance and amplify the HRP–H_2_O_2_–4-*tert*-butylcatechol quinone redox current in the competitive immunoassay. Antibodies are covalently tethered *via* PEI/glutaraldehyde and Au–S anchoring, while T-2 toxin binds its specific antibody through noncovalent hydrogen bonds, electrostatic, and hydrophobic interactions. The commercial ELISA method and the electrochemical immunosensor have detection limits of 10 μg kg^−1^ and 0.10 μg kg^−1^, respectively. Furthermore, the coefficients of variation within and between assays were less than 5.35% and 6.87%, respectively.^227^ An inkjet-printed electrochemically reduced graphene oxide (rGO) microelectrode for HT-2 mycotoxin immunoenzymatic biosensing was constructed and utilized, as reported in another study. Following solvent evaporation, single-drop line working microelectrodes with dimensions of 78 μm in width and 30 nm in height were inkjet-printed onto poly (ethylene 2,6-naphthalate) substrates using a water-based GO ink. DPV was used to detect 1-naphthol (1-N) by modifying the electrochemical reduction parameters. Reduction timings greatly affected electrode performance. The biosensor exhibited a linear dynamic range of 6.3 to 100.0 ng mL^−1^ and a detection limit of 1.6 ng mL^−1^ during a 5-minute incubation period using the 1-naphthyl phosphate (1-NP) substrate.^228^

Another study utilized AgMOFs and N-CDs to develop a sensitive trilobatin electrochemiluminescence (ECL) sensor. The sensor used AgMOF@N-CD composite as the luminophore, as the integration of both components enhances the stability and intensity of N-CD ECL. AgMOFs exhibited high specific surface area, porosity, and adsorption capabilities, whereas N-CDs displayed high ECL intensity but low stability. The high conductivity of AgMOF@N-CD composite, large porosity, and abundant N-doped sp^2^ sites minimize charge-transfer resistance and accelerate electron injection into trilobatin, whose multiple phenolic –OH groups coordinate to Ag^+^ centers and form hydrogen bonds and π–π stacking with the N-CD matrix to lock the analyte in place and facilitate interfacial electron transfer. Under ideal conditions, the ECL sensor detected trilobatin with a low detection limit of 5.99 × 10^−8^ M (S/N = 3), and a linear range of 1.0 × 10^−7^ M to 1.0 × 10^−3^ M. Additionally, the sensor exhibits excellent stability, interference resistance, and reproducibility.^229^ A label-free aptasensor detects vomitoxin in grains. Cerium-based MOF composite AuNPs (CeMOF@Au) supplied electron transport substrates and DNA binding sites. The CeMOF@Au composite, with dense AuNPs grown on a porous Ce-MOF scaffold, greatly increases electroactive surface area and conductivity lowering charge-transfer resistance and accelerating [Fe(CN)_6_]^3−/4−^ shuttling, while Exo III-mediated cDNA recycling further amplifies the electron flux. Thiolated DNA is covalently anchored to AuNPs *via* Au–S bonds, and DON–aptamer recognition is driven by hydrogen bonding and van der Waals (electrostatic) interactions between the toxin's hydroxyl/epoxide groups and the oligonucleotide bases, ensuring specific target capture and signal modulation. With a detection limit of 1.79 × 10^−9^ mg mL^−1^ and an excellent recovery rate in maize, samples spiked with vomitoxin, the aptasensor can detect vomitoxin in the range of 1 × 10^−8^ to 5 × 10^−4^ mg mL^−1^ under optimal conditions.^230^

**Fig. 8:**
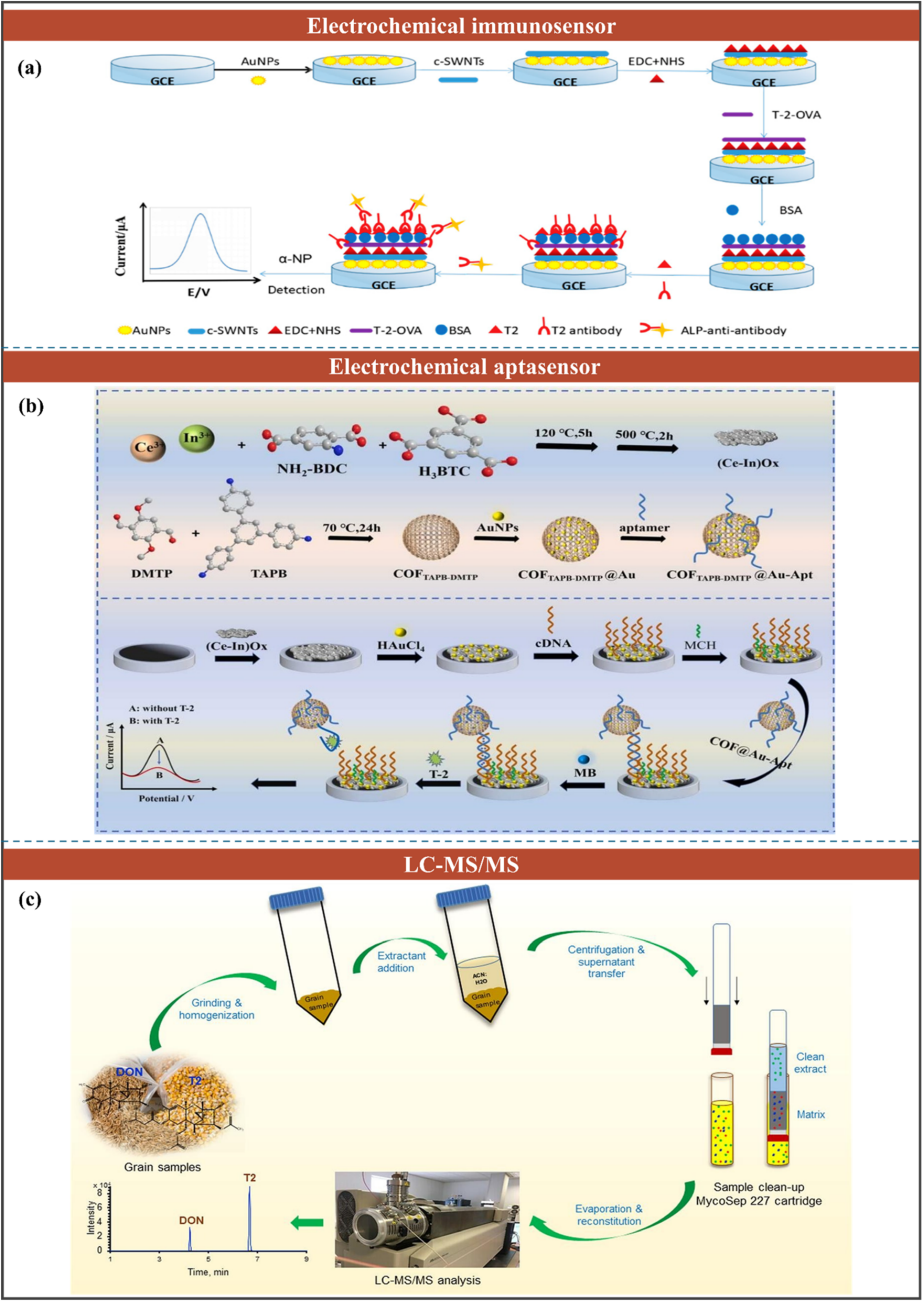
Electrochemical immunosensor: (a) the fabrication process of electrochemical biosensor SWNTs/CS/AuNPs/GCE for the detection of T-2 toxin, reproduced from ref. 231 with permission from MDPI, copyright 2018. Electrochemical aptasensor: (b) schematic diagram for the determination of T-2 toxin using a dual-signal amplification, reproduced from ref. 232 with permission from Elsevier, copyright 2023. LC-MS/MS: (c) schematic representation for the simultaneous determination of deoxynivalenol and T-2 toxin in maize and oats, reproduced from ref. 233 with permission from Elsevier, copyright 2021.

The analytical approaches for detecting T-2 toxin, a highly toxic type A trichothecene mycotoxin, are illustrated in Fig. 8, which showcases three distinct yet complementary detection strategies. Electrochemical immunosensors (Fig. 8a) employ a sophisticated nanocomposite platform comprising single-walled carbon nanotubes (SWNTs), chitosan (CS), and gold nanoparticles on a glassy carbon electrode, which enhances both the surface area for antibody immobilization and electron transfer efficiency, enabling sensitive T-2 toxin detection in complex matrices like feed and swine meat. Electrochemical aptasensors (Fig. 8b) employ a dual-signal amplification strategy that involves bimetallic oxide (Ce–In)O_*x*_ and covalent organic frameworks, significantly enhancing detection sensitivity through synergistic effects that improve both catalytic activity and aptamer loading capacity. For confirmatory analysis and multi-mycotoxin detection, liquid chromatography-tandem mass spectrometry (LC-MS/MS) (Fig. 8c) provides a gold-standard approach, offering high specificity and accuracy for the simultaneous quantification of T-2 toxin and deoxynivalenol in cereals like maize and oats. Together, these methods illustrate the evolution from conventional antibody-based assays to advanced nanomaterial-enhanced biosensors and exact chromatographic techniques, addressing the critical need for reliable T-2 toxin monitoring across different food and feed commodities. The integration of nanomaterials in electrochemical platforms and the robustness of mass spectrometry highlight the complementary roles of screening and confirmatory methods in comprehensive mycotoxin analysis.

Since T-2 toxin is a significant pollutant of crops, stored grains, and food products, its identification is essential for both human and environmental health. Nanoelectrode arrays are used as gate photoactive materials in the zero-gate-bias organic photoelectrochemical transistor (OPECT) sensor. Due to its extraordinary signal amplification, OPECT's channel current was an order of magnitude higher than that of a typical photoelectrochemical system's photocurrent. Ordered ZnO nanorod arrays act as densely packed nanoelectrodes that, under illumination, generate a large photovoltage and facilitate rapid, low-resistance electron transfer to the FTO gate thanks to their high electroactive surface area and double-layer capacitance. The OPECT channel then amplifies these slight gate-potential shifts into large drain-current outputs. Aptamer probes are covalently anchored *via* Schiff-base (–NH_2_/–CHO) linkages, and T-2 toxin is captured through hydrogen bonding and van der Waals interactions between its hydroxyl/epoxide groups and the oligonucleotide bases. The OPECT aptasensor's detection limit is 28.8 pg L^−1^, which is significantly lower than the standard PEC method's limit of 0.34 ng L^−1^. This indicates that OPECT devices are more effective at measuring T-2 toxins.^234^ The Fusarium genus produces T–2 toxin, a type A TCT mycotoxin that is genotoxic, cytotoxic, and immunotoxic to animals and humans. As a result, a quick testing process with high reliability, sensitivity, and selectivity is required. By carefully modifying the synthesis parameters, Zhao *et al.* discovered a form of NH_2_-UiO-66 with a high quenching efficiency. They used a Cy3-labeled aptamer to develop a new fluorescence sensor. NH_2_-UiO-66 may adsorb and quench Cy3–aptamer fluorescence using FRET and PET principles through coordination, hydrogen bonding, and π–π interactions. The NH_2_-UiO-66/Cy3-aptamer complex was disrupted by the T-2 toxin, which was covalently linked to the Cy3-aptamer.

Zhao *et al.* demonstrated that inhibiting energy transfer restored fluorescence intensity, facilitating a sensitive response to T–2 toxin. Their study revealed a strong linear correlation between T-2 toxin concentrations (ranging from 0.5 to 100 ng mL^−1^) and fluorescence intensity. The developed fluorescent aptasensor exhibited a signal-to-noise ratio of three and achieved a detection limit of 0.239 ng mL^−1^. Additionally, the recovery rates for beer and milk samples were found to be 92.31–111.51% (RSD = 2.3–2.9%) and 89.86–108.99% (RSD = 2.0–2.6%), respectively.^235^ Zhang *et al.* developed a colorimetric aptasensor for T-2 toxin detection utilizing pristine Au NPs and an ssDNA aptamer. The solution remained red because the aptamer-wrapped AuNPs inhibited salt-induced aggregation when T-2 was not present. T-2 was added, and the aptamer was complexed with it before being separated from the AuNPs. In a saline environment, this interaction caused the AuNPs to aggregate and change from red to a purple-blue color. The dense AuNPs film on the electrode–aptamer interface, which multiplies electroactive surface area slash charge-transfer resistance and speeds faradaic electron flow, while T-2 binding induces an aptamer fold that further gates tunneling currents; the toxin's hydroxyl and epoxide moieties engage complementary bases *via* multiple hydrogen bonds and van der Waals contacts to secure specific recognition and signal modulation. T-2 was detected by the aptasensor with excellent selectivity and sensitivity. The detection limit and linearity range were 57.8 pg mL^−1^ (0.124 nM) and 0.1 to 5000 ng mL^−1^, respectively.^236^ The detection of TCT by different biosensing technologies can be seen in Table 4.

**Table 4:** Detection of TCT using their LODs as determined by various analytical methods

Sr No.	Method	Analyte	Electrode modifier	Linear range	LOD	Samples/recovery	Ref.
1	ECL	Deoxynivalenol (DON)	Ab-biconjugates/BSA/Ag/APTES-PTCA@CNHs/GCE	0.1 ng mL^−1^–20 ng mL^−1^	0.03 pg mL^−1^	Milk/89.3–102.4%	237
2	CV	DON	MIP-SPGE	5–500 ng mL^−1^	0.3 ng mL^−1^	Cornflakes/90.8–98.5%	238
	EIS						
3	EC	DON	—	610–6210 μg kg^−1^	342.4 μg kg^−1^	Wheat/78–79%	239
4	EC	DON	DON/Anti_DON_/Gel/AE	0.001–0.5 ng mL^−1^	0.43 pg mL^−1^	Apple juice/95–109%	240
5	EC	HT-2	Anti-HT-2 Fab/ERGO	6.3–100.0 ng mL^−1^	1.6 ng mL^−1^	Bovine serum/87.8–108.5%	228
6	EC	T-2	rGO-NPG/Pt	0–1000 μg kg^−1^	0.10 μg kg^−1^	Wheat germ	227
7	Aptasensor	DON	—	1 fg mL^−1^–1 ng mL^−1^	2.11 pg mL^−1^	Plant extract	241
8	Electrochemical DNA sensor	T-2	Aptamer/MCH/CP/MoS_2_-PANI-Chi-Au/GCE	10 fg mL^−1^–100 ng mL^−1^	1.79 fg mL^−1^	Canned beer/94.62–103.52%	242
9	Fluorescence NPs based aptasensor	T-2	T-2/aptamer/KYF_4_:Tb^3+^	0.001–100 ng mL^−1^	0.33 pg mL^−1^	Maize/90.0–92.0%	243
10	Silver nanoclusters-based FRET aptasensor	T-2	NC-T5-apt-AgNCs/MoS_2_/T-2	0.005–500 ng mL^−1^	0.93 pg mL^−1^	Maize/89.46–102.08%	244
						Wheat/90.41–107.75%	
11	GC-QqQ-MS/MS	DON	—	1.3–1.6 μg kg^−1^	0.1 μg kg^−1^	Chicken liver/87.9–105.7%	245
		3-ADON				—/92.4–108.4%	
		15-ADON				—/89.2–96.7%	
12	ID-LC/MS/MS	DON	—	0.01–10 μg kg^−1^	0.11 μg kg^−1^	Grain	246
		DEG			0.02 μg kg^−1^		
		NIV(Nivalenol)			0.14 μg kg^−1^		
13	GC-QqQ-MS/MS	NIV	—	2.6–7.9 μg kg^−1^	0.05 μg kg^−1^	Chicken liver/97.4–103.7%	245
		NEO		2.7–25 μg kg^−1^	0.15 μg kg^−1^	—/88.6–95.4%	
14	GC-QqQ-MS/MS	HT-2	—	3.7–13.3 μg kg^−1^	0.15 μg kg^−1^	Chicken liver/96.5–112.3%	245
		T-2				—/98.1–101.5%	
15	EC immunosensor	T-2	ALP-*anti*-antibody/anti-T-2/T-2-SWNTs/CS/AuNPs/GCE	0.01–100 ng mL^−1^	0.14 μg L^−1^	Maize/91.59–102.49%	231
						Swine meat/91.42–100.80%	
16	FRET	T-2	—	0.001–100 ng mL^−1^	0.57 pg mL^−1^	Maize/90–107.77%	247
17	UCNP/MOF fluorescence aptasensor	T-2	—	0.1–100 ng mL^−1^	0.087 ng mL^−1^	Corn meal/97.52–109.53%	248
						Beer/90.81–100.02%	
18	Single cell EC	T-2	GelMA/AuNP/GCE	0–1000 ng mL^−1^	0.13807 ng mL^−1^	Flour/81.19–130.17%	249
19	UPLC-MS/MS	DON T-2	—	10–800 μg kg^−1^	0.10 μg kg^−1^ and 0.12 μg kg^−1^	Maize/89.6–93.3% and Oat/86.3–87.7%	233
				5–200 μg kg^−1^	0.04 μg kg^−1^	Maize/86.1–95.3% and oat/85–92.4%	
20	Photo induced OECT	T-2	T-2/Apt/ZnO NEAs	100 pg L^−1^–1 mg L^−1^	28.8 pg L^−1^	Milk/97.8–102.4%	250
21	Cy3–aptamer/MOFs fluorescence aptasensor	T-2	—	0.5–100 ng mL^−1^	0.239 ng mL^−1^	Milk/89.86–108.99%	235
						Beer/92.31–111.51%	
22	TRFIA	T-2	—	0.0625–50 ng mL^−1^	0.052 ng mL^−1^	Corn and feed substrate/95.31–119.03%	251
					0.071 ng mL^−1^		
23	EIS	DON	Au/Cys/PAMAM/MAb	50–2500 ppb	50 ppb	Dry pasta/98.75–102.60%	252
	DPV			1–5000 ppb	1 ppb		
24	EC	T-2	MCH/S-DNA/AuNPs/MnO_2_@GO/AuE	2 fg mL^−1^–20 ng mL^−1^	0.107 fg mL^−1^	Milk/96.5–103.4%	232
25	DPV	T-2	T-2/MB/COF_TAPB-DMTP_@Au-apt/MCH/cDNA/AuNPs/(Ce–In)Ox/GCE	5.0 × 10^−7^–5.0 ng mL^−1^	7.6 × 10^−8^ ng mL^−1^	Crop/94.6–102%	253
26	EC	DON	MB@ZIF-8/SA/sDNA/DON(2 ng mL^−1^)/Apt/MCH/cDNA/PEI-rGO/AuNWs/AuE	0.01–100 ng mL^−1^	0.002 ng mL^−1^	Maize flour/81.85–109.4%	253
27	EC	DON		1 × 10^−8^–1 × 10^−4^ mg mL^−1^	6.9 × 10^−9^ mg mL^−1^		254
28	EIS	T-2	Label DNA @AgPdNFs/help DNA/substrate DNA/AuONs@GO/AuE	1 × 10^−2^–1 × 10^4^ pg mL^−1^	6.71 fg mL^−1^	Beer/86.2–96.1%	255
	DPV						
29	EC	DON		0.1–5000 pg mL^−1^	0.0186 pg mL^−1^	Grain/85.67–118.0%	256
30	EIS	T-2	T-2/RecJfApt-sDNA/AuNPs/FeMOF@GO/AuE	5.0 × 10^−1^–5.0 × 10^6^ pg mL^−1^	0.19 pg mL^−1^	Beer/92.5–97.8%	257
	DPV						

#### 3.1.5 Zearalenone

Zearalenone (ZEA) is a resorcyclic acid lactone mycotoxin produced by Fusarium graminearum, culmorum and cerealis. Due to its estrogenic properties, this mycotoxin commonly contaminates cereal crops such as maize, wheat, barley, and oats, posing a risk to both animal and human health.^258^ Corn, sorghum, wheat, rice, barley, oats, pecans, soybeans and sesame are among the cereals contaminated with ZEA. Due to its structural resemblance to estrogens, ZEA has been demonstrated in numerous *in vivo* studies to disrupt hormonal homeostasis.^259^ Mammals experience problems with reproduction and fertility as a result of mycotoxins' strong binding to estrogen receptors.^260^ Cancer is linked to progressive exposure to endocrine-modulatory chemicals. Toxin bioavailability in humans, rats, rabbits, and pigs can exceed 80%, according to a 2014 EFSA assessment.^79^ According to recent studies, ZEA is processed in the liver and causes nephrotoxicity, immunotoxicity, cancer, and hepatotoxicity in animals.

##### 3.1.5.1 Detection of ZEA

For maize ZEA detection, a dual-signal immunoassay activated by ALP has been reported.^258^ The sensitivity may arise from alkaline phosphatase-generated l-ascorbic acid, reducing the K_3_[Fe(CN)_6_] mediator to K_4_[Fe(CN)_6_], whose rapid electron shuttling at the AuNP-modified electrode slashes charge-transfer resistance and produces a large faradaic current. ZEA is captured by its monoclonal antibody *via* multiple non-covalent hydrogen bonds and hydrophobic contacts, localizing ALP-IgG at the interface to ensure specific mediator consumption and signal amplification. The role of K_3_[Fe(CN)_6_] in multicolor creation and electron transfer allowed colorimetric and electrochemical assays to quantify ZEA. The detection limits for colorimetric and electrochemical techniques were 0.04 and 0.08 ng mL^−1^. ZEA recovery from cornmeal samples was 80–120%, with relative standard deviations below 10%. The dual-signal immunoassay demonstrated high sensitivity in detecting ZEA in corn samples. Another work combined NiO with carboxylated multi-walled carbon nanotubes (MWCNT-COOH) to create an electrochemical sensor that does not require biomolecules and can detect ZEA and OTA. For the first time, ZEA and OTA have been simultaneously detected by a NiO-MWCNT (–COOH) electrochemical sensor. Under optimal conditions, ZEA (0.01–10.24 μg mL^−1^) and OTA (0.04–10.24 μg mL^−1^) showed linear responses, with detection limits of 6 and 15 ng mL^−1^, respectively.^261^ For ZEA, Azri *et al.* created an electrochemical aptasensor. A 1,4-phenylene diisocyanate linker and cysteamine hydrochloride were used to bind ZEA to the gold electrode covalently. A shortened ZEA aptamer with a dissociation constant of 13.4 ± 2.1 nM was utilized to develop an aptasensor. Using ferro/ferricyanide as a redox probe, SWV was utilized to examine changes in electron transport. The best measurement of the reaction under suitable experimental conditions was at 0.20 V (*vs.* Ag/AgCl). The competition for the aptamer binding site between the free and immobilized ZEAs was necessary for the signals to occur. Amine-terminated cysteamine/PDIC layers on the Au electrode create a positively charged interface that strongly attracts the [Fe(CN)_6_]^3−/4−^ redox probe. In the competitive assay, the displacement of the negatively charged aptamer–ZEA complex reduces surface blocking, accelerating electron transfer and amplifying the faradaic current. ZEA is covalently tethered *via* urethane bonds (isocyanate–phenol coupling) and its aptamer recognizes it through hydrogen bonding, π–π stacking, and van der Waals interactions between the phenolic/lactone groups and nucleobases. Aptasensor has a detection limit of 0.017 ng mL^−1^ and can detect ZEA in the concentration range of 0.01 to 1000 ng mL^−1^. Cross-reactivity with other ZEA analogs was high, but not with other mycotoxins. After quantifying ZEA in maize grain extract, the aptasensor showed 87–110% recovery.^262^

Zaman *et al.* employed a SPE using a 3D carnation flower-like Tb^3+^/Co_3_O_4_ nanocomposite to detect ZEA in authentic dairy products, juices, and other liquid foods.^261^ The modified electrode was analyzed electrochemically using DPV, EIS, CV, and chronoamperometry. ZEA's proton receptor groups caused its peak current to vary with pH. The maximum signal of the received stream indicated the optimal ZEA signal in PBS at pH 7.0. The improved sensing may derive from the flower-like Tb^3+^/Co_3_O_4_ nanocomposite's exceptional conductivity and enlarged electroactive surface area. Tb^3+^ induced oxygen vacancies slash charge-transfer resistance and catalyze the diffusion-controlled electrocatalytic oxidation of zearalenone. Zearalenone's phenolic –OH and lactone C=O moieties form hydrogen bonds with surface oxide sites and coordinate to Tb^3+^ centers, securing selective adsorption and efficient electron exchange. For ZEA concentration, the sensor exhibited a broad linear range (0.001–500.0 μM) and an accurate detection limit of 0.34 nM. Zhou *et al.* employed a highly sensitive and selective molecularly imprinted electrochemical sensor to detect ZEA using reduced graphene nanoribbons (GNRs) and AuNPs. To synergistically increase the electrochemical signal, oxidized GNRs are created *via* enhanced Hummers' oxidation, then reduced and modified with AuNPs on a GCE using electrodeposition. With a detection limit of 0.34 ng mL^−1^, the sensor's linear detection range for ZEA is 1–500 ng mL^−1^.^263^ A high-sensitivity signal-off electrochemical aptasensor for detecting ZEA traces was developed in another study. The Ce-based MOF and MWCNTs nanocomposite, functionalized with polyethyleneimine (P–Ce-MOF@MWCNTs), served as a sensing platform due to its wide surface area and high electrochemical activity. The aptamer was then attached using Pt@Au NPs, and the signal probe, toluidine blue, was electrodeposited. The enhanced current arises from the synergy of Ce-MOF's large, catalytically active Ce–O porous network and MWCNT's exceptional conductivity, which together slash charge-transfer resistance and accelerate electron hopping of the toluidine blue tag to the GCE. Zearalenone's phenolic –OH and lactone C=O functionalities form hydrogen bonds with PEI amines and coordinate to Ce^3+^ centers, while its aromatic core π–π stacks with CNT walls, immobilizing the analyte close to the transducer for efficient sensing. Under optimal conditions, the aptasensor displayed a linear relationship for ZEA in the concentration range of 5.0 × 10^−5^ to 50.0 ng mL^−1^ 1.0 × 10^−5^ ng mL^−1^ was the detection limit (LOD, S/N = 3), and 2.9 × 10^−5^ ng mL^−1^ was the quantitation limit (LOQ, S/N = 10).^264^

**Fig. 9:**
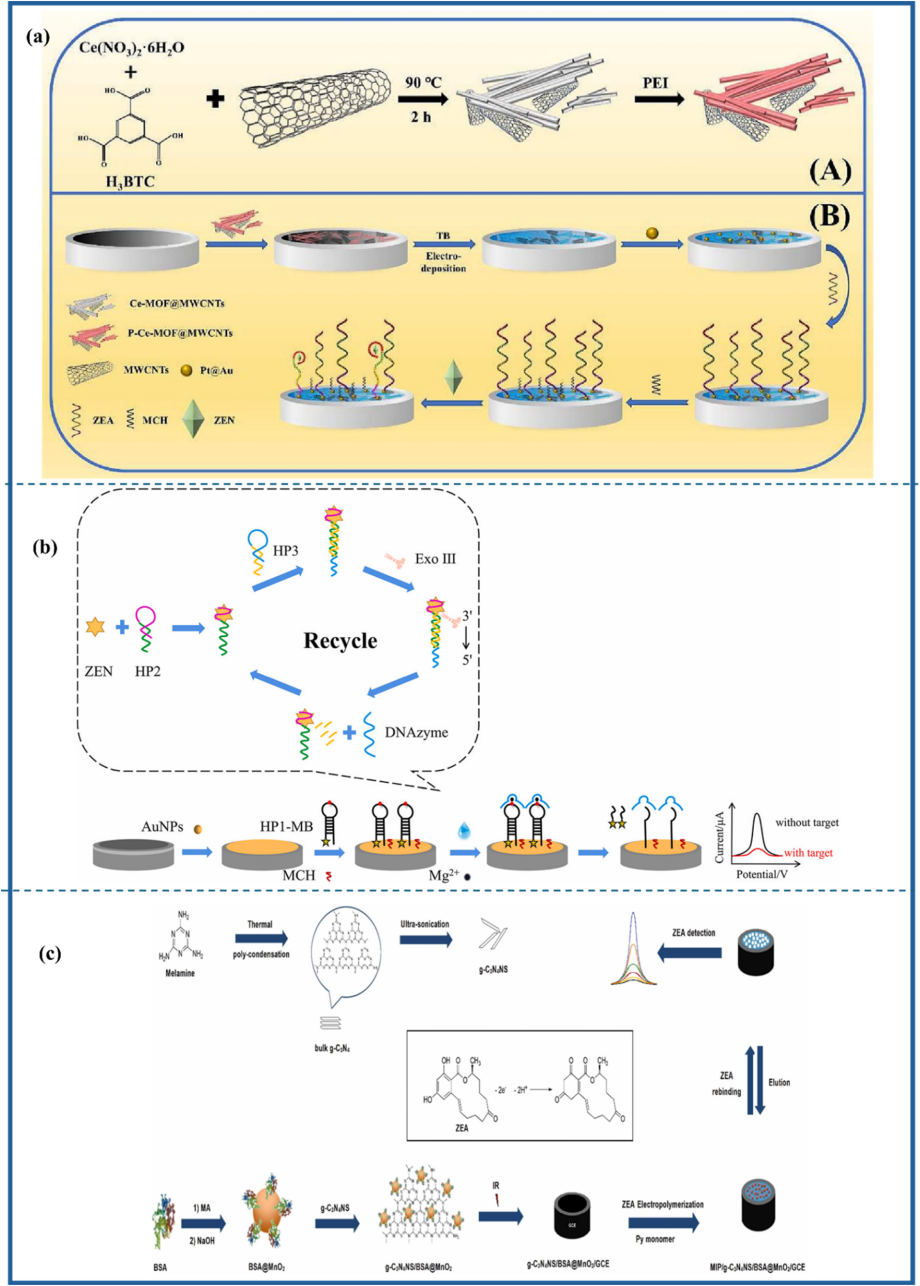
(a) Schematic diagrams of the synthesis of P–Ce-MOF@MWCNTs and the stepwise construction of the electrochemical aptasensor for the detection of ZEA, reproduced from ref. 264 with permission from Elsevier, copyright 2023. (b) Schematics for the Detection of zearalenone by electrochemical aptasensor based on enzyme-assisted target recycling and DNAzyme release strategy, reproduced from ref. 265 with permission from Elsevier, copyright 2025. (c) Schematic preparation of g-C_3_N_4_NS/BSA@MnO_2_ nanocomposite and imprinted electrode for ZEA detection, reproduced from ref. 266 with permission from Elsevier, 2024.

Recent advances in electrochemical biosensing for zearalenone detection are comprehensively illustrated in Fig. 9, highlighting three innovative sensor designs that employ distinct signal amplification and recognition strategies. The first approach (Fig. 9a) demonstrates an aptasensor utilizing a phosphorus-doped cerium metal–organic framework (P–Ce-MOF) decorated with multi-walled carbon nanotubes (MWCNTs), which synergistically enhances electrical conductivity and provides abundant binding sites for aptamer immobilization, enabling highly sensitive quantification of ZEA. A more sophisticated enzyme-assisted mechanism is shown in Fig. 9b, where target recycling and DNAzyme release strategies are combined to achieve exponential signal amplification, significantly lowering the detection limit through catalytic hairpin assembly and enzymatic signal enhancement. The most autonomous design is presented in Fig. 9c, featuring a self-reporting molecularly imprinted sensor based on copper hexacyanoferrate (CuHCF) modified with reduced graphene nanoribbons-reduced graphene oxide. Here, the redox-active CuHCF serves as both a recognition element and an intrinsic signal reporter, eliminating the need for external probes. These four approaches collectively demonstrate the field's progression from conventional receptor-based detection to intelligent systems that integrate nanozymes, molecular imprinting, and self-signaling materials, all while addressing the critical need for rapid, sensitive, and specific ZEA monitoring in food safety applications. The strategic combination of advanced nanomaterials with clever molecular recognition principles in these designs provides a blueprint for next-generation mycotoxin sensors that strike a balance between sensitivity, selectivity, and practical applicability.

Liu *et al.* developed an electrochemical sensor for ZEA by utilizing a carefully selected mimic peptide from a screened library, validated through molecular modeling and electrochemical methods. The construction of the mimic peptide library involved amino acid mutations, dynamic simulations, molecular docking, and structural analyses. The incorporation of gold nanoparticles (AuNPs) and reduced carboxylated graphene oxide significantly enhanced the electrical signal. The sensor demonstrated a linear detection range from 0.01 to 10 ng mL^−1^, achieving an impressive limit of detection of 0.91 pg mL^−1^ under optimal conditions. The commercial ELISA kits varied from 82% to 108.8%, while the grain sample recovery rate ranged from 84% to 105.3%.^267^ ZEA in rice flour, maize powder, and morning cereal can be measured using a simple electrochemical nanohybrid sensor. A magnetic Fe_3_O_4_-GO-modified electrode and a Cu-based MOF are key features of this layer-by-layer-built sensor. The synergistic coupling of highly porous, electrocatalytic Cu-MOF with conductive Fe_3_O_4_-GO maximizes the electroactive surface area and minimizes charge-transfer resistance, thereby accelerating electron transfer. ZEA is preconcentrated and recognized through hydrogen bonding between its phenolic –OH and lactone C=O groups and the carboxylates of MOF/GO, π–π stacking of its aromatic ring on graphene oxide, and coordination of its carbonyl oxygens to Cu centers. The well-designed sensor is efficient, has a wide surface area, and has exceptional electron transport. Under ideal conditions, the limit of detection for ZEA electrochemical detection was 23.14 ng mL^−1^, with a range of 159.2 to 2865.2 ng mL^−1^.^268^

Wang *et al.* developed CuO@GO nanocomposites as an electrochemical sensor for detecting ZEA mycotoxins in food samples. The toxin's aromatic core π–π stacks with graphene, its phenolic –OH hydrogen bonds to GO oxygen functionalities, and its carbonyl oxygen coordinates to Cu^2+^ sites, leading to selective adsorption and rapid electron exchange. The electrochemical characterization of ZEA on CuO@GO/GCE, using CV and DPV techniques, demonstrated a linear detection range of 10 to 150 ng mL^−1^, a sensitivity of 0.4895 μA ng^−1^ mL^−1^, and a detection limit of 0.012 ng mL^−1^.^269^ Additionally, an electrochemical aptamer sensing method utilizing PEI@Ti-MOF@Ti_3_C_2_T_*x*_-MXene was developed for the identification of ZEA in food products. The synthesis of NH_2_-MIL-125 involved the use of tetra-butyl titanate as the metal center and 2-amino terephthalic acid as the organic ligand. Simultaneous hybridization of Ti_3_C_2_T_*x*_-MXene resulted in the formation of the Ti-MOF@Ti_3_C_2_T_*x*_-MXene composite material. The composites were utilized to develop a sensing platform on gold electrodes, following their functionalization with PEI and subsequent covalent bonding. The sensor achieved a LOD of 1.64 fg mL^−1^. Furthermore, it demonstrated remarkable stability, reproducibility, and selectivity in identifying ZEA in beer and cornmeal samples.^270^Table 5 presents the detection of ZEA using different methods along with their LODs.

**Table 5:** Detection of ZEA on various sensing platforms along with their analytical parameters

S. No.	Method	Analyte	Electrode modifier	Linear range	LOD	Sample/recovery	Ref.
1	EC	ZEA	MNPs-aptamer/TRFLNPs-cDNA	0.001–10 ng mL^−1^	0.21 pg mL^−1^	Maize/80.76–119.66%	271
						Wheat/90.04–114.75%	
2	TR-aptasensor	ZEA	—	0.001–100 ng mL^−1^	0.51 pg mL^−1^	Maize/94.4–98.0%	243
3	EC	ZEA	ZEA/CGO-ZBA/BSA/PCS/AuNPs/CS@AB-MWCNTs/GCE	10 fg mL^−1^–10 ng mL^−1^	3.64 fg mL^−1^	Corn oil/92.81–99.58%	272
						Corn flour/101.12–111.5%	
4	EC	ZEA	Au/Cys/PDIC/ZEA/Apt	0.01–1000 ng mL^−1^	0.017 ng mL^−1^	Maize grain/87–110%	262
5	DPV	ZEA	Thi/GO/MCH/Apt/AuNPs/Nafion/p-PtNTs/AuE	0.5 pg mL^−1^–0.5 μg mL^−1^	0.17 pg mL^−1^	Maize/97.3–106%	273
6	Colorimetric aptasensor	ZEA	—	10–250 ng mL^−1^	10 ng mL^−1^	Corn oil/101.24–104.92%	274
						Corn/92.34–115.59%	
7	Colorimetric aptasensor	ZEA	—	4–128 ng mL^−1^	4 ng mL^−1^	Maize powder/96.44–99.78%	275
						Mouse feed/95.99–103.73%	
8	Colorimetric aptasensor	ZEA	—	20–8000 ng mL^−1^	10 ng L^−1^	Human serum/95–103%	276
9	Aptamer-based lateral flow test strip	ZEA	—	5–200 ng mL^−1^	20 ng mL^−1^	Corn/93.4–114.2%	277
10	EC	ZEA	GCE/rMoS_2_-Au/Aps/BSA/L-CPs	0.001–10 ng mL^−1^	0.5 pg mL^−1^	Maize/95.9–105.2%	278
11	DPV	ZEA	Anti-ZEA/MCM-41-Fe_2_O_3_	1.88–45 ng mL^−1^	0.57 ng mL^−1^	Amaranthus cruentus seeds	279
12	HPLC	ZEA	—	1.29–16.88 ng mL^−1^	1.29 ng mL^−1^	Field corn	280
13	CV	ZEA	Apt/Pt@AuNPs/TB/PEI-MoS_2_-MWCNTs/Au	0.5 pg mL^−1^–50 ng mL^−1^	0.17 pg mL^−1^	Beer/85.3–100.2%	281
14	EC	ZEA	Cu@L-GA/NPs/Apt/PANI-AuNPs/GCE	1 fg mL^−1^–100 ng mL^−1^	0.45 fg mL^−1^	Beer/92–105%	282
15	DPV	ZEN	ZEN-BSA/EDC-NHS/SPCE	8–32 ng mL^−1^	2 ng mL^−1^	Beer/89–97%	283
						Wine/91.5–96.5%	
16	CV	ZEN	CoPC/SPCE	25–400 ng cm^3^	0.15 ng cm^3^	Corn/94.1–103.9%	284
	DPV						
17	DPV	ZEN	—	100–600 ng mL^−1^	29.47 ng mL^−1^	Corn flour, corn starch and Malt	285
18	CV	ZEN	SWCNTs/SPCE	2.5 × 10^−8^–1× 10^−6^ M	5 × 10^−9^ M	Cornflakes/98.6–103.3%	286
	DPV						
	EIS						
19	DPV	ZEN	PAN/Glu/PGE	5–30 and 60–100 nM	1.66 nM	Dairy juices	287
	SWV						
	EIS						
20	EC	ZEN	ZENxpAb/AuNPs/MWCNTs/PEI/SPCE	0.1 pg mL^−1^–0.1 ng mL^−1^	0.15 pg mL^−1^	Maize/100.4–104.6%	288
21	CV	ZEN	ZEN/MCH/DNAS2-Apt/AuNPs/Fe_3_O_4_NRs/rGO/AuE	0.5 pg mL^−1^–0.5 ng mL^−1^	0.105 pg mL^−1^	Maize/91.6–104.4%	289
	DPV						
	EIS						
22	EIS	ZEN	MCH/DNAH1/CoSe_2_/AuNRs/AuE	10 fg mL^−1^–10.0 ng mL^−1^	1.37 fg mL^−1^	Maize/93.6–103.4%	290
23	EC	ZEN	Ab/EDC-NHS/LA/Au	0.010–10 nM	1.9 pg mL^−1^	—	291
24	CV	ZEN	ZEN/BSA/biotin-Ab/streptavidin/GCE	10 pg mL^−1^–3 ng mL^−1^ and 3–12 ng mL^−1^	3.7 pg mL^−1^	Urine/98.5–101%	292
	EIS						
25	EC	ZEN	3D CF-L TB^3+^/Co_3_O_4_ NC/SPE	0.001–500.0 μM	0.34 nM	—	293
26	CV	ZEN	MIP/AuNPs/rGNRs/GCE	1–500 ng mL^−1^	0.34 ng mL^−1^	Maize	263
	DPV						
27	CV	ZEN	ZEN/MCH/L3/AuNPs/rGO–COOH–apt/GCE	0.01 ng mL^−1^–10 ng mL^−1^	0.91 pg mL^−1^	Corn/98.0–101.3%	267
	EIS					Wheat/90.0–108.8%	
	DPV					Oat/82.0–106.0%	
						Bran/94.0–98.75%	
28	DPV	ZEN	GCE/P–Ce-MOF@MWCNTs/TB/Pt@Au/ZEA	50.0 fg mL^−1^–50.0 ng mL^−1^	12.0 fg mL^−1^	*Semen coicis*/97.06–102.9%	264
29	CV	ZEN	CuO@GO/GCE	10–150 ng mL^−1^	0.012 ng mL^−1^	Milk/84.4–97.0%	269
	DPV						
30	CV	ZEN	AuNP-SE/ZEN	10 ng kg^−1^–10 mg kg^−1^	1.1 ng kg^−1^	Maize and oats	294
	DPV						
31	SERS	ZEN	Substrate (MSN-Rh6G-AuNPs)	3–200 ng mL^−1^	0.22 μg L^−1^	Corn	295
32	SERS	ZEN	—	10–1000 μg kg^−1^	3.6 μg kg^−1^	Maize/86.06–111.23%	296
33	Fluorescence based aptasensor	ZEN	—	0.01–100 ng mL^−1^	1.2 pg mL^−1^	Corn and oat flour	297
34	EC	ZEN	VMSF/ITO	0.001–1000 ng mL^−1^	1.2 × 10^−6^ ng mL^−1^	Maize/97.1–103%	298
						Chestnut/96.0–105%	
35	ECL	ZEN	ZEN/MB/ZBA/BSA/cDNA/CS/NGQDs-Ru@SiO_2_/GCE	1.0 × 10^−15^–5.0 × 10^−8^ g mL^−1^	0.85 fg mL^−1^	Maize flour/93.2–102.8%	299
36	CV	ZEN	MIP/AuSPE	1 × 10^−4^–1 × 10^2^ ng mL^−1^	3.4 × 10^−5^ ng mL^−1^	Maize/92–108%	300
	EIS						
37	EC	ZEN	B-ZIF@CNP/Ag/Ab/Anti-IgG-Bio/A-GOx-modified AuE	0.87–1058.5 pg mL^−1^	0.87 pg mL^−1^	Wheat, peanuts and feed/76.4–108.2%	301
38	DPV	ZEN	PEI-rGO/Pt@AuNRs	1–1 × 10^6^ pg mL^−1^	0.02 pg mL^−1^	—	302
39	DPV	ZEN	ZEN/BSA/Apt/PEI@T@M/AuE	1 × 10^−5^–1 × 10^−11^ mg mL^−1^	1.64 fg mL^−1^	Beer/87.7–105.2%	270 and 303
	CV					Corn starch/98.2–101.2%	
	EIS						
40	EIS	ZEN	SPCE/CS-CNT-Pd/EDC-NHS/ZEN/-MAb/ZEN	0.25–16 ng mL^−1^	0.25 ng mL^−1^	Corn/86.5–98.6%	303
	CV						
41	OMPD	ZEN	GPE/CNH/GNP	10–1000 μg kg^−1^	4.40 μg kg^−1^	Corn/91–105.2%	304
42	CV	ZEN	MIP/g-C_3_N_4_NS/BSA@MnO_2_/GCE	1.0–10.0 ng L^−1^	0.25 ng mL^−1^	Rice	305
	EIS						
43	OWLS immunosensor	ZON	APTS/SA/EDC-NHS	0.01–1 pg mL^−1^	0.002 pg mL^−1^	Maize	306
44	FRET	ZEN	(Aptamer-SAF-UNCP-FGO) probe	0.005–100 ng mL^−1^	0.0018 ng mL^−1^	Maize/92.30–110.68%	307
45	EIS	ZEN	ZEN/BSA/Apt/CuBi-BPDC/AE	1 fg mL^−1^–10 ng mL^−1^	0.19 fg mL^−1^	Milk/91.2–109.9%	308
	DPV			0 fg mL^−1^–1 × 10^9^ fg mL^−1^	0.73 fg mL^−1^	Rice/92.0–109.9%	
46	DPASV	ZEA	Cu-MOF/Fe_3_O_4_-GO/GCE	159.2–2865.2 ng mL^−1^	23.14 ng mL^−1^	Maize powder/93.3–97.7%	268
						Rice flour/94.9–113.3%	
47	CV	ZEA	MIP/CuHCF/rGNR-rGO/GCE	0.25–500 ng mL^−1^	0.09 ng mL^−1^	Corn meal/97.30–102.18%	309
	DPV						
48	Amperometry	ZEA	Bi_2_S_3_@CNF/GCE	0.125–375.5 μM	0.61 μM	Wheat and oats/98.9–99.15%	310

#### 3.1.6 Patulin (PAT)

PAT is an electrophilic α, β-unsaturated γ-lactone, which interacts with ascorbic acid, free amino groups, sulfur dioxide, and sulfhydryl groups. PAT has been reported to have gastrointestinal, immunotoxic, genotoxic, and mutagenic effects, which can damage vital organs and systems.^311^ Therefore, a maximum acceptable concentration of 50 μg L^−1^ PAT in apple juice and cider has been set by China, the US, the EU, and other countries.^312^ Patulin (4-hydroxy-4*H*-furo[3,2-*c*]pyran-2(6*H*)-one, PAT), a mycotoxin found in various foods, is produced by a wide range of fungi.^313^ Under optimal temperature and humidity conditions, Aspergillus and Penicillium species synthesize this secondary metabolite. In 1941, Glister identified, isolated, and designated PAT. Because PAT inhibits both Gram-positive and Gram-negative bacteria (*e.g.*, *Shigella* spp., *Salmonella typhi*, and *E. coli*), it was the focus of earlier clinical trials.^314^ Research in the 1960s demonstrated that PAT was detrimental to bacterial cells and animal models. Consequently, PAT is infrequently utilized as a therapeutic agent in clinical environments, with investigations concentrating on its toxicity and contamination of food and feed.^315^

Pears, blueberries, cherries, peaches, plums, mulberries, rotten apples, and pears all contain polyphenolic acids or polyphenols. Due to the higher concentration of PAT in apples, the majority of individuals obtain their PAT from apples and apple-derived products. Recent assessments of PAT by other researchers have been exceptional. These evaluations focus on PAT domains, including synthesis, distribution, and others. An updated evaluation is necessary to incorporate the latest insights from significant studies on the toxicity and detoxification of PAT. This study emphasizes the benefits and potential of the biological approach, highlights the lack of interaction between intestinal microbiota and PAT, and enhances the detoxification model through a thorough analysis of PAT. Regarding PAT toxicity, our findings also included vitamin, trace metal, and probiotic ingestion. We anticipate that compiling perspectives may help mitigate PAT contamination in food and stimulate future studies. Despite clear correlations between PAT contamination numbers and food, epidemiological data about population-level PAT exposure are limited. These investigations did not consider PAT bio-acceptability across various dietary media. Furthermore, fruits and vegetables may exhibit low PAT uptake, thereby reducing mealtime exposure to levels below the provisional maximum allowable daily dose. PAT contamination levels in fruit products exceed permitted limits in several locations, posing a health risk to residents. Future studies should investigate the co-occurrence of PAT with other mycotoxins in food and their combined and synergistic health effects, even if PAT alone poses a negligible risk.

##### 3.1.6.1 Detection of PAT

The successful fabrication of a rGO/SnO_2_ composite for the electrochemical detection of the fungal contaminant PAT by Shukla *et al.* eliminated the need for specific antibodies or chemical or biological sensors. The resulting rGO/SnO_2_ composite demonstrated exceptional performance in the direct assessment of PAT levels in contaminated apple juice samples, exhibiting excellent electrochemical properties. The high DPV current originates from the synergistic coupling of conductive rGO and electrocatalytic SnO_2_, which minimizes charge-transfer resistance and facilitates the rapid two-electron oxidation of patulin. PAT adsorbs onto the composite through π–π stacking of its furan-lactone core with the graphene lattice, hydrogen bonding of its hydroxyl and carbonyl groups to GO's oxygen functionalities, and coordination of its carbonyl oxygen to Sn^4+^ centers. With a detection limit of 0.6635 nM, the rGO/SnO_2_ composite electrode's DPV response showed a linear relationship with PAT concentration over the 50–600 nM range. The sensor electrode showed remarkable selectivity, consistent repeatability, and sensitivity.^316^ The harmful properties of increased PAT concentrations underscore the importance of developing new detection methods, which are crucial for advancing research and creating revolutionary control technologies. A new sensor for the SWV method of PAT toxin detection was developed, utilizing a GCE enhanced by an ionic liquid-based MIP and Fe_3_O_4_/GO composite. A linear range of 0.001 nM to 250.0 nM was established under optimized conditions, with a LOQ of 0.001 nM and a LOD of 3.33 × 10^−4^ nM.^317^ To achieve ultra-sensitive and selective PAT detection, a distinct study presented a novel method for creating a molecularly imprinted electrochemical sensor. Initially, N-GQDs were integrated with Au@Cu-MOF to enhance the surface of a GCE. Electro-polymerization was then used to create a layer of MIP on the Au@Cu-MOF/N-GQDs/GCE. The dense AuNPs, conductive N-GQDs, and abundant Cu(ii)/Cu(i) redox centers slash charge-transfer resistance and accelerate the −0.11 V Cu–MOF redox signal. Patulin then selectively binds to imprinted cavities and gets locked *via* hydrogen bonds (phenolic –OH to polymer/N-GQD oxides), π–π stacking of its furan-lactone ring on graphitic domains, and coordination of its carbonyl oxygens to Cu^2+^ nodes, gating electron flow to modulate the current. With an unusually low detection limit of 0.0007 ng mL^−1^, the developed MIP sensor showed a wide linear range from 0.001 to 70.0 ng mL^−1^.^318^

**Fig. 10:**
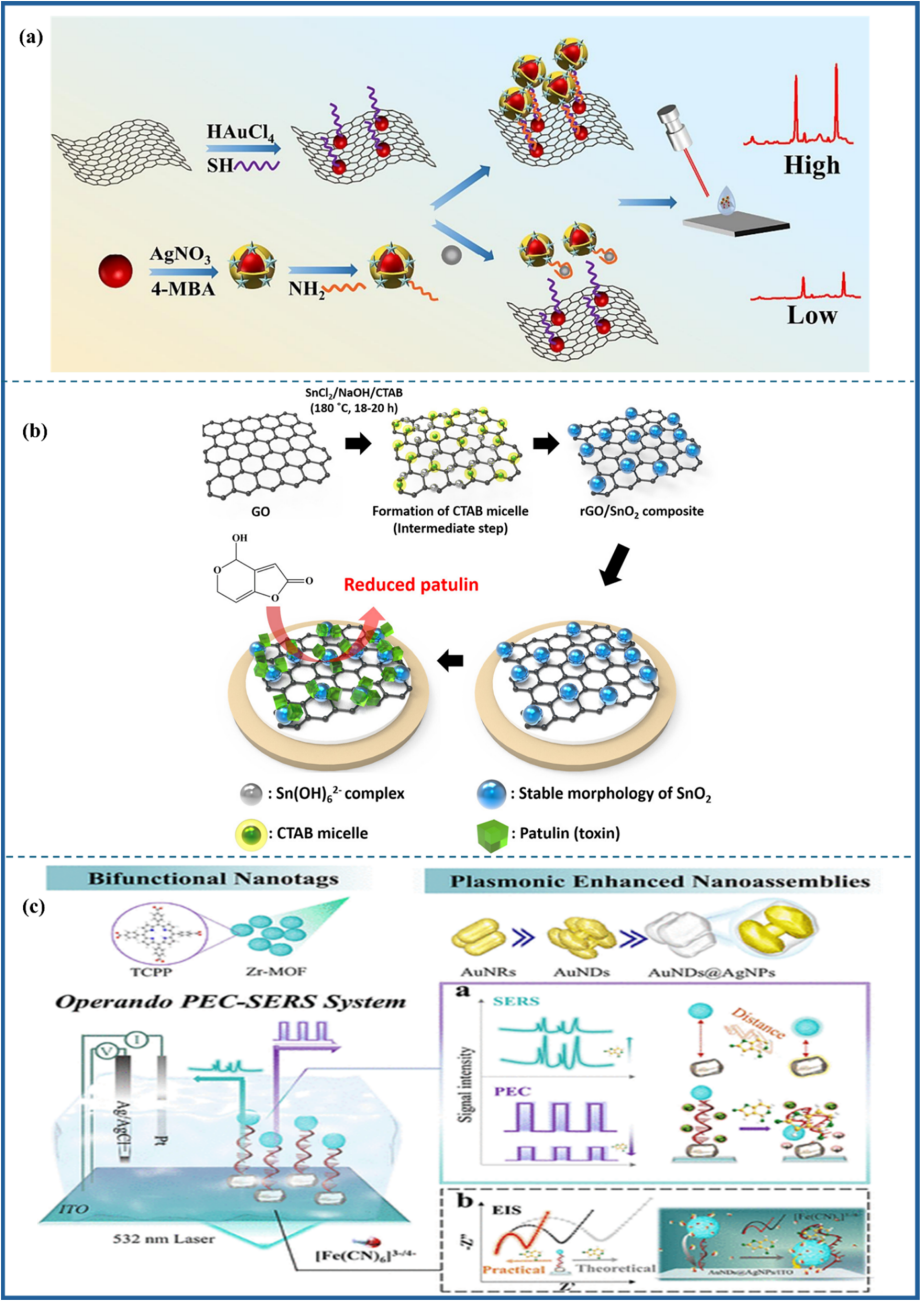
(a) Schematic diagram of the film-like SERS aptasensor based on GO@Au nanosheets for the detection of PAT, reproduced from ref. 319 with permission from Elsevier, copyright 2024. (b) Schematic representation of the fabrication of the rGO/SnO_2_ nanocomposite and its electrochemical sensing of PAT, reproduced from ref. 316 with permission fom Elsevier, copyright 2020. (c) Schematic illustration of the *operando* PEC-SERS biosensor for PAT detection: (a) analysis of biosensing mechanism *via* the *operando* PEC-SERS method and (b) analysis of biosensing mechanism *via* the electrochemical characterization method, reprinted from ref. 320 with permission from American Chemical Society, copyright 2025.

The detection of patulin (PAT), a hazardous mycotoxin commonly found in fruit products, is demonstrated through three advanced sensing platforms in Fig. 10, each employing distinct detection mechanisms and nanomaterial engineering strategies. Fig. 10a presents an innovative film-like surface-enhanced Raman spectroscopy (SERS) aptasensor utilizing graphene oxide-gold nanosheet composites (GO@Au), where the large surface area and plasmonic properties enable ultrasensitive PAT detection through aptamer-mediated recognition and significant Raman signal enhancement. Transitioning to electrochemical detection, Fig. 10b illustrates a bioreceptor-free sensor based on a reduced graphene oxide-tin oxide nanocomposite (rGO/SnO_2_), which leverages the synergistic effects of excellent electrical conductivity and metal oxide catalytic activity for rapid PAT quantification without the need for biological recognition elements. The most sophisticated approach is illustrated in Fig. 10c, featuring an *operando* photoelectrochemical-SERS (PEC-SERS) biosensor that combines *in situ* electrochemical characterization with Raman spectroscopy, enabling the simultaneous investigation of interfacial charge transfer mechanisms and highly sensitive PAT detection through dual-mode signal verification. These three platforms collectively demonstrate the evolution from single-mode detection to multifunctional analytical systems, highlighting how strategic nanomaterial design and multimodal sensing can address the challenges of PAT monitoring in complex food matrices. The progression from aptamer-based recognition to bioreceptor-free detection and, ultimately, to *operando* mechanistic analysis reflects the field's shift toward more robust, informative, and practical sensing solutions for food safety applications.

Food PAT poses a significant risk to human health, making the development of a precise and sensitive detection method essential. In this context, Jiang *et al.* developed an electrochemical assay for PAT detection by synthesizing aptamers on a carboxylated hierarchically porous carbon (HPC–COOH)-modified glassy carbon electrode, achieving high sensitivity in their approach. Due to its wide surface area, the HPC-COOH is a unique substrate for immobilizing aptamers, thereby enhancing target PAT capture and identification. The hierarchically porous, carboxylated carbon network of HPC-COOH slashes charge-transfer resistance and boosts Fe(CN)_6_^3−/4−^ electron shuttling at the GCE. At the same time, amino-terminated aptamers are covalently tethered *via* amide bonds to –COOH sites, and patulin's phenolic –OH and lactone C=O groups engage the aptamer through hydrogen bonding and π–π stacking, gating mediator access, and modulating the faradaic current. With a low detection limit of 0.25 ng L^−1^, the modified aptasensor was able to detect PAT at concentrations ranging from 0.5 to 5.0 × 10^6^ ng L^−1^.^321^ A RuMOF@hydrogel and carbonized polymer dots generated from banana peels were used to create a multimodal PAT aptasensor carefully. The RuMOF@hydrogel-modified magnetic electrode exhibited excellent anodic and cathodic electrochemiluminescence (ECL) emission and stability. The synergistic conductivity and dual-potential electrochemiluminescence of RuMOF@hydrogel and BPPDs@SiO_2_ lead to improved electron transport and amplify the anodic ECL response; patulin binds its aptamer *via* hydrogen bonding and π–π stacking between its phenolic –OH and lactone C=O groups and the nucleobases, triggering a hybridization chain reaction and magnetic enrichment that concentrate signal probes at the electrode interface. The detection limit for ECL was 2.5 fg mL^−1^, resulting in reasonable specificity, stability, and recovery.^322^ By modifying electrodes with polyethyleneimine-reduced graphene oxide composites (Pt@AuNRs/Fe-MOFs/PEI-rGO), iron-based MOFs, and gold-platinum core–shell nanorods, a DNA walking machine improves signal quality. For PAT detection, Zr-based MOFs-labeled oligonucleotides loaded with MB (MB@Zr-MOFs-cDNA) serve as signal probes, providing a sensitive electrochemical aptasensor. The Pt@AuNRs/Fe-MOFs/PEI-rGO modified gold electrode shows no change after 60 scans. Its catalytic performance is 250% better than that of a gold electrode. A computed apparent electron transfer rate constant of 1.64 s^−1^ confirms high conductivity. With a detection limit of 4.14 × 10^−5^ ng mL^−1^, the aptasensor with this design has an excellent sensitivity range of 5.0 × 10^−5^ to 5.0 × 10^−1^ ng mL^−1^.^323^

Guo *et al.* introduced an innovative assay for detecting PAT contamination in food. Their approach was based on the secondary structure of the aptamer, resulting in a unique truncation strategy. By eliminating the stem-loop structure, they optimized P-30, a truncated version of the PAT aptamer. In this assay, the gold nanocluster-modified aptamer functioned as the fluorescent probe, while BSA-directed MnO_2_ nanoflakes served as the receptor. The intensification of fluorescence signal in this DNase I-assisted aptasensor arises from the efficient Förster resonance energy transfer (FRET) between Au nanocluster-labeled aptamers and MnO_2_ nanoflakes, where patulin binding induces aptamer desorption and fluorescence recovery. Specific recognition is driven by hydrogen bonding (*e.g.*, between PAT's hydroxyl and carbonyl groups and aptamer bases like A3, T5, C4) and π–π stacking interactions (*e.g.*, between PAT's furan ring and adenine residues such as A25), ensuring selective target capture and signal amplification. The assay was effective in apple and grape juice samples, with a detection limit of 8.5 ng L^−1^.^324^ A GCE sensor was carefully designed for quick and sensitive detection of PAT. Anti-patulin-BSA IgG was produced by rabbits and immobilized on a graphene oxide/gold nanocomposite. Anti-PAT-BSA IgG on the GCE sensor can collect mycotoxin PAT in samples. IgG and PAT interaction reduced electron transfer resistance and IgG's spatial obstruction effect on the GCE sensor. PAT detection was made possible by recent modifications to the immobilized anti-PAT-BSA IgG GCE sensor, which showed a linear relationship with PAT concentration. With a detection limit of 5 μg L^−1^, the immuno-electrochemical GCE sensor can detect PAT in under a minute.^325^ In traditional Chinese medicine, PAT produced from Crataegus pinnatifida Bge and Semen Coicis can be rapidly detected electrochemically using a ratiometric aptasensor that utilizes NH_2_-MIL-101@MB and an innovative catalytic MnCo@C nanoprobe in conjunction with a straightforward and effective multi-amplification technique. NH_2_-MIL-101@MB's electrode surface modification provided a baseline electrical signal, improving sensor dependability. Redox processes and electron transfer efficiency increased by a new enzyme-mimetic MnCo@C. The detection limit of the PAT sensor is 0.040 fg mL^−1^ (S/N = 3), and its linear range is 1.00 × 10^−4^ to 1.00 × 10^1^ pg mL^−1^. With recovery rates of 90.6–106.3% and 90.2–103.1%, respectively, and RSD values of 1.21–4.70% and 3.18–5.00%, the aptasensor detected PAT in Crataegus pinnatifida Bge and Semen Coicis.^326^Table 6 enlists the sensing parameters of PAT as determined by various investigators.

**Table 6:** Sensing parameters of PAT as determined by various analytical techniques

S. No.	Method	Analyte	Electrode modifier	Linear range	LOD	Sample/recovery	Ref.
1	EC impedimetric aptasensor	PAT	—	1–25 ng mL^−1^	1.25 ng mL^−1^	Apple juice/92.5–96%	327
2	DPV	PAT	rGO/SnO_2_/GCE	50–600 nM	0.6635 nM	Apple juice/74.33–99.26%	316
3	EC	PAT	MIP/Au@Cu-MOF/N-GQDs/GCE	0.001–70 ng mL^−1^	0.0007 ng mL^−1^	Apple juice/97.6–99.4%	318
4	EC	PAT	MIP/thionine-PtNP-NGE/GCE	0.002–2 ng mL^−1^	0.001 ng mL^−1^	Apple juice/99.8–113.0%	328
						Grape juice/95.4–104.8%	
5	EC	PAT	PAT/aptamer-AuNP-BP NSs/GCE	0.1–10 μM	0.03 nM	Apple juice/96.2–104%	329
6	DPV	PAT	PAT/MCH/Apt/DpAu/ZnONRs-CS/AuE	50 ng mL^−1^–0.5 pg mL^−^	0.27 pg mL^−1^	Apple juice/95.6–104.8%	330
7	CE	PAT	—	1–100 μg kg^−1^	0.3 μg kg^−1^	Apple juice/94–98%	331
8	HPLC-DAD	PAT	—	5 ng mL^−1^–10 μg mL^−1^	1.5 μg kg^−1^	Strawberries/53.1–103.6%	332
9	UPLC-MS/MS	PAT	—	42–757 μg kg^−1^	50 μg L^−1^	Apple/76–84%	333
				42–1298 μg kg^−1^	12.5 μg kg^−1^	Apple juice/102–108%	
10	EC	PAT	MIP/Fe_3_O_4_/GO/GCE	0.001 nM-250.0 nM	3.33 × 10^−4^ nM	Apple juice	317
	SWV						
11	EC	PAT	Anti-patulin-BSA/IgG/GCE	5–200 μg L^−1^	5 μg L^−1^	—	325
12	EIS	PAT	Fe_3_O_4_NPs/rGO/PAT/TDNs-Apt/AuE	5 × 10^−8^–5 × 10^−1^ μg L^−1^	30.4 fg mL^−1^	Apple juice/96.9–105%	334
	DPV						
13	EC	PAT	—	1–25 μM	3.56 ng mL^−1^	Apple juice/94.4%	335
14	CV	PAT	MIP/PtPd-NPC/GCE	0.01–10 μg L^−1^	7.5 × 10^−3^ μg L^−1^	Juice (apple & grape)/94–99.8%	336
	EIS						
15	SPES	PAT	Aptamer/G-Bi_2_S_3_/ITO	5.0–500 nM	0.28 nM	Apple juice/97.7–104.8%	337
16	DPV	PAT	BSA/Apt/HPC-COOH/GCE	0.5–5.0 × 10^6^ ng L^−1^	0.25 ng L^−1^	Apple juice and haw juice/86.2–105.4%	321
17	CV	PAT	cDNA/aptamer/AuNPs-BPNS/GCE	0.0154–15.4 ng mL^−1^	0.043 ng mL^−1^	Apple, pear, and tomato/95.0–106.7%	338
	SWV						
18	EIS	PAT	Patulin/pAb-patulin/GO/GCE	0.01–10 ng mL^−1^	9.8 pg mL^−1^	Apple juice/86%	339
19	EC	PAT	CdTe QDs/Au NRs	50 fg mL^−1^–500 ng mL^−1^	30 fg mL^−1^	Apple	340
	PEC						
20	DPV	PAT	MCH/PAT-aptamer & AuNPs-BSA/GCE	0.01 × 10^−7^–0.10 μg mL^−1^	—	Apple juice/98.20–102.70%	341
21	DPV	PAT	EDC/NHS	In buffer & apple juice medium (1–10^4^ pg mL^−1^)	In buffer (0.18 pg mL^−1^) & In apple juice (0.47 pg mL^−1^)	Apple juice/91.24–93.47%	342
22	Amperometry	PAT	Ni–NiO-streptavidin-BSA-aptamer-GCE	10–10^6^ fg mL^−1^	1.65 fg mL^−1^	Apple juice/more than 85%	343
23	EC (EIS, CV & DPV)	PAT	AgPdNPs/c-DNA/reaction solution/MCH/DNAmix/AuNFs/g-C_3_N_4_/AuE	5 × 10^−6^–50 μg L^−1^	0.92 fg mL^−1^	Apple juice/95.5–110%	344
24	Dual-EATR	PAT	AuNPs/FeMOF-PEI-GO/AuE	5 × 10^−7^–5 ng mL^−1^	0.217 fg mL^−1^	Apple juice/91.0–103%	345
25	SPR	PAT	—	0.5–750 nM	0.011 nM	Apple juice/98.24–98.32%	346
26	SERS	PAT	MIP-ir-Au/PDMS/AAO	5 × 10^−10^–5 × 10^−6^ M	8.5 × 10^−11^ M	Apple juice/96.43–112.83%	347
27	EC	PAT	NH_2_-MIL-101@MB	1.00 × 10^−4^–1.00 × 10^1^ pg mL^−1^	0.040 fg mL^−1^	Crataegus pinnatifida *Bge*/90.6–106.3%	326
						*Semen Coicis*/90.2–103.1%	
28	FRET	PAT	—	0.01–100 ng mL^−1^	0.003 ng mL^−1^	Apple juice/93.33–105.21%	348
29	FRET	PAT	—	15–35 μg L^−1^	6 ng L^−1^	Apple juice/94.7–109%	349
30	MIP-SERS	PAT	—	7.00 × 10^−12^–5.00 × 10^−8^ M	5.37 × 10^−12^ M	Fruit products/96–108%	350
31	Fluorescence-apta assay	PAT	—	5–600 ng mL^−1^	0.13 μg L^−1^	Apple juice/96–98%	351
32	SWV	PAT	Pep&Apta/AuNPs/SPE	0.1 fM–10 nM	0.03 fM	Apple and orange juices/99.3–101.2%	352
	DPV						
33	ECL	PAT	Apt/PTCA/Tb MOG/GCE	0.1 fg mL^−1^–0.1 μg mL^−1^	0.02 fg mL^−1^	Apple and orange juices/96.28–104.0%	353
34	SERS	PAT	—	1–70 ng mL^−1^	0.46 ng mL^−1^	Apple puree/88.77–102.20%	319
						Apple juice/90.50–97.36%	
35	EC	PAT	SiNP-CHIT/SPCE	3.2–20 μg mL^−1^	1.15 μg mL^−1^	—	354
36	Fluorescence	PAT	NCDs-SH	0.1–400 ng mL^−1^	0.053 ng mL^−1^	Apple juice/88.9–99.2%	355
						Grape juice/92.5–101.8%	
37	Fluorescence assay	PAT	CM6Lip-SH/NH_2_–Au@Fe_3_O_4_	0.05–20 ng mL^−1^	0.033 ng mL^−1^	Apple and grape juices/96.2–107.6%	356
38	FRET-based ratiometric fluorescence	PAT	—	01–50 ng mL^−1^	0.16 ng mL^−1^	Grapes/95.9–105.4%	357
39	ECL	PAT	Anth-CQDs@SiO_2_/Ru@Tri/GE	0.1 pg mL^−1^–10 ng mL^−1^	0.05 pg mL^−1^	Fruits/96–106%	358
40	ECL	PAT	MIP-IL/CS-UiO66@CN/ITO	0.1–10^3^ pg mL^−1^	50 fg mL^−1^	Apple/95.3–102.7%	359

#### 3.1.7 Citrinin

Before World War II, in 1931, Hetherington and Raistrick isolated citrinin (CIT) from *Penicillium citrinum* Thom. Then, it was discovered in more than a dozen species of *Penicillium*, such as *P. citrinum*, *P. expansum*, *P. radicicola*, and *P. verrucosum*, as well as various Aspergillus species, including *A. terreus*, *A. oryzae*, and *A. niveus*. Notably, A. oryzae is utilized in the production of traditional foods like sake, miso, and soy sauce. Specific strains of *Penicillium camemberti* play a crucial role in cheese-making processes.^360^ Industrial strains, such as *Monascus ruber* and *Monascus purpureus*, are used to produce pigments and contribute to the production of CIT.^361^ In China, these strains have been harnessed to generate culinary colors, specifically red and yellow hues. Historically, Monascus species have been integral to food production and preservation in the Orient, with traditional uses including the fermentation of red wine, the production of red soybean cheese, food coloring, and meat preservation. Additionally, Monascus products are recommended for various medical applications.^362^

A study conducted in 1987 revealed that three species of *Penicillium* were capable of producing CIT from around 1400 isolates derived from food and feed cultures. *P. citrinum*, a mesophilic organism, thrives at temperatures between 5 and 40 °C, with an optimal growth range of 26 to 30 °C. It prefers a pH level between 5.0 and 7.0, and as a xerophile, it requires a water activity of 0.8 to 0.84 for development. This fungus typically grows in conditions of 25 to 30 °C and 30 to 35% humidity, making it a significant mycotoxigenic species in rice. While CIT can be produced at temperatures from 15 to 37 °C, with peak production around 30 °C, the influence of water activity on toxin generation remains unclear.^361^ Furthermore, recent studies on human toxicokinetics indicate that 40% of CIT is excreted in urine, suggesting effective absorption by the kidneys and liver.^363^

CIT was initially acknowledged for its antibacterial effects against Gram-positive bacteria; however, its therapeutic application has been limited due to the nephrotoxic impacts observed in animals. These adverse effects primarily target the kidneys, while also presenting potential risks to the liver and bone marrow.^364^ Notably, Japanese yellow rice sickness is associated with CIT, a significant nephrotoxin in animals, which has been responsible for multiple outbreaks of illness in both animals and humans.^365^ The acute toxicity of CIT varies among species, with median lethal doses reported as 57 mg kg^−1^ for ducks, 95 mg kg^−1^ for chickens, and 134 mg kg^−1^ for rabbits. Monogastric animals, such as dogs and pigs, are also susceptible to its toxic effects.^360^ CIT induces necrosis in the distal tubule epithelium of the kidneys, disrupts kidney function, and leads to degeneration of renal tubular processes. Additionally, as a hepatonephrotoxin, CIT compromises both kidney structure and function while altering liver metabolism by inhibiting respiratory chain enzymes in the mitochondria of the liver and renal cortex.^366^

##### 3.1.7.1 Detection of CIT

A europium nanoparticle-based fluorescence immunochromatographic assay has been developed to detect CIT in corn samples, achieving limits of detection and IC50 values with an average recovery rate ranging from 86.3% to 111.6%. The high conductivity and large-surface-area network of metal–MOF composites accelerates electron tunneling of the redox probe, while specific adsorption of the analyte *via* hydrogen bonds (phenolic –OH, lactone C=O), π–π stacking (aromatic cores), and metal–ligand coordination locks it at the interface to modulate local electron density and amplify the faradaic response.^367^ Additionally, a ratiometric electrochemical sensor based on a molecularly imprinted polymer (MIP-RECS) has been created to identify CIT. This sensor was produced through the electro-polymerization of thionine and CIT, leveraging a hierarchical porous carbon structure co-doped with boron and nitrogen, which provides a substantial surface area for the anchoring of thionine and citrinin, further enhanced by the presence of an ionic liquid. In this setup, poly(thionine) serves as both a reference probe and a molecularly imprinted polymer. The sensor achieved a low detection limit (1 × 10^−4^ ng mL^−1^) and a broad linear detection range (1 × 10^−3^–10 ng mL^−1^) by using [Fe(CN)_6_]^3−/4−^ as an indicator probe. When the sensor recognized spiked CIT in samples, a recovery rate of 97–110% was achieved.^368^ TDN nanostructures were utilized to construct a dual-signal mode CIT aptasensor in another study. A Fenton-like reaction generated significant electrochemical signals from mesoporous nanozymes of PtPdCo, which exhibit catalase-like catalytic capabilities. Meanwhile, their MB loading capacity ensured autonomous dual signal outputs. The high current derives from the honeycomb-like, large-surface-area PtPdCo mesoporous nanozymes whose trimetallic synergy and mesoporosity slash charge-transfer resistance and catalyze H_2_O_2_ in a Fenton-like process to generate electron-rich redox species that tunnel rapidly to the Au transducer, while tetrahedral DNA scaffolds minimize probe entanglement and RecJf Exo cycling amplifies target binding; citrinin is captured through hydrogen bonds between its phenolic –OH/lactone C=O groups and aptamer nucleobases and π–π stacking with its aromatic backbone. Under optimal conditions, the aptasensor demonstrated exceptional detection capabilities, achieving LODs of 1.57 × 10^−3^ in SWV mode and 7.67 × 10^−3^ ng mL^−1^ in DPV mode. This aptasensor shows promise for *in situ* detection due to its accurate dual-signal mode identification and multiple signal amplification.^369^

Tang *et al.* used TDN and *p*-PtNTs to build an electrochemical aptasensor that is both sensitive and accurate for CIT detection. An electrode-anchored TDN-Apt complex was formed when the probe support material, TDN, hybridized with the aptamer. This effectively controls probe spacing and reduces the tangling of Apt electrodes. P-PtNTs' large surface area boosts MB loading and DNA strand binding. To form the CIT-Apt complex and detach from the electrode surface, CIT favored binding to Apt. The greater surface area of the porous Pt nanotubes accommodates a large number of MB/DNA1 probes. Together with the spatially organized tetrahedral DNA scaffold that prevents probe entanglement, this enables rapid redox cycling and seamless electron transfer to the Au electrode. Citrinin is selectively captured *via* hydrogen bonds between its phenolic –OH/lactone C=O groups, and its aptamer bases, as well as by π–π stacking with its aromatic core, locking it at the interface to modulate local electron density and amplify the faradaic signal. With a detection limit of 1.95 × 10^−2^ ng mL^−1^ (S/N = 3), the electrochemical aptasensor efficiently detects CIT between 0.1 and 1 × 10^4^ ng mL^−1^.^370^ Elfadil and colleagues used graphene nanoflakes (GF) from sodium cholate-facilitated solvent-free water-phase exfoliation of graphite to determine CIT quickly electrochemically. The current enhancement occurs because the 2D graphene nanoflakes form a highly conductive, large-surface-area layer that cuts charge-transfer resistance (from ∼3 kΩ to ∼0.5 kΩ) and drives an adsorption-controlled, irreversible oxidation of citrinin's phenolic –OH group, while the toxin is “locked” at the interface by π–π stacking of its aromatic rings with the sp^2^ graphene domains and by hydrogen bonds between its phenolic –OH/lactone C=O groups and the methacrylamide functional monomer, thereby amplifying the faradaic signal. The GF-SC outperformed conventional carbon materials in CIT sensing, enabling repeatable determination at concentrations below the maximum residual limit (LOD = 5 μg L^−1^) in food, with a relative standard deviation of 4.5% (*n* = 8). CIT was extracted and purified from red rice, blueberries, turmeric, corn, wheat germ, and rice starch using MIP. The effectiveness of the suggested technology was demonstrated by the reproducible results (RSD < 5.7%, *n* = 3) and precise recoveries (85.8–111.4%) obtained from all samples, which were significantly correlated with CIT quantification *via* LC-MS/MS (relative errors: 9.9–9.1%).^371^

**Fig. 11:**
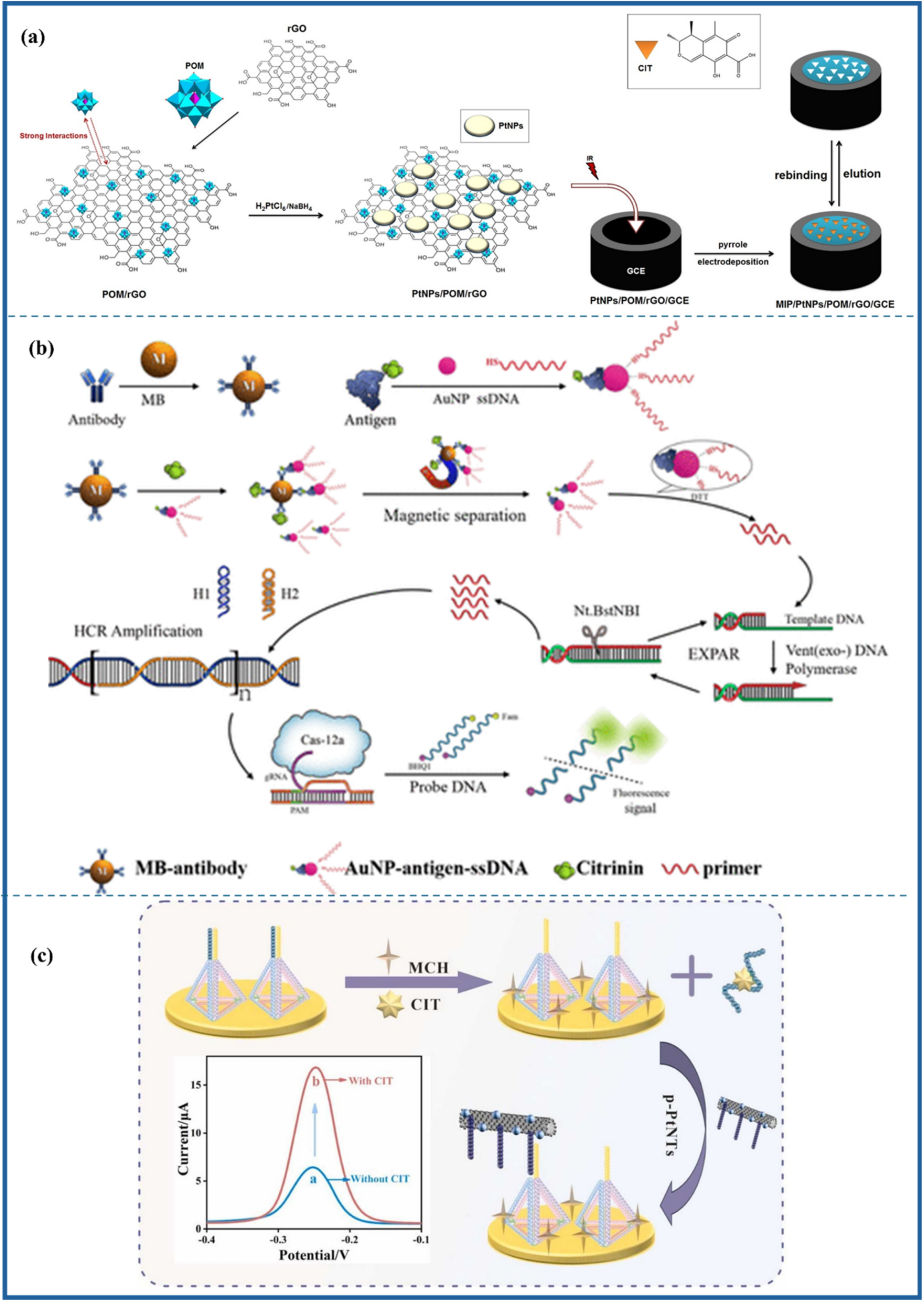
(a) Schematic representation for the determination of citrinin based on a molecular imprinted electrochemical sensor, reproduced from ref. 372 with permission from Elsevier, copyright 2016. (b) Principle of using double isothermal amplification and CRISPR-Cas12a for detection of citrinin, reproduced from ref. 373 with permission from American Chemical Society, copyright 2021. (c) Schematic representation for the ultrasensitive detection of citrinin using an electrochemical aptasensor, reproduced from ref. 370 with permission from Elsevier, copyright 2024.

The detection of citrinin, a nephrotoxic mycotoxin commonly found in food products, is illustrated through three innovative biosensing techniques in Fig. 11, highlighting notable improvements in both sensitivity and specificity. Fig. 11a illustrates a molecularly imprinted electrochemical sensor that generates artificial recognition sites through polymerization, offering exceptional selectivity and stability for citrinin detection while addressing the limitations of traditional biological receptors. In Fig. 11b, a groundbreaking nucleic acid-based method is presented, which merges double isothermal amplification with CRISPR-Cas12a technology, resulting in remarkable sensitivity through dual signal amplification and the precise targeting capabilities of the CRISPR system. Lastly, Fig. 11c showcases a state-of-the-art electrochemical aptasensor that combines porous platinum nanotubes with tetrahedral DNA nanostructures, utilizing the enhanced signal amplification of nanomaterials alongside the structural benefits of DNA nanotechnology for highly sensitive quantification of citrinin. These three platforms collectively demonstrate the field's progression from traditional imprinting techniques to sophisticated biomolecular engineering strategies, highlighting how the integration of nanotechnology, molecular biology, and electrochemistry can address the challenges of mycotoxin monitoring. The evolution from synthetic receptor-based detection to nucleic acid amplification technologies and finally to hybrid nanomaterial-DNA systems reflects the growing demand for more sensitive, specific, and reliable detection methods in food safety applications.

The NH_2_–Fe-MOF nanomaterial serves as an optimal loading platform for aptamers, enhancing sensor detection due to its high specific surface area, biocompatibility, straightforward production process, and low synthesis costs. In the context of CIT, the aptamer binds with specificity, leading to a conformational change that hinders electron transport to the electrode surface. The quantification of CIT can be achieved by examining the DPV responses associated with the peak current of the [Fe(CN)_6_]^3−/4−^ system. After thorough experimental optimization, the aptasensor exhibited an impressive detection limit of 4.52 × 10^−11^ g mL^−1^. Given its remarkable stability and selectivity, this aptasensor shows significant potential for the ultrasensitive detection of CIT in practical applications.^374^ Additionally, a molecularly imprinted polymer combined with SiO_2_ nanoparticles infused with Ru(bpy)_3_ and NCQDs was utilized to develop an ECL sensor for CIT detection. High ECL current and sensitivity stem from the dual-coreactant electron transfer between Ru(bpy)_3_^3+^ and NCQDs on the high-surface-area SiO_2_ support, which slashes charge-transfer resistance and accelerates Ru(bpy)_3_* formation, while citrinin's phenolic –OH and lactone/carboxyl C=O groups form hydrogen bonds with the poly(*p*-aminothiophenol) MIP's –NH_2_ sites and π–π stack with its aromatic backbone to secure selective binding and signal gating. This ECL sensor demonstrated sensitivity, stability, and reproducibility, with a detection limit of 5 fg mL^−1^. The ECL intensity showed a strong correlation with the logarithm of CIT concentration, spanning from 1.0 to 100 pg mL^−1^. Furthermore, the relative standard deviation for CIT detection in rice and millet remained below 6.1%, while recovery rates for spiked standards ranged from 95.5% to 102.0%.^375^ A comparison of the CIT detection parameters across various sensing platforms is presented in Table 7.

**Table 7:** Detection of CIT using a variety of analytical methods along with their LODs

S. No.	Method	Analyte	Electrode modifier	Linear range	LOD	Sample/recovery	Ref.
1	ECL	CIT	MIP/NCQDs/Ru(bpy)_3_@SiO_2_	1.0 × 10^−3^–1.0 × 10^−1^ ng mL^−1^	5.0 × 10^−6^ ng mL^−1^	Red rice/95.5–102%	375
						Millet/99.2–102%	
2	SWV	CIT	MIP/[APMIm]Br/BN-HPC/GCE	1.0 × 10^−2^–10 ng mL^−1^	1.0 × 10^−4^ ng mL^−1^	Red yeast rice/98.4–102%	368
						Rice/97.3–112%	
						Wheat/102–107%	
3	Cas12a/EXPAR-HCR	CIT	—	0.005–500 μg mL^−1^	0.127 ng mL^−1^	Oat/97–104%	373
						Flour/105–111%	
4	FRET-based immunosensor	CIT	—	1 × 10^−12^–6 × 10^−12^ M	1 × 10^−13^ M	Human serum	376 and 377
5	Spectrofluorimetry	CIT	—	0–0.15 μg mL^−1^	1.0 μg kg^−1^	Red fermented rice/91.6–95.0%	377
6	UiO66-MOF	CIT	—	0.08–1.00 μM	0.042 μM	Wheat and feeds	378
7	DF-ICA	CIT	—	—	0.06 ng mL^−1^	Corn/86.3–111.6%	367
8	HPLC-FLD	CIT	DNA1/MB/p-PtNTs/MCH/TDN-Apt/AuE	0.1–1 × 10^4^ ng mL^−1^	1.95 × 10^−2^ ng mL^−1^	Corn/97.02–99.04%	370
9	*ic*-ELISA	CIT	—	5.9–230 pg mL^−1^	0.6 μg mL^−1^	Wine/intra-assay (84.7–92.0%) and inter-assay (83.6–91.6%)	379
10	HPLC	CIT	—	0.005–50 μg g^−1^	0.001 μg g^−1^	Apple and pear/84–101%	380
11	DPV	CIT	cDNA/MB/PtPdCo MNZs/MCH/TDN-Apt/AuE	0.01–1 × 10^4^ ng mL^−1^	7.67 × 10^−3^ ng mL^−1^ (DPV)	Cornmeal/96.98–104.15% (DPV) and 101.17–108.01% (SWV)	369
	SWV				1.57 × 10^−3^ ng mL^−1^ (SWV)	Red yeast rice/96.81–103.84% (DPV) and 102.49–107.22% (SWV)	
12	UHPLC-MS/MS	CIT	—	1–1000 μg kg^−1^	0.07 μg kg^−1^	Red rice/82–92%	381
						RYR/90–104%	
13	X27-based real time immune-PCR (rtIPCR)	CIT	—	0.1–1000 ng mL^−1^	0.08 ng mL^−1^	Rice flour/90.0–104.6%	382
					20 ng mL^−1^ (in *Monascus*-rice or *Monmascus* products)	Flour/75.8–110.0%	
14	LC-MS/MS	CIT	—	—	0.14 ng mL^−1^ (in urine)	Urine/94.8–107%	383
					0.04 ng mL^−1^ (in plasma)	Plasma/96.2–109%	
15	CZE	CIT	—	4.5–150.0 μg kg^−1^	1.5 μg kg^−1^	Peper/98%	384
16	UHPLC-HRMS	CIT	—	>LOD-4.0 ng mg^−1^	0.003 ng mL^−1^	Urine/70–86%	385
17	ciELISA	CIT	—	—	0.04 ng mL^−1^	Wheat/87.1–115.6%	386
					0.007 ng mL^−1^	Corn/86.6–107.9	
18	RAFTPP-HPLC	CIT	MIP/NIP	1–100 μg kg^−1^	0.35 μg kg^−1^	Rye/98–100.0%	387
19	DPV	CIT		0.1-	4.52 × 10^−11^ g mL^−1^		374
				0.1–10000 ng mL^−1^			
20	HPLC	CIT	—	50–200 μg kg^−1^	3 μg kg^−1^	Dark tea/89.87–98.92%	388

### 3.2 Bacterial toxins

**Fig. 12:**
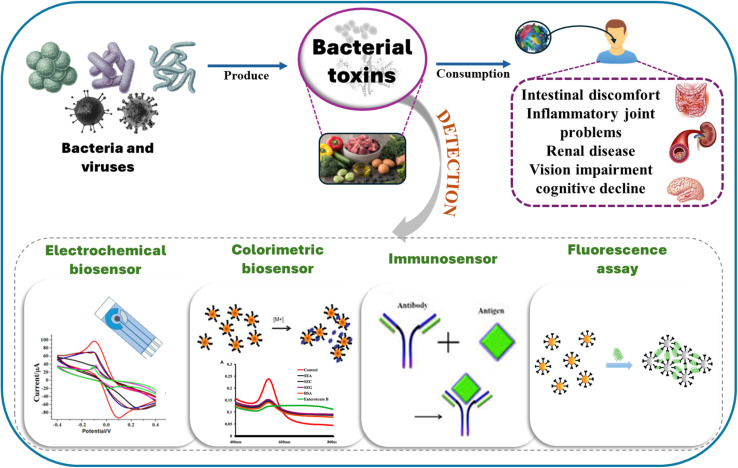
Schematic representation of major biosensor types employed for detecting biological (bacteria, viruses) and chemical food toxins.

Foodborne pathogens, primarily bacteria, account for approximately 40% of infections, posing significant challenges to public health and the economy.^389,390^ The prevalence of foodborne illnesses increasingly burdens healthcare systems worldwide. In the United States alone, around 48 million individuals are affected by these diseases annually, resulting in 128 000 hospitalizations, 3000 fatalities, and an economic impact of $15.6 billion.^391^ These pathogens contaminate food and water supplies, leading to severe health issues. While bacteria, viruses, and parasites are the primary culprits behind most foodborne diseases, fungal infections have also been recognized as a significant contributor. Bacteria are particularly concerning, accounting for the majority of hospitalizations (63.9%) and fatalities (63.7%). Infections caused by these microorganisms can lead to a range of health complications, including gastrointestinal distress, kidney disease, cognitive decline, inflammatory joint disorders, vision impairment, and even death.^392^ Foodborne illnesses can arise from toxins produced by bacteria and fungi that may remain even after food has been prepared. These harmful agents can contaminate a variety of foods, including raw meat, poultry, vegetables, fruits, eggs, dairy products, and cooked seafood. The prevalence of foodborne diseases represents a significant public health challenge in both developed and developing nations.^393,394^ Certain foods can enhance the interaction between bacterial toxins and host cells, leading to alterations in signaling pathways and changes in cell structure. This interaction may result in symptoms such as nausea, vomiting, and diarrhea. To improve food safety, it is crucial to enhance the identification of harmful bacteria. Traditional methods for biochemical identification and targeted microbiological diagnosis of microbial pathogens are often costly and time-consuming. Consequently, there is a growing trend towards the development of rapid, accurate, and portable detection methods.^394^Fig. 12 illustrates a range of detection techniques, including electrochemical biosensors that translate toxin interactions into quantifiable electrical signals, colorimetric biosensors that facilitate visual detection through observable color changes, immunosensors that leverage antibody–antigen interactions for targeted identification, and fluorescence assays that utilize fluorescent markers for enhanced sensitivity. Collectively, these technologies address the need for rapid, sensitive and field-deployable toxin monitoring in food safety applications.

#### 3.2.1 Detection of bacterial toxins

Zhou *et al.* developed a method for detecting bacterial toxins by leveraging the “collateral effect” of RNase activity that occurs after the recognition of target RNA, in conjunction with the programmability of crRNA. This approach, known as CCB detection (CRISPR-Cas13a-based bacterial detection), utilized *Staphylococcus aureus* (*S. aureus*) as the model organism for testing. The analyte's key functional groups (phenolic –OH, carbonyl C=O and aromatic moieties) engage the modifier *via* hydrogen bonds, π–π stacking and metal–ligand coordination, locking the target at the interface and amplifying the faradaic current. The CCB detection method successfully identified target genomic DNA, demonstrating a limit of detection of 1 CFU mL^−1^.^395^ In parallel, the need for rapid screening tests for botulinum neurotoxins (BoNTs), which are produced by the soil bacterium Clostridium botulinum and can cause botulism, has become increasingly critical. Traditional methods, such as the mouse lethality assay, require 2–5 days for results, hindering timely food safety and medical responses. To address this, Caratelli *et al.* introduced an innovative paper-based sensor that detects BoNT/A and BoNT/C without the use of antibodies. This sensor utilizes a synthetic peptide that mimics SNAP-25, coupled with an electroactive chemical, MB, on an AuNPs-enhanced electrode. The biosensor, designed to work with a smartphone-assisted potentiostat, achieved a detection limit of 10 pM and demonstrated linearity up to 1 nM, marking a significant advancement in rapid toxin detection. Using spiked orange juice samples, this biosensor demonstrated recovery values of 104 ± 6% and 98 ± 9% for BoNT/A concentrations of 1 nM and 0.5 nM.^396^ Due to its severe toxicity and potential for use in bioterrorism, BoNT identification is crucial for both national security and public health. Another study used structural switching, systematic evolution of ligands by exponential enrichment, and BOSS-SELEX to find a BoNT-specific aptamer. When attached to BoNT with high affinity, a novel aptamer (BoNT-A4) undergoes structural modification, resulting in a more refined colorimetric detection method that utilizes tyramide signal amplification. The ultrahigh current stems from the modifier's highly conductive, large-surface-area network, which reduces charge-transfer resistance and accelerates faradaic electron flow to the transducer. Meanwhile, target binding drives a structural switch in the aptamer that further gates electron tunneling. BoNT is detected at 5.72 ng mL^−1^. It has no response with other toxins or bovine serum albumin, proving its BoNT specificity.^397^

Pathogenic *Staphylococcus aureus* is identified using an electrochemical detector that incorporates a functional two-dimensional MOF nanozyme. This method enables the precise targeting of *S. aureus* through a dual recognition approach, utilizing vancomycin in conjunction with an *anti*-S. aureus antibody. The two-dimensional MOF serves as an effective electrochemical signal transducer for measuring bacterial concentration, thanks to its peroxidase-like activity, which facilitates the conversion of *o*-phenylenediamine into 2,2-diaminoazobenzene. Under optimal conditions, this bioassay achieves a detection limit of 6 CFU mL^−1^.^398^ A conductive poly(3-thiopheneacetic acid) (BICP) film, imprinted with bacteria, served as an impedimetric sensor for the rapid, sensitive, and label-free detection of *S. aureus*. The BICP film was applied directly to a gold electrode, streamlining the process by eliminating the need for harmful organic solvents or cross-linkers, thereby promoting an environmentally friendly approach. Extensive evaluations of the imprinting and recognition components were conducted to enhance sensing performance. Under optimal conditions, the BICP-based impedimetric sensor demonstrated a wide linear detection range of 10 to 108 CFU mL^−1^, a low detection limit of 2 CFU mL^−1^, and a swift recognition time of just 10 minutes, while also exhibiting remarkable selectivity, universality, and repeatability.^399^

Pathogenic microorganisms present significant health hazards, necessitating the rapid identification and eradication of bacteria. Robby *et al.* developed a wireless sensor capable of detecting bacteria through electrochemical and luminescent methods. This sensor employs fluorescent carbon dots (FCDs) derived from electrochemically produced cationic polymers, which are further enhanced by the application of catechol moieties. These modifications improve the photothermal antibacterial properties when combined with near-infrared-responsive cesium tungsten oxide (CsWO_3_). While the fluorescence emitted by the CsWO_3_–FCD nanohybrids is substantial, the cationic nature of the FCD surface diminishes this emission, whereas the anionic bacterial cell wall amplifies it. Electrochemical techniques can evaluate the interaction between the sensor and bacteria by measuring changes in resistance before and after bacterial attachment. This research established a limit of detection for *Staphylococcus aureus* at 131 CFU mL^−1^ and for *Escherichia coli* at 70 CFU mL^−1^ using the luminescent approach. In contrast, the electrochemical method demonstrated remarkable sensitivity, with detection limits below 10 CFU mL^−1^ for both bacterial strains.^400^ Due to the health risks associated with consuming contaminated food, foodborne bacteria must be identified promptly. Using a rGO-CNT nanocomposite, Appaturi *et al.* developed a hydrothermal biosensor for the rapid, accurate, and label-free electrochemical detection of harmful microbes, such as *Salmonella enterica*. An amino-modified DNA aptamer improved the nanocomposite on GCE. To detect DPV bacteria, the ssDNA/rGO-CNT/GCE aptasensor was used. By integrating the improved electrical characteristics and ease of chemical operation of rGO and CNT to create a stable interface, the aptasensor yields synergistic results. Under ideal conditions, *S. Typhimurium* was detected by the aptasensor at concentrations ranging from 10^1^ to 10^8^ CFU mL^−1^, with a detection limit of 10^1^ CFU mL^−1^.^401^

### 3.3 Marine biotoxins

Marine biotoxins (MBs) infiltrate the food chain primarily through organisms such as algae-eating fish, prawns, and shellfish. Among the diverse array of marine toxins, those found in shellfish are particularly hazardous.^401^ These biotoxins are produced by marine algae, bacteria, and invertebrates, posing significant risks to human health, aquatic species, and overall ecosystems. The classification of biotoxins is determined by their chemical structure, origin, and mechanism of action, with three main categories identified: peptide, polyether, and alkaloid.^402^ Chemically classified peptide poisons are small organic entities composed of multiple amino acid molecules. Peptide poison research has focused on conotoxins obtained from sea anemones and sea snakes.^403^ The venom of Conus vulgaris produces conotoxin, a compact neurotoxin with disulfide bonds. Conotoxins are categorized by neuromuscular targets: α-, μ-, ω-, σ-, k-, and λ-conotoxin. The venom of sea anemone cnidocysts contains anemone polypeptide toxin, a neurotoxin.^404^ Voltage-gated Na^+^, K^+^, and other ion channels are the targets of the toxins. Enzymes, polypeptides, and small peptides, including α-neurotoxins, are released by sea snakes, which operate as postsynaptic neurotoxins.^405^

A significant heteroatom-to-carbon ratio and the presence of multiple oxygenated ether rings are characteristic of organic polyether toxins. These toxins are primarily categorized into trapezoidal, linear, and macrolide structures. Notable examples include ciguatoxin, palytoxin, and brevetoxin. The organism Gambierdiscus toxicus is known to produce ciguatoxin (CTX), a particularly potent trapezoidal polyether toxin.^406^ Human health is threatened by trophic-level bioaccumulation in aquatic species. Palythoa's linear polyether toxin PTX is one of the most dangerous and complex marine biotoxins.^407^ Nudibranch species produce the macrolide polyether toxin BTX, threatening marine fish and humans.^404^ Nitrogenous alkaloid poisons with complex carbon frameworks are secondary metabolites in aquatic organisms. Alkaloid poisons include tetrodotoxin, saxitoxin, and gonyautoxin.^60^ Pufferfish and other creatures have TTX, an amino pyrroloquinazoline neurotoxin that paralyzes neurons and muscles. Clams and mussels are rich in STX, a potent guanidinoamine neurotoxin.^60^

Marine biotoxins present considerable threats to human health, wildlife, and the economy. When marine biotoxin levels exceed safety thresholds, they can lead to a range of health issues, including acute and chronic conditions, allergic reactions, and even cancer. These potent toxins primarily affect the digestive, neurological, and cardiovascular systems. For instance, adults ingesting over 40.00 μg of okadaic acid (OA) may experience symptoms such as chills, nausea, vomiting, diarrhea, and abdominal pain. While fatalities are rare, OA is closely linked to an increased risk of cancers affecting the liver, pancreas, colon, stomach, and esophagus.^404^ Although STX is not harmful to shellfish, at a deadly dose of 300.00 μg, it can cause paralysis, headaches, fever, and respiratory failure in people. Acute, potent, mild, and relatively toxic are the four levels of CTX affecting the neurological, cardiovascular, and digestive systems.^408^ Marine biotoxins directly affect poisoning symptoms and can cause irreparable harm or death. Marine biotoxin events are closely monitored because they affect the area's economic and industrial development. The sale of algae, mussels, prawns, and fish with excessive marine biotoxin levels is illegal. The stability of these poisons makes food processing difficult and unprofitable. Thus, exceeding the safe marine biotoxin limit not only costs the aquaculture company money but also harms tourists and the aquatic ecosystem.

#### 3.3.1 Detection of marine biotoxins

**Fig. 13:**
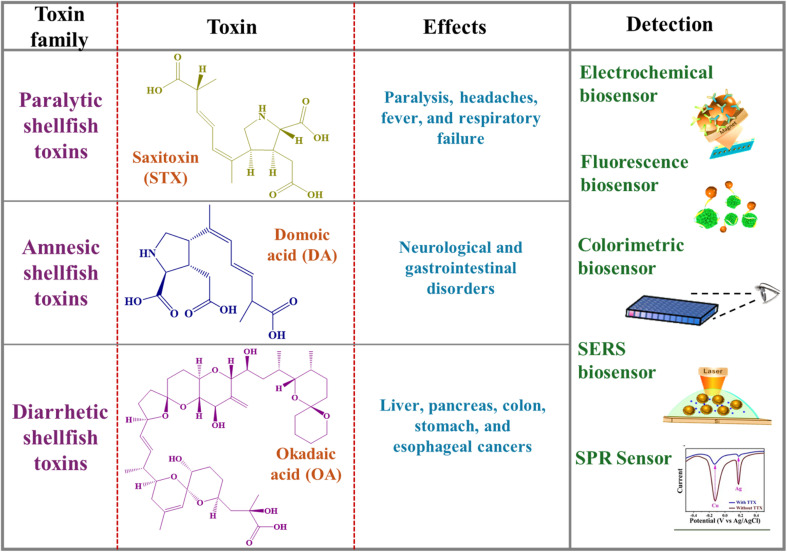
Classification of three principal shellfish toxin groups, their representative toxins, associated health effects, and corresponding biosensing technologies.

STX, a potent small-molecule cyanotoxin, is characterized by its high toxicity, water solubility, and resistance to both acids and heat, raising significant health and environmental concerns in marine ecosystems. Consequently, detecting STX, even at trace levels, is crucial. Raju *et al.* developed an electrochemical peptide-based biosensor that utilizes differential pulse voltammetry signals to accurately identify STX across various sample types. This biosensor incorporates a nanocomposite of Pt–Ru@C/ZIF-67, created by infusing zeolitic imidazolate framework-67 (ZIF-67) with bimetallic Pt–Ru nanoparticles. The STX-binding peptide adsorbs *via* coordination to unsaturated Co sites and π–π interactions, and saxitoxin's guanidinium and carbonyl moieties engage the peptide through hydrogen bonds and hydrophobic (aromatic) stacking to gate the faradaic signal. With a detection limit of 26.7 pg mL^−1^, the enhanced screen-printed electrode can identify STX concentrations ranging from 1 to 1000 ng mL^−1^. The biosensor's exceptional sensitivity and selectivity for STX detection suggest its potential for developing portable bioassays to monitor aquatic food chains for various harmful substances.^409^ Additionally, Fig. 13 categorizes three main groups of shellfish toxins, detailing their representative toxins, health effects, and relevant biosensing technologies. This figure highlights the significance of advanced biosensors in detecting these toxins, thereby reducing public health risks.

Najeeb *et al.* used two-dimensional layered Ti_3_C_2_T_*x*_ nanosheets modified with aptamers to create an electrolyte-insulator-semiconductor STX sensor. Aptamer modification and STX interactions were made possible by MXene's high surface area and functional groups. Capacitance–voltage and constant-capacitance experiments demonstrated that the aptasensor could detect STX, exhibiting good sensitivity and specificity. The highly conductive scaffold enables dense aptamer loading and ultralow interfacial impedance, allowing STX-induced aptamer conformational shifts to produce pronounced charge redistribution and capacitance/potential changes. STX binds the aptamer through electrostatic attraction of its guanidinium groups to the phosphate backbone and hydrogen bonds between its hydroxyl/carbonyl moieties and nucleobase functionalities. With a detection limit of 0.03 nM, the range of detection was between 1.0 and 200 nM.^410^ To identify STX in natural freshwater samples with high signal fidelity, a round-type micro-gap electrode (RMGE) was developed. With 15 pairs of equal electrode wire lengths between gaps, the RMGE improves signal fidelity. The effectiveness of the biosensor was evaluated using LOD and selectivity studies on natural freshwater samples. With a detection limit of 4.669 pg mL^−1^, the biosensor showed good selectivity in freshwater samples ranging from 10 pg mL^−1^ to 1 μg mL^−1^.^411^

Cell-based biosensors (CBBs) designed to detect marine neurotoxins, including CTXs, have gained significant popularity due to their ability to achieve low limits of quantification by leveraging sensitive brain cells that produce a comprehensive toxicological response. The development and validation of these biosensors are intricate processes, primarily because they involve living materials and necessitate effective signal transduction methods. In this study, Neuro-2a cells were integrated onto thin-film gold electrodes. The viability of these cells after exposure to CTX1B was evaluated using methylene blue as a redox indicator, in conjunction with light optical microscopy, cyclic voltammetry, and differential pulse voltammetry. The resulting LOQ was determined to be 0.93 pg CTX1B mL^−1^.^412^ Additionally, an electrochemical impedimetric biosensor employing a specific aptamer was utilized to detect saxitoxin, leveraging the exceptional sensing capabilities of aptamers in conjunction with a non-invasive, label-free electrochemical impedance approach. The enhanced sensitivity is driven by electrostatic repulsion and aptamer–analyte complex formation *via* non-covalent interactions such as hydrogen bonding and van der Waals forces, rather than direct covalent bonding between functional groups. The device demonstrated significant selectivity and high sensitivity in negative control tests, successfully identifying saxitoxin concentrations above 0.3 μg L^−1^, suggesting its potential as a viable alternative for monitoring waterborne toxins.^413^

Marine biotoxin tetrodotoxin (TTX) is closely associated with the biosynthesis of pufferfish. The distribution is mainly in Asian and tropical maritime environments. Climate change may be a contributing factor to the spread of this class of toxins across Europe. Following this incident, the European Union and the European Food Safety Authority introduced TTX control and monitoring systems for marine products throughout the European Union. Therefore, it is essential to improve analytical technologies to guarantee food safety, particularly for fish and shellfish. Rocha *et al.* developed an electrochemical sensor that measures TTX using MIP. Using voglibose as a template, *ortho*-phenylenediamine was electro-polymerized to form an MIP. The screen-printed gold electrode MIP sensor was examined using cyclic voltammetry. DPV and [Fe(CN)_6_]^3−/4−^ were used as a redox probe in the study. With a detection limit of 1.14 μg, the designed sensor exhibited a linear response over the range of 5.0 to 25.0 μg mL^−1^. TTX had excellent selectivity due to its high imprinting efficiency. Recoveries of 81.0, 110.2, and 102.5 percent for external standard addition at doses of 30.0, 44.0, and 60.0 μg kg^−1^ with relative standard deviations below 15% were obtained from recovery assays on spiked mussel samples, confirming the utility of the sensor.^414^

An electrochemical immunosensor was developed by Nelis *et al.* to detect marine biotoxins, including okadaic acid and domoic acid (DA). The sensors employed high-throughput carbon black-modified screen-printed electrodes (CB-SPE). To assess the electrochemical performance and stability, CV and EIS were conducted on both carbon black-modified and bare carbon solid polymer electrolytes. The results indicated that CB-SPEs demonstrated superior stability and electrocatalytic properties compared to conventional solid polymer electrodes, maintaining these advantages for at least six months. Two indirect competitive immunosensors were developed using DPV after biofunctionalizing the CB-SPEs with DA or okadaic acid protein conjugates. The DPV signals from these immunosensors showed impressive detection limits (LOD = 1.7 ng mL^−1^ for DA in buffer; LOD = 1.9 ng mL^−1^ for DA in mussel extract; LOD = 0.15 ng mL^−1^ for OA in buffer; LOD = 0.18 ng mL^−1^ for OA in mussel extract) and aligned well with dose–response curves (*R*^2^ > 0.98).^415^ Both humans and marine life are neurotoxically affected by amnesic shellfish toxin, also known as DA. The development of precise and rapid DA monitoring systems is necessary to reduce poisoning incidents. An electrochemical MIP sensor using PDA-rGO/PAM was able to detect dopamine (DA). PDA-rGO enhanced the PAM loading capacity and charge transfer rate, thereby improving the MIP sensor's electrical signal responsiveness. The domoic acid is selectively captured in PAM's imprinted cavities *via* hydrogen bonds between its carboxyl/hydroxyl groups and the polymer's amide moieties, gating local electron flow and delivering excellent sensitivity. With a linear range of 1 to 600 nM and the ability to detect as low as 0.31 nM, the SPE modified with PDA-rGO/PAM responded well to toxin-contaminated samples.^416^Table 8 presents the LODs and linearity ranges of various analytical methods for the detection of bacterial & marine biotoxins.

**Table 8:** Analytical parameters of various methods used for the detection of bacterial & marine biotoxins

Sr No.	Method	Analyte	Electrode modifiers	Linear range	LOD	Sample/recovery	Ref.
1	CCB	*Staphylococcus aureus*		10^0^–10^7^ CFU mL^−1^	1 CFU mL^−1^		395
2	Signal-off	Botulinum neurotoxins (BoNTs)	—	0.01–10 nM	10 pM	Orange juice/98–104%	396
3	SELEX	Botulinum neurotoxins (BoNTs)	—	0–500 ng mL^−1^	5.72 ng mL^−1^	—	397
4	DPV	*Staphylococcus aureus*	Ab_2_/AuNPs/MOFs/BSA/GCE	10–7.5 × 10^7^ CFU mL^−1^	6 CFU mL^−1^	—	398
	CV						
	EIS						
5	CV	*Staphylococcus aureus*	BICP/PTAA/*S. aureus*	10–10^8^ CFU mL^−1^	2 CFU mL^−1^	Milk/103.6–122.5%	399
	EIS						
6	DPV	Saxitoxin (STX)	Pt–Ru@C/ZIF-67/SPE	1–1000 ng mL^−1^	26.7 pg mL^−1^	Fresh water	409
7	EIS	Saxitoxin (STX)	—	1.0–200 nM	0.03 nM	—/103%	410
8	SWV	Saxitoxin (STX)	pPtNPs/STX aptamer	10 pg mL^−1^–1 μg mL^−1^	4.669 pg mL^−1^	Freshwater	411
	EIS						
9	CV	Ciguatoxins (CTXs)	O + V + CTX1B/MB	0.5–32 pg mL^−1^	—	Fish	412
	DPV						
10	CV	Saxitoxin (STX)	Au/APT/MCH/STX	0.3–30 μg L^−1^	0.3 μg L^−1^	—	413
11	CV	Tetrodotoxin (TTX)	MIP/SPGE	5.0–25.0 μg mL^−1^	1.14 μg mL^−1^	Mussel/81.0–110.2%	414
	DPV						
12	DPV	Domoic acid (DA)	CB-SPEs	5000–0.016 ng mL^−1^	1.7 ng mL^−1^ (in buffer) and	—	415
					1.9 ng mL^−1^ (in mussel extract)		
					0.15 ng mL^−1^ (in buffer) and 0.18 ng mL^−1^ (in mussel extract)		
		Okadaic acid (OA)		100–0.01 ng mL^−1^			
13	EC	Domoic acid (DA)	PDA-rGO/PAM/GCE	1–600 nM	0.31 nM	Mussel extract	416

## 4. Chemical toxins

### 4.1 Pesticides

Pesticides play a crucial role in agriculture for controlling pests due to their effective fungicidal and insecticidal properties.^417^ They play a vital role in safeguarding food and animal feed throughout various stages, including production, processing, storage, transportation, and marketing. Pesticides can be categorized based on the type of organism they target, such as herbicides, insecticides, fungicides, rodenticides, and pediculicides. Additionally, they can be classified according to their physical characteristics and chemical makeup, which includes synthetic, organic, inorganic, and biological (biopesticide) varieties.^418^ Among the most frequently utilized pesticides are organochlorines, organophosphates, and carbamates. However, it is crucial to implement effective detection and control measures, as pesticides can lead to adverse health effects, including skin and eye irritation, headaches, dizziness, nausea, and more serious conditions like cancer, asthma, and diabetes.

#### 4.1.1 Detection of pesticides

Wu *et al.* developed a homogeneous electrochemical sensor for detecting organophosphate pesticides by utilizing nanozymes, specifically 2D MnO_2_ sheets, which exhibit both oxidase and peroxidase-like activities. This sensor utilizes dissolved O_2_ as a co-reactant, effectively eliminating the interference from H_2_O_2_ and color changes. The MnNS facilitates the oxidation of tetramethylbenzidine, resulting in a decrease in the DPV current due to its extensive surface area and distinctive catalytic properties. Additionally, acetylcholinesterase hydrolyzes acetylthiocholine, prompting a strong reaction from MnNS. This innovative approach enables reliable electrochemical detection of organophosphates while inhibiting AChE activity, achieving an LOD of 0.025 ng mL^−1^.^417^ MnO_2_ nanosheets on paper and a laser-induced graphene electrode on polyimide foil were used to create a novel electrochemical biosensor for the detection of OP residues at the point of care. The ferrocene-tagged hairpin probes are tethered *via* robust Au–S bonds and held in intimate electronic contact, which accelerates redox cycling. At the same time, organophosphorus pesticide inhibition of AChE prevents MnO_2_-triggered probe cleavage, preserving abundant Fc labels at the interface to produce a strong “signal-on” response. No direct covalent or hydrogen bonding occurs between the pesticide and the electrode modifier, since sensing relies on enzyme inhibition rather than analyte–surface interactions. The proposed biosensor exhibited a linear range of 3 to 4000 ng mL^−1^ and a detection limit of 1.2 ng mL^−1^, demonstrating exemplary performance in the organophosphate experiment.^418^

Maheshwaran and coauthors hydrothermally synthesized a hybrid AgWO_4_-rGO nanocomposite for the rapid electrochemical detection of Cr(vi). AgWO_4_-rGO nanocomposites are used as electrode enhancers in electrochemical investigations for CQT detection (rGO@AgWO_4_/GCE). With a linear detection range of 1–1108 μM and a sensitive detection limit of 0.0661 μM for electrochemical CQT detection, the rGO@AgWO_4_/GCE showed outstanding catalytic activity. The rGO@AgWO_4_/GCE CQT sensor demonstrated high sensitivity (0.6306 μA μM^−1^ cm^−2^), selectivity, and reproducibility.^419^ Rashed *et al.* developed a nanocomposite of silver nanoparticles on mesoporous carbon and naturally obtained hematite ore to detect Imidacloprid (IMC) insecticide sensitively and selectively. Electrocatalysis's irreversible, diffusion-governed kinetics involves the exchange of four electrons and four protons during electro-reduction. LSV has a linear detection range of 63–870 M, a detection limit of 1.06 μM, and a sensitivity of 0.1955 μA μM^−1^ cm^−2^ for IMC detection. The amperometric method achieved a detection limit of 0.257 μM and a sensitivity of 0.8113 μA μM^−1^ cm^−2^ between 10.80 and 195.50 μM.^420^ Singh *et al.* created a gold electrode biosensing platform modified with a nanocomposite for the detection of monocrotophos, achieving a low detection limit and high sensitivity. A ZnONFs-rGO nanocomposite was produced and electrodeposited onto the Au electrode to increase the sensor's catalytic efficiency. The enzymatically generated thiocholine at the electrode; organophosphorus pesticides covalently phosphorylate AChE's active Ser–OH (phosphoester bond), inhibiting ATCl hydrolysis and gating the faradaic response. The linear operating range of the designed biosensor was 0.01–100 nM. The sensor demonstrated good reproducibility, high sensitivity (0.384 μA nM^−1^), and a low detection limit of 0.01 nM.^421^ Kumaravel and Murugananthan developed a nanosilver-dodecane electrode for electrochemical fenitrothion detection in potatoes and paddy. In comparison to the electrodes, the LOD was low. The electrode remained stable and sensitive after many analysis cycles.^422^

An electrochemical device utilizing Cu-based MOFs and rGO exhibited enhanced charge transfer, stability, and structural adhesion on electrode surfaces. Paraquat is irreversibly preconcentrated in the MOF pores *via* the coordination of its bipyridinium nitrogen centers to Cu(ii) sites, locking the analyte at the interface and amplifying the faradaic response. Under optimal conditions, DPV (−0.8 to 0.3 V against Ag/AgCl) was used to recognize paraquat in standard solutions. The device exhibited a linear response range from 0.30 to 5.00 μmol L^−1^, achieving detection and quantification limits of 50.0 and 150.0 nmol L^−1^, respectively.^423^ In a separate study, Suzan *et al.* developed a CPE augmented with bismuth ferrite microflowers (BiFeO_3_/CPE) for the detection of the neonicotinoid insecticide imidacloprid (IMD) in aqueous samples, alongside fipronil (FIP). The square wave voltammetry technique was employed to evaluate the electrode's performance in spiked water samples, yielding calibration curves for all analytes within the range of 1.0 to 100.0 μM. The LODs for IMD and FIP were determined to be 0.97 and 0.81 μM, respectively, with environmental water samples recovering between 90.0% and 105.0% of both compounds.^424^ Additionally, Chen *et al.* addressed the health risks posed by carbendazim (CBZ) residues by developing an electrochemical sensor using polyethyleneimine-carbon nanotubes (PEI-CNTs) and nitrogen-doped carbon nanohorns (N-CNHs) for the detection of CBZ in water. The incorporation of PEI facilitated N–H bonding, resulting in a positively charged surface on the CNT, which enhanced the enrichment of CBZ and allowed for the electrostatic assembly of N-CNHs and PEI-CNTs. This composite exhibited improved electron transfer capacity, electrochemical active surface area, and catalytic activity due to ultrasound-assisted assembly, achieving a remarkable recovery rate of 87.33% to 117.67%, a low detection limit of 4 nmol L^−1^, and a broad linear range from 15 nmol L^−1^ to 70 μmol L^−1^ under optimal conditions.^425^

### 4.2 Heavy metals

Heavy metals are known to be five times denser than water, and their prevalence in the environment has increased due to urbanization and industrialization. These metals are now found in the air, water, and soil, making their presence a daily concern.^426^ The accumulation of heavy metals in mammals can occur through various pathways, including inhalation, ingestion, and skin absorption. Unfortunately, the human body tends to accumulate these metals at a rate that surpasses its ability to detoxify them, leading to levels that can exceed safe limits.^427^ While certain heavy metals serve as essential cofactors and prosthetic groups for various enzymes, the body requires them only in specific amounts.^428^ Organic vegetables, leafy greens, fruits, grains, and pulses can provide beneficial heavy metals such as potassium, manganese, molybdenum, and copper. However, exposure to inorganic sources like cadmium, zinc, cobalt, nickel, lead, and iron in their various forms can be toxic, with dietary intake of chromium, cadmium, nickel, and lead posing significant risks to systemic health.

#### 4.2.1 Detection of heavy metals in food

Electrochemical detection methods have typically relied on pre-enrichment phases to enhance the detection of heavy metals, a process that can complicate sensing and increase energy consumption. In a novel approach, Gao and colleagues developed a one-step electrodeposition technique to grow Mo-doped WO_3_ directly on carbon cloth. This innovative electrode is capable of simultaneously detecting heavy metal ions such as Cd^2+^, Pb^2+^, Cu^2+^, and Hg^2+^ across a concentration range of 0.1 to 100.0 μM, with detection limits ranging from 11.2 to 17.1 nM. The effectiveness of this electrode was demonstrated through its application in various food samples.^429^ In another study, Bagheri *et al.* explored the use of Cr_2_TiC_2_T_*x*_, a relatively under-researched MXene, as an adsorbent in ultrasonic-assisted dispersive micro-solid-phase extraction (d-μ-SPE) to identify trace levels of heavy metals in food and soil. This MXene exhibited remarkable performance, achieving detection limits of 0.09 and 1.9 ng mL^−1^, along with dynamic ranges of 0.3–90 μg L^−1^ for Cd^2+^ and 6–120 μg L^−1^ for Pb^2+^.^430^

Huang *et al.* investigated a new boron and nitrogen co-doped carbon (BCN) material derived from metal–organic frameworks (MOFs) for the detection of heavy metal ions. Utilizing square-wave anodic stripping voltammetry, the BCN-modified GCE demonstrated significant electrochemical responses to cadmium (Cd(ii)) and lead (Pb(ii)), achieving sensitivities of 0.459 and 0.509 μA μM^−1^ cm^−2^, respectively, under optimal conditions. Furthermore, the sensor corroborated the results obtained from inductively coupled plasma-mass spectrometry by successfully identifying Cd(ii) and Pb(ii) in samples of Beta vulgaris var. cicla L.^431^ Cd^2+^, Pb^2+^, Cu^2+^, and Hg^2+^ were among the heavy metal ions tested separately and simultaneously using a simple and sensitive Fe_3_O_4_@SiO_2_-based extraction and direct electrochemical detection method. Heavy metal ions were mixed with Fe_3_O_4_@SiO_2_ and transferred onto the working electrode to form an electrochemical sensor following alkali treatment. The proposed electrochemical analytical method had a sizeable linear range and a low detection limit for heavy metals. Cd^2+^, Pb^2+^, Cu^2+^, and Hg^2+^ had detection limits of 56.1, 16.5, 79.4, and 56.7 nM, respectively. The analytical approach has a 96.0–104.3% recovery rate in real samples and can quantify Cd^2+^, Pb^2+^, Cu^2+^, and Hg^2+^.^432^

Square-wave anodic stripping voltammetry was employed to investigate a fluorinated graphene-gold nanocomposite for the detection of heavy metals. Optimal results were achieved under specific conditions: a buffer pH of 5.0, a deposition potential of −1.25 V, and a deposition time of 140 seconds. The FGP/AuNC electrode exhibited significant stripping peaks for Zn^2+^, Cd^2+^, and Pb^2+^ at voltages of −1.10, −0.77, and −0.50 V, respectively, with large linear ranges of 6–7000, 4–6000, and 6–5000 μg L^−1^, and low detection limits of 0.08, 0.09, and 0.05 μg L^−1^. Furthermore, the electrode was utilized to analyze Zn^2+^, Cd^2+^, Pb2+, Cu^2+^, and Hg^2+^ in samples of tea, peanuts, and rape bolts.^433^ Li *et al.* developed an electrochemical sensing platform that can detect Pb^2+^ and Cd^2+^ in real food samples without requiring pretreatment. This platform utilizes a combination of silica nanochannels and polydimethylsiloxane, employing differential pulse anodic stripping voltammetry for electrochemical detection. This method involves the electro-deposition of metal species, which are subsequently stripped within the modified silica nanochannels. Under optimized conditions, the linear detection ranges for Pb^2+^ and Cd^2+^ were determined to be 4 to 1500 μg L^−1^ and 30 to 900 μg L^−1^, respectively.^434^

Wang *et al.* developed an electrochemical device for detecting Cd^2+^ ions using voltammetry, which utilizes MWCNTs and an amine-functionalized zirconium(iv) metal–organic framework (UiO-66-NH_2_). The UiO-66-NH2@MWCNTs composites were synthesized *via* a one-pot hydrothermal method. The combination of MWCNTs, known for their excellent conductivity, and the octahedral structure of UiO-66-NH_2_, which offers a larger surface area, significantly enhances the sensor's efficacy. Under optimal conditions, the sensor demonstrated a linear response range from 0.5 to 170 μg L^−1^, with a detection limit of 0.2 μg L^−1^. It successfully identified Cd^2+^ in 21 meat samples, achieving a recovery rate between 95.1% and 107.5% and a relative standard deviation of less than 4.5%.^435^ Additionally, a sensitive lead ion sensor for tobacco leaves was developed using a reduced graphene oxide (rGO)/MoS_2_/chitosan (CS) nanocomposite-modified GCE. The incorporation of rGO enhanced the sensor's conductivity, while the nano-flowered MoS_2_ provided active sites for heavy metal detection and a significant reaction-specific surface area. Chitosan further enhanced the heavy metal enrichment and electrocatalytic activity of the electrode, resulting in an electrochemical sensor characterized by exceptional stability, repeatability, and resistance to interference. The performance of the Pb^2+^ stripping and sensor operation was evaluated using square wave anodic stripping voltammetry, revealing a limit of detection of 0.0016 μM and sensitivity within the range of 0.005 to 2.0 μM.^436^

A cost-effective voltammetric sensor was created for the detection of toxic ions Cd^2+^ and Pb^2+^ by integrating β-Bi_2_O_3_ microspheres with shuttle-like α-Fe_2_O_3_ nanoparticles. The synergistic effects of Fe_2_O_3_/Bi_2_O_3_ nanocomposites enhanced the deposition-stripping process, resulting in improved electrocatalytic activity compared to other modified electrodes. The Fe_2_O_3_/Bi_2_O_3_/GCE facilitated the simultaneous detection of Cd^2+^ and Pb^2+^ at nanomolar concentrations, exhibiting a dynamic range of 0.002–4 μM.^437^ Additionally, an electrochemical sensor utilizing UiO-66 was developed for the concurrent measurement of these heavy metals, demonstrating a linear detection range of 10–50 μg L^−1^ and detection limits of 1.16 μg L^−1^ for Cd^2+^ and 1.14 μg L^−1^ for Pb^2+^. The carbonized UiO-66/Bi/GCE electrode showcased remarkable detection capabilities, attributed to the increased availability of active sites for Bi and the co-deposition of heavy metal ions, which enhanced electron transport. Furthermore, a decorating method applied to disposable SPCEs yielded detection limits of 4.67 μg L^−1^ for Cd^2+^ and 1.24 μg L^−1^ for Pb^2+^, with a linear detection range of 20–120 μg L^−1^.^438^

### 4.3 Veterinary drug residues

Animal husbandry practices have led to a decrease in disease prevalence and an increase in animal welfare. In this context, the use of antibiotics plays a significant role. However, a lack of awareness regarding proper medical practices and the overuse of these drugs has resulted in antibiotic residues being present in milk, meat, eggs, and honey. These residues pose serious health risks, including mutagenicity, teratogenicity, carcinogenicity, antimicrobial resistance, hypersensitivity reactions, and disruptions to the internal microbiota. While most antibiotics administered to animals are metabolized and excreted, traces that remain in animal tissues can still lead to health issues if ingested.^439^ Bacteria gain antibiotic resistance through mutations. After developing antibiotic resistance, bacteria use various self-protection mechanisms, including disrupting the cell wall and nucleic acid synthesis, inhibiting protein synthesis, and changing the outer membrane to alter permeability.^440^ Several antibiotics are commonly used, including tetracycline, β-lactamase inhibitors, penicillin, sulfonamides, aminoglycosides such as streptomycin, and macrolides like erythromycin. Oxytetracyclines are frequently used to treat animal infections. Overuse causes accumulation in milk, pork, eggs, and chicken, posing health hazards.^441^ While Staphylococcus infections are treated with cloxacillin, a penicillin resistant to β-lactamases, residual microbial diseases in domestic animals may contaminate milk because animal-derived products include varied antibiotic residues that are harmful to humans.^442^

#### 4.3.1 Detection of veterinary drug residues

Huang *et al.* developed a flexible electrode device called Co nanoparticle-modified 3D N-doped porous carbon (Co@3D NPC) by integrating a Co-based metal–organic framework (ZIF-67) with polyimide (ZIF-67/PI) using a room-temperature laser ablation technique.^445^ This Co@3D NPC electrode exhibited improved electron transport, enhanced physical adsorption, and a larger electrochemically active surface area compared to 3D NPC electrodes made solely from polyimide film. Additionally, the Co@3D NPC electrode technology proved effective in detecting two widely used veterinary drugs and insecticides, clozapine and albendazole, achieving detection limits of 2.8 nM and 2.5 nM, respectively, along with detection sensitivities of 410.47 μA μM^−1^ cm^−2^ for clozapine and 580.33 μA μM^−1^ cm^−2^ for albendazole. The Co@3D NPC electrode device exhibited remarkable stability and precision, highlighting its potential for practical applications.^443^ Promethazine (PHZ), a sedative used in veterinary medicine, poses potential risks to humans. Detecting PHZ through electrochemical methods presents a practical solution; however, conventional electroanalytical techniques face challenges when applied directly to meat samples due to matrix interference. In a pioneering study, Yang *et al.* achieved sensitive and selective detection of PHZ in beef and beef liver using differential pulse voltammetry (DPV) combined with magnetic solid-phase extraction. They developed a magnetic adsorbent by coating CoFe_2_O_4_/graphene with C18-functionalized mesoporous silica (MG@mSiO_2_-C18), which effectively isolates PHZ, minimizing the influence of contaminants and concentrating the analyte on the magnetic electrode. The modified N-doped hollow carbon microspheres (HCM) further enhance the electrochemical signal of PHZ. This integrated detection method demonstrates a low detection limit of 9.8 nmol L^−1^ and a wide linear range from 0.08 μmol L^−1^ to 300 μmol L^−1^, with promising recovery rates in beef samples, indicating its potential for rapid and efficient identification of PHZ in meat products.^444^

Dimetridazole (DMZ), a derivative of nitroimidazole, has been utilized as an antibiotic for treating bacterial and protozoal infections in poultry. However, the presence of DMZ residues poses health risks to humans. To enhance the detection of DMZ, Behera and colleagues developed a novel electrocatalyst. They created a Cu-integrated poly(aniline) (PANI) electrocatalyst, termed PANI-Cu@BSA, through a one-step biomimetic mineralization and polymerization process that employed bovine serum albumin (BSA) as a stabilizing agent. This synthesized PANI-Cu@BSA was further encapsulated with rGO using ultrasonication. The resulting nanocomposite, PANI-Cu@BSA/rGO, exhibited remarkable electrical conductivity, water dispersibility, and effective nanoscale particle performance. A screen-printed carbon electrode modified with the PANI-Cu@BSA/rGO nanocomposite was then utilized for the sensitive electrochemical detection of DMZ. Compared to both the PANI-Cu@BSA/rGO/SPCE and the bare SPCE in phosphate-buffered saline, the modified electrode exhibited a significantly higher current intensity. The combination of PANI-Cu@BSA and rGO facilitated the formation of analyte–electrode junctions characterized by high conductivity and increased active surface areas, thereby enhancing electron transfer between the electrode and the analyte. The PANI-Cu@BSA/rGO/SPCE demonstrated linear detection ranges of 1.78 nM, a sensitivity of 5.96 μA μM^−1^ cm^−2^, and a detection limit that underscores its effectiveness for analytical applications.^445^ Furaltadone (FTD), a nitrofuran derivative used in veterinary medicine, poses environmental and health risks due to the toxic nature of its organic components. To facilitate the electrochemical detection of FTD, an activated SPCE was modified with hydrothermally synthesized CuCoO_2_, employing CV and DPV. This modified SPCE/CuCoO_2_ electrode demonstrated a broad linear detection range of 0.1–316 μM, significantly improved sensitivity of 21.03 μA μM^−1^ cm^−1^, and a detection limit of 1.79 nM.^446^

Veterinary medicine employs the broad-spectrum antibiotic tetracycline (TC) to address various health issues; however, the presence of its residues in food and beverages raises significant concerns. This necessitates the development of an efficient, cost-effective, and straightforward method for detecting TC in aquatic environments. Wang *et al.* introduced a nanocomposite sensor for tetracycline detection, utilizing bismuth carbide (BiC) nanofibers modified with AuNPs and a TC aptamer. The incorporation of AuNPs enhances electrochemical detection and facilitates the binding of the TC aptamer. The electrochemical performance of the BiC@Au@Apta sensor was assessed using CV and DPV, revealing a detection range of 0.001–100 μM and LOD of 2.92 nM under optimal conditions, along with impressive stability (*I* = 97.30%), repeatability, and resistance to interference. Additionally, antimicrobial resistance (AMR) poses a significant socioeconomic challenge to public health, with β-lactams being the most frequently prescribed treatments for various bacterial infections. The routine use of antibiotics contributes to the emergence of AMR in both humans and animals. In response, Kolhe *et al.* developed an electro-immunosensor designed to detect and mitigate antibiotic misuse in food products derived from animals. This sensor utilized a recessed nano-disk array electrode (RNE) created from a PEO-*b*-PMMA amphiphilic block copolymer, which was subsequently immobilized with Penicillin and Cefalexin antibodies. The RNE working electrode demonstrated limits of detection of 14.8 and 13.8 pM for penicillin and cefalexin, respectively, showcasing strong selectivity against non-specific antibiotics.^447^

### 4.4 Food additives

Food additives are deliberately incorporated into food products to fulfill specific technological or sensory roles that improve the quality of these items, as long as they remain within legally established limits. Any substance that is meant to modify the properties of food, whether directly or indirectly, and thus becomes part of the food itself, is categorized as a food additive.^448,449^ To be recognized as such, a substance must be deemed safe for its intended use by qualified experts based on scientific evidence. However, many synthetic preservatives, including sulfites, benzoates, sorbates, and nitrates, raise health concerns due to their potential harmful effects.^450^ While these artificial preservatives are effective in preventing spoilage and prolonging shelf life, the impact of natural preservatives, such as nisin, on gut microbiota remains under-researched. Research has shown that certain dietary additives can trigger inflammation and worsen metabolic diseases by altering gut microbiota,^449^ particularly affecting vulnerable populations such as children, who are more susceptible to the adverse effects of these additives. Therefore, it is essential to investigate further the significant influence of food additives on gut microbiota, potentially utilizing *in vitro* models of the human gastrointestinal system.^451^ Some additives, like monosodium glutamate (MSG) and artificial sweeteners, have been associated with addictive properties that may influence eating behaviors, resulting in compulsive consumption and overeating due to their impact on brain function.^452^ The ongoing debate regarding the health implications of food additives is fueled by inconsistent research findings on their toxicity and effects. Nonetheless, these additives must be used within legal limits, raising concerns about their proper identification and regulation.

#### 4.4.1 Detection of food additives

The excessive reliance on flavor enhancers in food marketing diminishes the nutritional integrity of products. Vanillin (VAN), a widely used flavor enhancer, necessitates accurate detection methods. This study introduces an electrochemical sensor utilizing a modified electrode made from La_2_NiO_4_-functionalized carbon nanofibers (f-CNF) to identify VAN in food samples. Through electrochemical analysis employing CV and DPV, the sensor exhibited a sensitivity of 0.2899 μAμM^−1^cm^−2^ and LOD of 6 nM. Testing with food samples confirmed the modified electrode's effective electrocatalytic properties for VAN detection, achieving a high recovery rate.^453^ Taouri *et al.* conducted measurements of VAN in food samples utilizing an electrochemical sensor.^457^ The sensor incorporated a carbon paste electrode featuring a fullerene (FNT) nanostructure along with functionalized multi-walled carbon nanotubes. For the electrochemical analysis, cyclic voltammetry was applied after optimizing parameters such as pH, accumulation time, and supporting electrolytes. Following this optimization, the nanostructured sensor demonstrated a detection limit of 3.4 × 10^−8^ mol L^−1^, with linear responses observed between 5 × 10^−8^ and 9 × 10^−6^ mol L^−1^ at trace levels, and from 10^−5^ to 10^−4^ mol L^−1^ for higher concentrations. Notably, VAN was removed entirely after 300 seconds of adsorption at concentrations below 10^−5^ mol L^−1^.^454^ Nehru *et al.* developed a hydrothermal method to synthesize g-C_3_N_4_NTs@MoS_2_ and evaluated its electrochemical performance for VAN detection. This sensor was engineered to produce a stronger current signal by utilizing various electrolytes and optimizing its settings. Under ideal conditions, the sensor demonstrated sensitivity of 3.48 μA μM^−1^ cm^−2^, limit of detection (4 nM), and extensive linear ranges (0.005–458.9 μM) using DPV.^455^

Sebastian *et al.* investigated the electrochemical detection of tert-butylhydroquinone (TBHQ), a potentially harmful food preservative.^456^ They employed sonochemical techniques to develop a nanocomposite by combining β-cyclodextrin (β-CD)-functionalized carbon black with various metal oxides, such as ZnO, CuO, and MgO. The resulting nanocomposite underwent extensive characterization through multiple methods. Notably, the screen-printed carbon electrode-based nanocomposite exhibited remarkable electrocatalytic performance for TBHQ detection, achieving a sensitivity of 22.67 μA μM^−1^ cm^−2^ and a detection limit of 1 nM. In a related study, Balram *et al.* developed an electrochemical sensor for TBHQ by sonochemically integrating spinel Co_3_O_4_ nanorods with surface-functionalized carbon black (FCB), resulting in a hybrid nanocomposite. The synthesis involved co-precipitation with oxalic acid to create highly porous Co_3_O_4_ nanorods, while acid treatment was employed to oxidize the carbon black. The modified electrode exhibited excellent electrochemical performance, allowing for TBHQ detection with a sensitivity of 7.94 μA μM^−1^ cm^−2^ and a detection limit of 1 nM.^457^

**Fig. 14:**
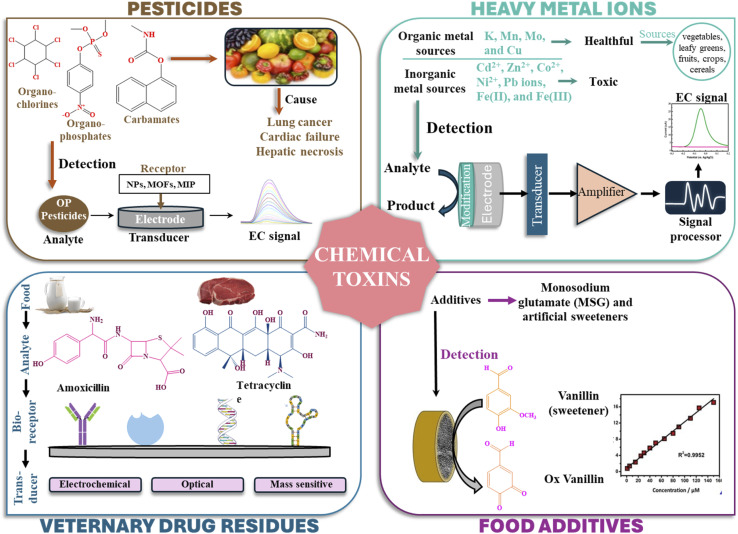
Chemical toxins and their identification through various biosensing technologies.

Rajaji and his team utilized traditional solvothermal and hydrothermal methods to produce binary nanosheets of Bi_2_Te_3_. To enhance electrocatalytic performance, they synthesized Bi_2_Te_3_/g-C_3_N_4_ binary nanosheets *via* a hydrothermal process. The electrochemical characteristics of the modified GCEs featuring Bi_2_Te_3_/g-C_3_N_4_ BNs were assessed using DPV, EIS, and CV. The Bi_2_Te_3_/g-C_3_N_4_ BNs electrode demonstrated high sensitivity and a low detection limit in identifying ractopamine in food samples.^458^ Nejad *et al.* designed a screen-printed graphite electrode (SPGE) incorporating MnO_2_ nanorods within a graphene oxide nanocomposite (MnO_2_ NRs/GO) for the detection of sunset yellow. Electrochemical techniques, including chronoamperometry, DPV, CV, and LSV, confirmed the effective oxidation of sunset yellow on the MnO_2_ NRs/GO/SPGE. The CV results indicated that the MnO_2_ NRs/GO nanocomposite exhibited electrocatalytic activity, with the electrode showing a linear response for sunset yellow concentrations ranging from 0.01 to 115.0 μM under optimal DPV conditions, achieving an LOD of 0.008 μM. Fig. 14 illustrates the identification of chemical toxins through various biosensing technologies, while Table 9 summarizes the detection of pesticides, heavy metals, veterinary drug residues, and food additives using diverse analytical methods.

**Table 9:** Detection of pesticides, heavy metals, veterinary drug residues, and food additives by electrochemical methods

Sr No.	Method	Analyte	Electrode modifier	Linear range	LOD	Sample/recovery	Ref.
1	DPV	Organophosphate pesticides (Ops)	TMB-MnNS-ATCh-AChE	0.1–20 ng mL^−1^	0.025 ng mL^−1^	Pakchoi/99.72–102.41%	417
2		Pesticides (Ops)		3–4000 ng mL^−1^	1.2 ng mL^−1^		418
3	EC	Crisquat	rGO@AgWO_4_/GCE	1–1108 μM	0.0661 μM		419
4		Imidacloprid (IMC) pesticide		10.80–195.50 μM	0.257 μM		420
5	EC	Monocrotophos pesticide	ZnONFs/rGO/Au	0.01–100 nM	0.01 nM		421
6	DPV	Paraquat	CuMOF/rGO/Au	0.30–5.00 μmol L^−1^	50.0 nmol L^−1^	River, tap water and human blood serum/98–104%	423
7	SWV	Imidacloprid (IMD)			0.97 μM	Environmental water/90.0–105.0%	424
8	EC	Carbendazium (CBZ)	N-CNHs/PEI-CNTs/GCE	15 nmol L^−1^–70 μmol L^−1^	4 nmol L^−1^	Water/87.33–117.67%	425
9	EC	Dichlorvos	ChOx/PBCB_ethaline_-HNO_3_^PTD^/MWCNT/GCE	0.8–30 μM	1.59 nM	Orange juice/99.7–103.2%	459
10	CV	Profenofos	BSA/Apt/rGO-CuNPs/SPCE	0.01–100 nM	3 pM	River water/80–92%	460
		Phorate		1–1000 nM	300 pM		
		Isocarbophos		0.1–1000 nM	30 pM		
		Omethoate		1–500 nM	300 pM		
11	CV	Diazinon (DZN)	DZN/thiolated aptamer/AuNPs/SPGEs	0.0304–304 ng mL^−1^	0.005 ng mL^−1^	Plasma of Male Wistar rat/96.0–99.30%	461
	EIS						
12	SWV	Sulfentrazone (SFZ)	SPE-MWCNT	1.0–25 μmol L^−1^	0.8 μmol L^−1^	Soy milk/98.67%	462
						Groundwater/99.00%	
13	Electrodeposition	Heavy metal ions (HMIs)	Mo-WO_3_/CC	0.1–100.0 μM	11.2–17.1 nM	—	429
14	d-μ-SPE	Heavy metal ions (Cd^2+^ and Pb^2+^)	—	0.3–90 μg L^−1^	0.09 ng mL^−1^	Food and soil	463
				6–120 μg L^−1^			
					1.9 ng mL^−1^		
15	SWASV	Cd(ii)	BCN-Nafion/GCE	1–150 μg L^−1^	0.41 μg L^−1^	*Beta vulgaris* var.*Cicla* L/101.56–104.81%	431
				2–150 μg L^−1^	0.93 μg L^−1^		
		Pb(ii)					
		(HMIs)					
16	DPSV	HMIs				Milk/96.0–104.3%	432
17	CV	Zn^2+^	FGP/AuNC/GCE	6–7000 μg L^−1^	0.08 μg L^−1^	Peanut/98.5% (Zn^2+^), 105% (Cd^2+^), and 104% (Pb^2+^)	433
				4–6000 μg L^−1^	0.09 μg L^−1^	Rape bolt/94.5% (Zn^2+^), 105% (Cd^2+^), and 101% (Pb^2+^)	
				6–5000 μg L^−1^	0.05 μg L^−1^	Tea/99.0% (Zn^2+^), 102% (Cd^2+^), and 102% (Pb^2+^)	
	EIS	Cd^2+^					
		Pb^2+^					
18	CV	Cu^2+^	FGP/AuNC/GCE	4–4000 μg L^−1^	0.19 μg L^−1^	Peanut/104% (Cu^2+^) and 95.5% (Hg^2+^)	433
				6–5000 μg L^−1^	0.01 μg L^−1^		
	EIS	Hg^2+^				Rape bolt/102%(Cu^2+^) and 104% (Hg^2+^)	
						Tea/93.5%(Cu^2+^) and 101% (Hg^2+^)	
19	DPV	Pb^2+^	*p*-PDMS@MSF/ITO			Fruit juice/	434
		Cd^2+^				Beverage/	
20	EC	Cd^2+^	UiO-66-NH_2_@MWCNTs/GCE	0.5–170 μg L^−1^	0.2 μg L^−1^	Meat/95.1–107.5%	435
21	SWASV	Pb(ii)	rGO/MoS_2_/CS/GCE	0.005–0.05 μM	0.0016 μM	Tobacco leaves	436
22	SWASV	Pb(ii)	Fe_2_O_3_/Bi_2_O_3_/GCE	0.002–4 μM		Water and milk	437
		Cd(ii)					
23	CV	Cd(ii)	UiO-66/Bi/GCE	10–50 μg L^−1^	1.16 μg L^−1^	Tap water/97% (Cd^2+^) and 105%(Pb^2+^)	438
	EIS	Pb(ii)			1.14 μg L^−1^	The Songhua river/93% (Cd^2+^) and 91%(Pb^2+^)	
24	CV	Cd(ii)	UiO-66/Bi/GCE	20∼60 μg L^−1^	1.73 μg L^−1^	Tap water/96% (Cd^2+^) and 93%(Pb^2+^)	438
	EIS	Pb(ii)			1.00 μg L^−1^	The Songhua river/94% (Cd^2+^) and 95%(Pb^2+^)	
25		Clozapine			2.8 nM		443
		Albendazole			2.5 nM		
26	CV	Promethazine (PHZ)	MG@mSiO_2_-C_18_-HCM/MGCE	0.08–300 μmol L^−1^	9.8 nmol L^−1^	Beef/82.5–97.2%	444
	DPV					Beef liver/81.5–96.1%	
27	LSV	Dimetridazole (DMZ)	PANI-Cu@BSA/rGO/SPCE	0.79–2057 μM	1.78 nM	Human blood serum/90.0–100.0%	445
						Rat blood serum/98.0–101.0%	
						Egg/	
						Egg/97.0–100%	
28	CV	Furaltadone (FTD)	ASPCE/CuCoO_2_	0.1–316 μM	1.79 nM	Antibiotic 1/90–93%	446
	DPV						
29	CV	Tetracycline (TC)	BiC@Au@Apta/AuE	0.001–100 μM	2.92 nM	Tap water/97.52–125.18	464
						Milk/78.73–103.80	
	EIS						
	DPV						
30	Fluorescent	Tetracycline (TC)	—	0.1–100 μM	0.23 μM	Antibiotics	465
31	SERS	Tetracycline (TC)	CoNi-ZIFs@Ag@NF	10^−10^–10^−5^ M	1.0 × 10^−11^ M	—/94.45–114.25%	466
32	DPV	Dimetridazole (DMZ)	Mn–SnO@rGO/GCE	0.009–1291 μM	2.0 μM	Milk and egg	467
33	DPV	Dimetridazole (DMZ)	Se–Co_3_O_4_@GO-NC/GCE	0.02–83.72 μM	3.4 nM	Milk/98.5%	468
						Pigeon egg/99.6%	
						Pigeon meat/100.4%	
34	DPV	Dimetridazole (DMZ)	DM/Nd_2_Sn_2_O_7_/GCE	0.01–1453 μM	6.0 nM	Human blood serum and human urine	469
35	CV	Vanillin	La_2_NiO_4_/f-CNF/GCE		6 nM	Chocolate/	453
	DPV					Ice cream/	
36	CV	Vanillin	f-MWCNTs-FNTs/CPE	5 × 10^−8^–9 × 10^−6^ mol L^−1^	3.4 × 10^−8^ mol L^−1^	Sugar/100–107%	454
37	DPV	Vanillin	g-C_3_N_4_ NTs@MoS_2_/GCE	0.005–458.9 μM	4 nM	—	455
38	CV	*Tert*-butyl hydroquinone (TBHQ)	Co_3_O_4_ NRs/FCB/SPCE		1 nM	Beef tallow/	457
						Peanut oil/	
						Lake water/	
	DPV						
39	DPV	Ractopamine (RAC)	Bi_2_Te_3_/g-C_3_N_4_ BNs/GCEs	0.015–456.4 μM	1.07 nM	Pork, beef, and chicken	458
	EIS						
	CV						
40	DPV	Sunset yellow	MnO_2_ NRs/GO/SPGE	0.01–115.0 μM	0.008 μM	Apple juice and orange juice/97.3–104.6%	470
	CV						
	LSV						

Reported literatures predominantly focus on sensor performance in controlled environments; however, it is crucial to comprehend the physical and chemical interactions that influence sensor effectiveness within complex food matrices. Each sensor type has specific known interferences from the matrix, such as lipid and protein fouling in electrochemical sensors, and background fluorescence or quenching in optical techniques. The current document discusses typical sample preparation methods necessary for achieving low detection limits, while also addressing common causes of false positives and negatives. It presents the validation levels as reported by the original authors, which may include buffer-only limits of detection, multi-matrix laboratory validations, inter-laboratory assessments, or field pilot studies. To emphasize the applicability of the data, a specific column for sample recovery has been incorporated into the tables, connecting the reported sensors to practical applications.

## 5. Future prospects and conclusions

The food toxin detection field is advancing thanks to innovations in sensor technology, including biosensors and chromatographic sensors. These improvements in sensitivity, specificity, and response speed, along with portable, user-friendly devices, drive this progress. Biosensors, leveraging biological recognition elements like enzymes, antibodies, and nucleic acids, now incorporate nanomaterials such as graphene and gold nanoparticles to enhance signals and lower detection limits. Flexible and wearable biosensors enable continuous, real-time food monitoring and early contamination alerts. Their integration with smartphones further makes detection and data analysis accessible to non-experts, boosting practical use.

Recent years have seen significant progress in chemical sensors, driven by innovative materials like metal–organic frameworks and covalent organic frameworks that improve sensitivity and selectivity through high surface area and tunable porosity. Molecularly imprinted polymers offer cost-effective, precise toxin detection *via* tailored binding sites. The move toward miniaturized sensors expands their use in food safety, enabling quick on-site testing. Advances in electrochemical sensors, improved electrode materials, and the addition of nanomaterials like carbon nanotubes boost sensitivity and response times. Combining electrochemical sensors with microfluidics allows for simultaneous multi-toxin detection, simplifying analysis and reducing reagent use. Self-powered sensors, powered by environmental energy or the sample itself, promise sustainable, maintenance-free operation, thereby boosting reliability and versatility in food toxin detection.

Fluorescent sensors are increasingly recognized for their sensitivity and quick response times. Fluorescent nanomaterials like quantum dots and carbon dots have enhanced these sensors with bright, stable signals. Recent advancements include ratiometric sensors using dual-emission probes to improve accuracy and reduce environmental interference. Integrating fluorescent sensors with microfluidic chips allows high-throughput food sample screening, streamlining detection. Optical sensors utilize techniques like surface plasmon resonance (SPR), surface-enhanced Raman scattering (SERS), and interferometry, with recent focus on boosting sensitivity and lowering detection limits. SPR utilizes nanostructured materials to amplify signals for the detection of low-level toxins, whereas SERS relies on molecular spectral fingerprints for the precise identification of contaminants. Coupling these sensors with portable devices like smartphones enables on-site, real-time food testing. Spectroscopic sensors have evolved with advanced materials and signal enhancement, using mid-IR quantum cascade lasers for increased sensitivity, facilitating quick, non-destructive toxin detection based on spectral characteristics. Combining spectroscopic sensors with chemometrics improves data analysis, allowing detection of multiple toxins simultaneously. Chromatographic sensors remain vital for their separation efficiency and accuracy, with recent miniaturization of systems like microchip chromatography reducing analysis time and sample volume. Coupling chromatographic sensors with mass spectrometry offers sensitive, specific toxin identification. Automated, high-throughput systems further improve toxin screening efficiency.

Recent advancements in the integration of machine learning with nanosensing technologies have revealed significant advantages alongside distinct challenges. Supervised learning algorithms, such as support vector machines, random forests, and gradient-boosted trees, have proven effective in classifying intricate signal patterns from multi-channel sensors, enhancing selectivity by distinguishing target toxins from potential interferents. Meanwhile, unsupervised techniques like principal component analysis and *t*-SNE aid in reducing dimensionality and exploring clustering within spectral or multi-electrode datasets, thereby revealing concealed interference patterns. Additionally, deep learning approaches, including convolutional and recurrent neural networks, have been utilized on raw spectral or time-series data to identify features that traditional methods might miss, thus broadening detection capabilities in noisy environments.

Practical challenges in sensor studies remain significant. Data scarcity and class imbalance often result in biased models due to the limited number of positive samples, necessitating strategies such as targeted data augmentation, synthetic data generation through physics-based simulations, and transfer learning from similar analytes. Additionally, label noise and inconsistent protocols, stemming from variations in sample preparation and inter-laboratory discrepancies, compromise model generalizability; addressing this requires standardized protocols, matrix-matched training sets, and robust algorithms that can tolerate label noise. Furthermore, sensor drift and batch variability can diminish model performance over time, which can be mitigated through online calibration, domain adaptation techniques, and periodic retraining with calibration standards. Lastly, deployment constraints due to limited computing power on portable devices necessitate the use of lightweight models, model compression, and edge-optimized inference frameworks. Systematic efforts to address these issues are essential for advancing machine learning from theoretical concepts to practical, field-ready enhancements in nanosensor technology.

When interpreting reported LODs, it is crucial to take into account the complexity of the matrix, the difficulties associated with sample preparation, and the validation phase. Sensors that present LODs solely under buffer conditions require further laboratory validation and field testing to be considered appropriate for regulatory use. The current document connects theoretical performance with these practical aspects, providing essential context for assessing translational readiness without necessitating the replication of experimental data.

In conclusion, the future of food toxin detection is poised for remarkable advancements driven by innovations in sensor technology. The ongoing enhancement of various sensor types, including biosensors, chemical, electrochemical, fluorescent, colorimetric, optical, spectroscopic, and chromatographic sensors, holds significant potential to improve food safety and public health. These technological improvements enable the more sensitive, specific, and rapid identification of toxins, allowing for proactive strategies to mitigate foodborne illnesses and safeguard consumers. As the field evolves, the integration of these sophisticated sensors into portable, user-friendly devices, along with the utilization of artificial intelligence for data analysis, will further increase their practicality and accessibility. This review highlights the vital role of advanced sensor technologies in protecting public health. It underscores the need for ongoing research and development to address the evolving challenges in food safety. By adopting these innovations, researchers, industry stakeholders, and regulatory agencies can work together to strive for a safer and more secure global food supply chain.

## Conflicts of interest

Authors declare no conflict of interest.

## Data Availability

No primary research results, software or code have been included and no new data were generated or analysed as part of this review.
